# Accelerated Porosity Screening Using a Multichannel Colorimetric Array

**DOI:** 10.1002/anie.202510400

**Published:** 2025-08-21

**Authors:** Yushu Han, Isaiah Borne, Biplab Dutta, Rob Clowes, Hang Qu, Alex James, Charlotte E. Boott, Marc A. Little, Andrew I. Cooper

**Affiliations:** ^1^ Department of Chemistry and Materials Innovation Factory University of Liverpool 51 Oxford Street Liverpool L7 3NY UK; ^2^ Institute of Chemical Sciences Heriot‐Watt University Edinburgh EH14 4AS UK

**Keywords:** Colorimetry, Dye adsorption, Gas adsorption, High throughput, Porosity

## Abstract

Porous materials are important for many technologies, but the measurement of porosity by gas adsorption isotherms is slow, taking around one day per sample using a single‐port gas sorption analyzer, even when using a “quick” analysis method with relatively few data points. With the increased use of automated platforms for material generation, porosity analysis is now frequently the bottleneck in the discovery of new porous materials. Here, we present a semiautomated pre‐screening strategy that uses dye adsorption to create a colorimetric array that is combined with computer vision analysis for porosity screening. By using a six‐dye multichannel array and a defined porosity threshold, our method rapidly screened 50 candidate materials that spanned molecular solids, polymers, and metal–organic frameworks. The method showed a 98–100% classification accuracy compared with gas uptake measurements. While this method is more qualitative than quantitative, it is more than 30 times faster than conventional gas sorption measurements, and it has the scope to be made much faster with greater parallelization and automation. This makes this colorimetric method suitable for pre‐screening arrays of materials to choose samples that merit more detailed conventional porosity analysis.

## Graphical abstract

A high‐throughput porosity screening method was developed for rapid porosity evaluation, offering a cost‐effective and scalable approach to identifying candidate porous materials that is 30 times faster than conventional gas sorption methods with 98–100% classification accuracy. A multichannel six‐dye array was used as a proxy measurement for porosity, eliminating false results that would result from the use of a single dye.

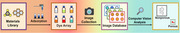

## Introduction

Automated workflows integrating synthesis, characterization, and computer simulations are becoming increasingly common in materials discovery.^[^ ^1^ ^]^ By enabling the rapid collection of datasets, automated workflows can facilitate the identification of optimal candidate materials from large chemical search spaces, sometimes with much greater efficiency than conventional experimental planning and design of experiments. For example, robotic‐infused automated workflows have been shown to accelerate library synthesis,^[^ ^2^ ^]^ optimize reaction yields,^[^ ^3^, ^4^ ^]^ as well as to discover and optimize electrolyte formulations,^[^ ^5^, ^6^ ^]^ photocatalysts,^[^ ^7^, ^8^ ^]^ and perovskites for solar cells.^[^ ^9^ ^]^ All closed‐loop materials discovery workflows involve some property measurement to allow the direct or indirect detection of “hits”.^[^ ^8^ ^]^ Analytical techniques that have been incorporated into high‐throughput workflows include UV–vis spectrometry,^[^ ^1^ ^]^ powder X‐ray diffraction (PXRD),^[^ ^10^, ^11^ ^]^ FTIR spectrometry,^[^ ^12^, ^13^ ^]^ gas chromatography (GC),^[^ ^14^, ^15^ ^]^ mass spectrometry (MS),^[^ ^16^, ^17^ ^]^ and computer vision which has aided continuous color monitoring or object detection of chemical reactions where reaction progress is linked to visual clues.^[^ ^18^, ^19^ ^]^ Such methods often support parallel testing through the use of either multiwell plates or continuous injection. However, not all techniques can be automated and seamlessly integrated into such workflows,^[^ ^20^ ^]^ and gas adsorption measurements are one such example.

Porous materials, including amorphous polymers, discrete molecular cages, and crystalline frameworks, can have a wide range of structures and pore architectures. The functional properties of these materials stem from their permanent porosity, leading to applications such as storage,^[^ ^21^, ^22^, ^23^ ^]^ separation,^[^ ^24^, ^25^, ^26^ ^]^ and catalysis.^[^ ^27^, ^28^ ^]^ The level of porosity can vary greatly—for some applications, such as storage, pore volume is important, whereas for separations, pore size is more critical. A metal–organic frameworks (MOFs), NU‐110E,^[^ ^29^ ^]^ shows the high reported surface area (7140 m^2^ g^−1^) with an exceptionally large total pore volume of 4.40 cm^3^ g^−1^. Achieving such structures is not always straightforward; for example, incipient porosity is often lost upon desolvation in many materials. Porosity is relatively rare: in 2017, the Cambridge Structure Database contained 54808 nondisordered MOF structures, and about 85% of these structures had gravimetric surface areas equal to zero.

The current bottleneck in discovering materials with permanent porosity often lies in the characterization step since for many materials, our ability to synthesize libraries has outpaced our ability to measure their porosity. Various complementary porosity characterization techniques,^[^ ^31^ ^]^ such as nuclear magnetic resonance,^[^ ^32^ ^]^ mercury intrusion porosimetry,^[^ ^33^ ^]^ and X‐ray computed microtomography^[^ ^34^ ^]^ have been used to analyze materials with diverse porosity characteristics. However, for micropore and mesopore analysis, gas adsorption is by far the most widely used method because of its nondestructive nature, accuracy in surface area measurement, and applicability across a broad range of materials. Recent innovations have aimed to improve sample throughput for gas sorption measurements. Bakken et al.^[^ ^35^ ^]^ developed an instrument that allows for the parallel analysis of eight samples in breakthrough experiments, and Mason et al.^[^ ^36^ ^]^ introduced an adsorption analyzer with 28 independent channels for gas dosing and analysis. However, these methods are still somewhat labor‐intensive and have a high barrier‐to‐entry since they require: i) skilled manual sample preparation; ii) precise degassing (6–24 h) at high temperature under high vacuum; and iii) stable introduction of adsorbate gases at low temperatures (e.g., N_2_ at 77 K) and ultralow pressures (<10^−5^ mmHg). These stringent requirements hinder the integration of such fast gas adsorption into automated robotic workflows, where there is a growing need for rapid porosity screening.

**Figure 1:**
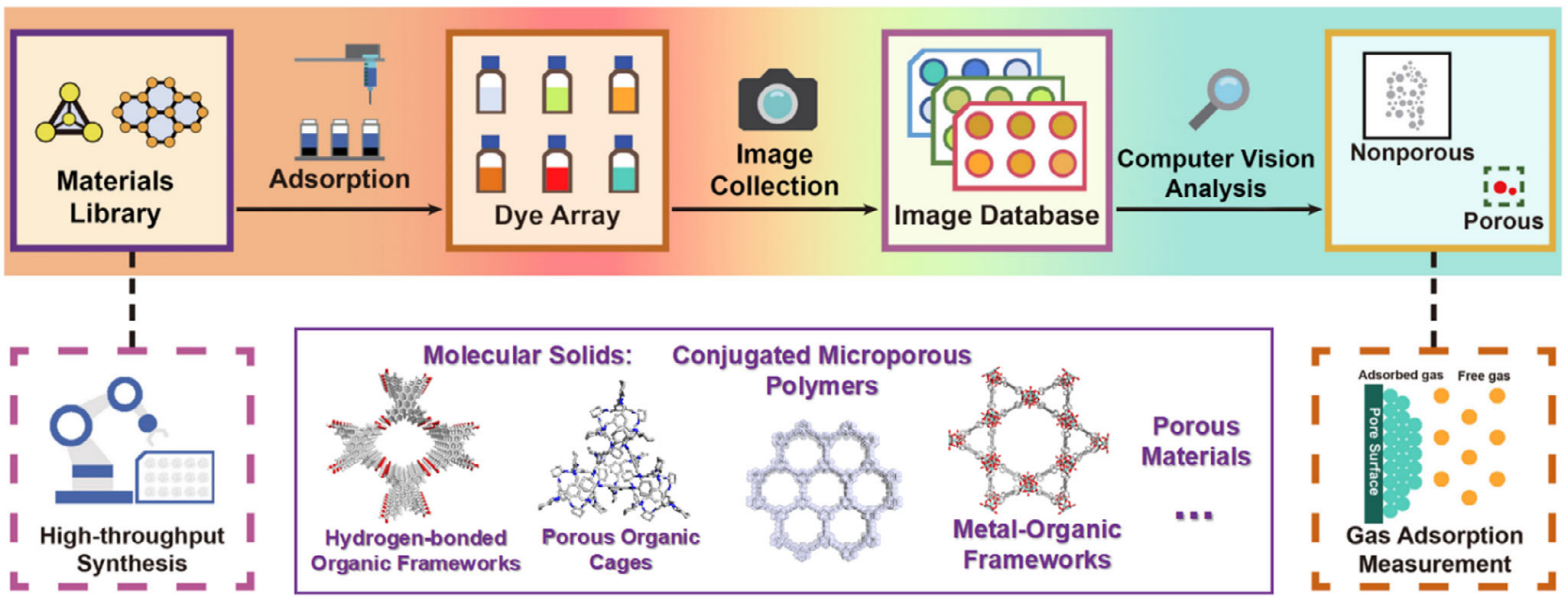
Scheme showing workflow for high‐throughput porosity screening by colorimetry. (Top) The key stages in the workflow are materials collection, dye adsorption, building of an image database, and colorimetric analysis of the image array. This workflow was designed for integration with a high‐throughput synthesis station and subsequent validation of “hits” using conventional gas adsorption measurements. (Bottom) The four classes of materials screened in this study; molecular solids (e.g., porous organic cages, hydrogen‐bonded organic frameworks), conjugated microporous polymers (CMPs), and MOFs.

In this study, we developed a high‐throughput porosity screening method that employs a multichannel colorimetric dye sorption array for the rapid assessment of porous materials (Figure 1). The porosity evaluation is determined qualitatively by the extent of color fading of the dye solution, which results from the adsorption of the dye molecules in the porous structure of the material. By using colorimetry, samples are classified as either porous or nonporous based on whether they meet a predefined threshold. To validate this approach, we used the array to classify the porosity of a diverse material library, including molecular solids (e.g., hydrogen‐bonded organic frameworks, porous organic cages (POCs)), conjugated microporous polymers (CMPs), and MOFs. To streamline the workflow and eliminate human error, this method used a computer vision‐based color analysis with robotic automation, allowing for the simultaneous evaluation of multiple samples under controlled conditions. This easy‐to‐use screening method offers an approach to rapidly identify promising samples before carrying out more traditional gas adsorption measurements. Benchmarking showed that the method, while qualitative, is more than 30 times faster than analysis using a conventional single‐port gas sorption analyzer.

## Results and Discussion

### The Design of the Dye Array

**Figure 2:**
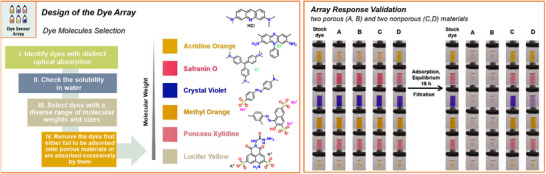
Design and validation of the multichannel dye array. (Left) Four fundamental criteria were used to guide the selection of dye molecules for the dye array design; the structures of selected dye molecules are shown (Acridine orange, Safranin O, Crystal violet, Methyl orange, Ponceau xylidine, and Lucifer yellow), ordered by increasing molecular weight. (Right) Images showing the adsorption behaviors of four model materials (two porous activated carbons (A, B) and two non‐porous linear polymers, polycaprolactone and Tenax® (C, D), used to validate the color response of the designed array. Red squares indicate the area where the solution color was assayed.

Porosity evaluation using the adsorption of polar liquids (e.g., ethylene glycol monoethyl ether) and/or dye solutions (e.g., methylene blue) has been employed to infer the specific surface area of clays and clay‐like soils, as well as the pore network connectivity of activated carbons.^[^ ^37^ ^]^ Like gas adsorption, liquid adsorption is a mass transfer process in which solute molecules (adsorbates) move from the liquid phase to form a surface monolayer on the solid materials (adsorbents), followed by multilayer adsorption if there is sufficient pore space.^[^ ^38^ ^]^ With many porous materials, the extensive surface area and accessible pore volume provide abundant sites for adsorption. Dye molecules can adsorb strongly and reversibly onto the material's surface due to various interactions such as hydrogen bonding, electrostatic interactions, and π–π stacking, in addition to van der Waals forces.^[^ ^39^, ^40^ ^]^ Among the multiple factors influencing dye adsorption on porous materials,^[^ ^41^ ^]^ molecular size plays a crucial role, as it directly affects the accessibility and diffusion of dye molecules within the pore structure. As such, the compatibility between dye molecule size and pore size determines the extent of adsorption, with large molecules facing steric hindrance and small molecules exhibiting weak retention. Bearing this in mind, we decided that we could not choose a single dye molecule that could serve as a general probe for a wide range of materials with different pore sizes and polarities. Instead, we selected an array of dye molecules with different sizes and chemical characteristics. Four criteria were used to guide the selection of these dye molecules (Figure 2): i) The dyes should exhibit distinct and stable absorbance in the visible region for analysis using a standard camera; ii) The dyes should all be water‐soluble to allow the use of a common carrier solvent, also biased by the fact that there are many applications, such as contaminant removal, where water is the relevant medium; iii) The dyes should cover a range of molecular weights and molecular sizes to accommodate materials with varying pore size distributions and overall porosities, potentially revealing additional information on pore size; and iv) avoidance of dyes that either fail to be adsorbed into porous materials or that are adsorbed excessively, on the basis that these dyes would be less discriminatory in the colorimetric assay.

Given these four principles, we designed a colorimetric array that contained six diverse dye molecules (Figure 2). Specifically, this array includes the following cationic and anionic molecules: Acridine orange, Safranin O, Crystal violet, Methyl orange, Ponceau xylidine, and Lucifer yellow, selected based on their chemical stability and photostability.^[^ ^42^, ^43^ ^]^ For example, Methyl orange, a low‐cost and commercially available dye with low toxicity, has been used before as an adsorption indicator for evaluating the adsorption capacity of adsorbents in wastewater.^[^ ^44^ ^]^ Ponceau xylidine^[^ ^45^ ^]^ has been used for microscopy staining to reveal tissue and cellular structure.

To better understand the size of these dye molecules, geometry optimization, and energy calculations were performed using the Forcite module (Figure S15). The narrowest dimensions of the six dye molecules ranged from 0.67 to 1.37 nm.

In an initial design, the array included two additional dye candidates, resazurin sodium and methylene blue, but these were removed due to their poor selectivity (Figures S16 and S17).

### Colorimetry Using Computer Vision Analysis

Following the design of the dye array, a colorimetry‐based computer vision method was developed as an in situ analysis tool (Figure S18). The dye array is imaged using a simple low‐cost web camera before and after adsorption by the materials (18 h time gap between measurements), with the outcomes captured as red, green, and blue (RGB) light channel intensities. To validate the feasibility of our approach, the RGB channel sensitivity of each dye was evaluated by imaging dye solutions (Figure S19). Each dye demonstrated a strong linear relationship (*R* ^2^ > 0.98) in either the red, green, or blue channel, corresponding to dye concentration changes ranging from 1 to 10 ppm (Figure S20, see Figures S22 and S23 for concentration data below 0.5 ppm). Moreover, a strong Pearson correlation (>0.99) was observed between the RGB channel intensity and the maximum absorbance obtained from UV–vis spectroscopic analysis (Figure S21). This high correlation is attributed to the significant overlap in visible light absorption at specific wavelengths, enabling each dye to be represented reliably by its respective R, G, or B channel intensity. Thus, the concentration of each dye after adsorption was quantified based on the relation between concentration and channel intensity. Due to the considerable number of samples, we created a Python script to automate image processing. The script defines the region of interest (ROI), extracts RGB values, calculates the corresponding concentration after adsorption, and classifies the output into three levels: “hit,” “moderate fade,” or “none”.

To verify the response of the multichannel colorimetric array against known materials, we conducted a preliminary test using four commercially available solids with known porosity as model materials. These materials were two porous activated carbons and two nonporous linear polymers, polycaprolactone, and a Tenax polymer based on 2,6‐diphenyl‐p‐phenylene oxide. The experiment followed a four‐step approach: sample preparation (Step 1), adsorption and equilibrium (Step 2), filtration (Step 3), and vision‐based analysis (Step 4). Due to their high porosity and hierarchical pore structure, the activated carbons (Figure 2 materials **A** and **B**) exhibited excellent adsorptions for all six dyes, with all dye solutions becoming colorless over the measurements. This served as a benchmark for highly porous materials with a broad pore size distribution. By contrast, nonporous linear polymers (Figure 2 materials **C** and **D**) displayed poor adsorption capacity for dyes, with no marked color change across the colorimetric array compared with stock dye solution, demonstrating that surface adsorption on nonporous particles is not an issue, at least for these polymers. These results suggested that our method was sound, at least for materials that were highly porous or completely nonporous.

### Assay Development

**Figure 3:**
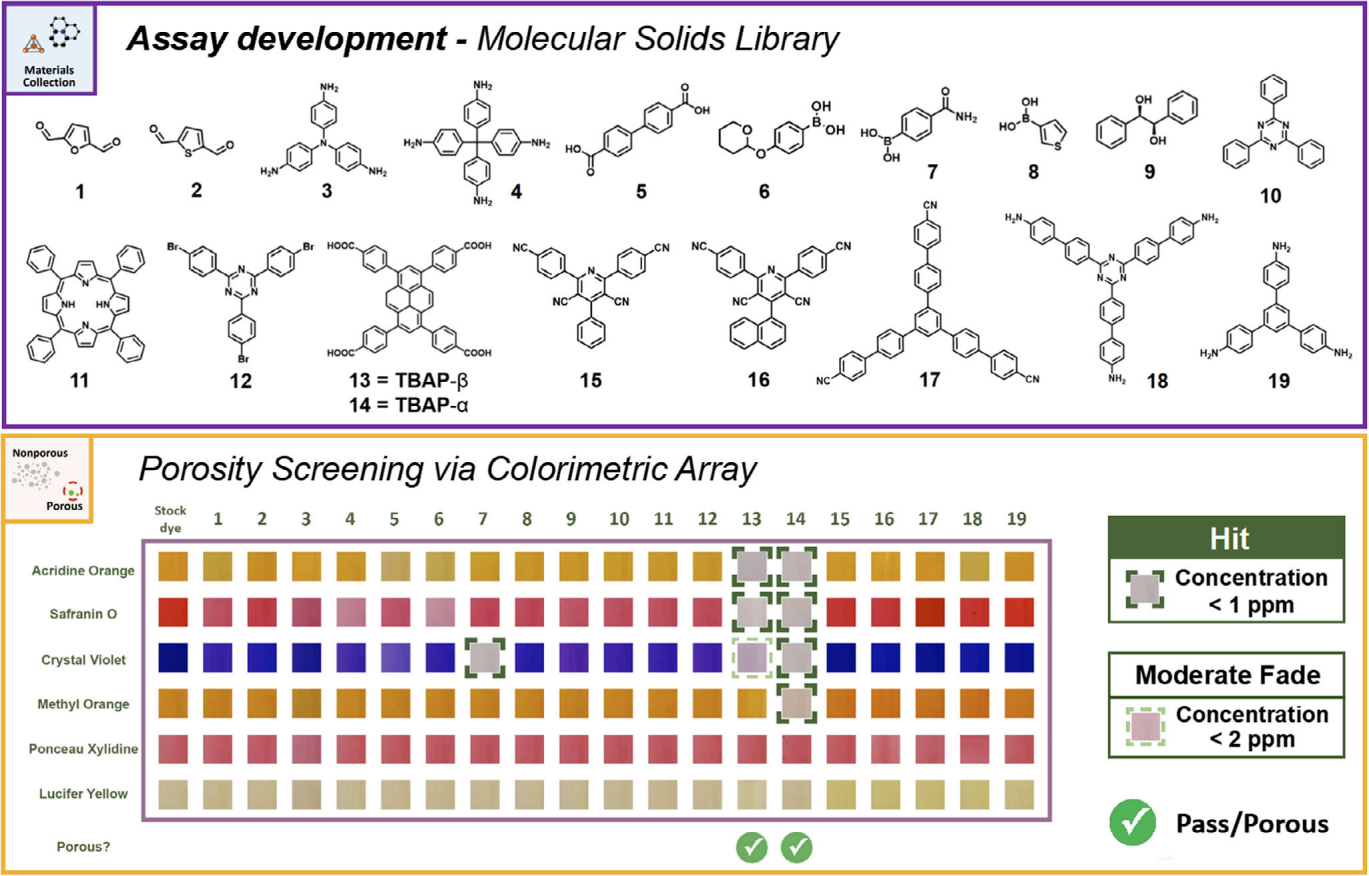
Assay development via porosity screening for a library of porous and nonporous molecular solids. (Top) Structures of nineteen molecular solids; (Bottom) Colorimetric map showing the images of nineteen molecular solids and stock dye solutions. (For display purposes, regions of interest (ROI) of each image are cropped from the images and combined to generate a color‐change profile. Original images can be found in Supporting Information.) A full color fade (concentration below 1 ppm) is classified as a “hit”, highlighted by a green dotted box. A moderate color fade while retaining some color (concentration between 1 and 2 ppm) is defined as a “Moderate fade,” marked with light green dotted lines, but is not currently included in the classification threshold.

Next, we evaluated our assay for the more challenging case of an array of molecular solids, some of which had much lower porosity levels and pore sizes than the activated carbons tackled above, as well as specific, well‐defined pore sizes that are defined by their crystalline structures. The materials selected are appended with a wide range of functional groups, such as aldehydes, amines, carboxylic acids, and cyanides, and the library includes a porphyrin‐based molecule (Figure 3). We used this approach to assess the chemical stability of the dye array (e.g., to probe the scope for chemisorption on the particle surface of nonporous materials). Given the strong tendency of organic crystals to adopt dense structures, porosity is uncommon. From the 19 molecular solids selected for this test, only **13**, 4,4′,4′“,4′”'‐(pyrene‐1,3,6,8‐tetrayl)tetrabenzoic acid (TBAP‐β ) (BET surface area of 275 m^2^ g^−1^ and total pore volume of 0.14 cm^3^ g^−1^) and **14**, TBAP‐α, (BET surface area of 2011 m^2^ g^−1^ and total pore volume of 0.83 cm^3^ g^−1^) had permanent porosity. These two materials, **13** and **14**, are the same molecule but exist as different polymorphs. As reported by Aitchison et al.,^[^ ^46^ ^]^ **14** forms a porous π‐stacked structure between the pyrene cores through crystallization, which significantly contributes to its overall porosity (PXRD shown in Figure S24). By contrast, the porosity in the polymorph TBAP is very modest, making this a challenging but representative case study for this class of materials.

Most molecular solids in the library produced a negligible color change (approaching “none”), with only samples **7**, **13,** and **14** showing varying degrees of color fading (from “moderate” to “full”) for 1, 3 and 4 of the six dyes, respectively. We used these data to define a “hit” as a sample that exhibits a full color fade (concentration below 1 ppm), highlighted by a dark green dotted box in Figure 3, or a “moderate fade” (concentration between 1 and 2 ppm) highlighted by a light green dotted box in Figure 3. **13** produced two “hits” and one “moderate fade”, whereas **14** produced four hits. The single “hit” from the nonporous molecular solids **7**, (4‐carbamoylphenyl)boronic acid, was most likely attributed to a redox reaction, where the boronic acid acted as a mild reducing agent, converting the colored cationic form of crystal violet into its colorless form in solution. This exemplifies the value of our multichannel method since **7** would have been a false positive in a single channel assay using Crystal violet.

To confirm that the crystal structures of **13** and **14** remained intact during the colorimetric array experiment—that is, that they are stable in water and retain their porous structures—PXRD analysis was performed before and after dye adsorption (Figures S25 and S26), which showed no apparent structural changes. Overall, these results demonstrated the stability and selectivity of the colorimetric array in detecting dye adsorption and its ability to distinguish between polymorphs with different porosity levels, suggesting that the multichannel method can be semi‐quantitative in some cases.

**Figure 4:**
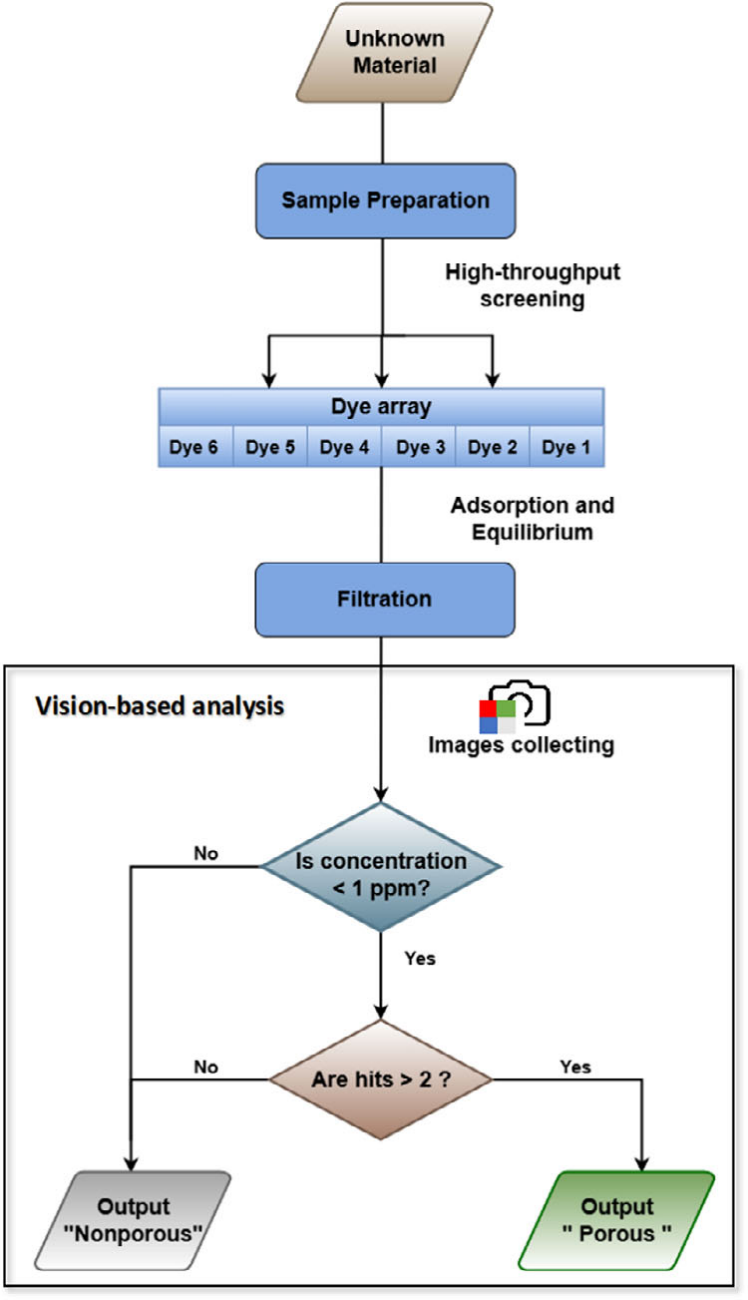
Flowchart illustrating the computer vision‐based analysis process for accelerating the discovery of porous materials using a multichannel colorimetric array.

Based on the average score observed in these molecular solids, we established a general threshold for the assay, that is, a material was classified as porous if it exhibits at least two “hits” (Figure 4). Conversely, if the sample fails to meet this threshold, it is categorized as nonporous. This threshold gives the correct outcome for modestly porous materials such as **13** but excludes false positives such as **7**.

To compare our method with conventional gas adsorption measurements, we established a corresponding reference benchmark based on total pore volume to distinguish between porous and nonporous materials. Classifying porosity using the BET surface area can sometimes be subjective as materials can exhibit complex adsorption isotherms that are problematic or ambiguous to fit with the BET model.^[^ ^47^, ^48^ ^]^ Here, we opted to use total pore volume instead as the benchmark since it is derived by directly converting the amount of gas adsorbed by a material using known relationships between gas volume and pore filling. A total pore volume of 0.10 cm^3^ g^−1^ (at *P/P_0 _* = 0.9) was set as the threshold limit for a porous material, although this could be adjusted; for example, in a workflow that focused on discovering highly porous solids, this threshold definition might be set much higher. A flowchart outlining the sample classification process is shown in Figure 4.

### High‐Throughput Porosity Screening for Diverse Porous Materials

We extended our screening beyond molecular solids to consider three representative classes of materials: POCs, CMPs, and MOFs. These materials have different levels of structural order, ranging from amorphous to crystalline architectures and exhibit diverse chemical compositions. They also encompass a wide range of surface areas, pore size distributions, and gas adsorption behaviors, making them ideal candidates for evaluating the generality and versatility of the proposed screening method.

**Figure 5:**
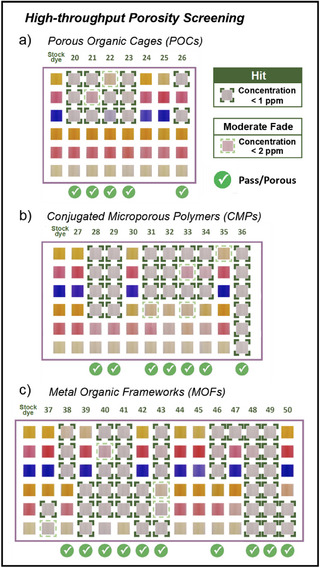
Colorimetric maps for a) organic cages, b) amorphous CMPs, and c) crystalline MOFs. A full color fade (concentration below 1 ppm) is classified as a “hit”, highlighted by a green dotted box. A dye concentration between 1 and 2 ppm is defined as a “Moderate fade,” marked with light green dotted lines (this is not currently included in the classification threshold (Figure 4)). For display purposes, regions of interest (ROI) of each image were cropped from the images and combined to generate the color‐change profile shown here. Original images can be found in Supporting Information. The target ROI channel intensity and corresponding concentration for each sample are provided in Table S6–S8.

POCs with prefabricated molecular pores are a class of molecular materials offering porosity in both the crystalline and the amorphous state. Here, we investigated five water‐stable imine cages^[^ ^49^ ^]^ and two corresponding dodecyl amine cages (Figure S27) using our dye array to assess their porosity. Five cage materials (**20**, **21**, **22**, **23**, and **26**) were classified correctly as porous according to our workflow definitions (Figure 4), showing hits from either two or three of the smallest dyes (Figure 5a). By contrast, the amine cages RCC1 (**24**) and RCC3 (**25**) exhibited zero response and were classified correctly as nonporous. Gas uptake measurements confirmed that all imine cages, CC1 (**20**), CC3 (**21**), CC4 (**22**), CC19 (**23**), and TFB‐CHEDA‐cage (**26**) possessed permanent porosity, with modest total pore volumes of 0.13, 0.19, 0.29, 0.14, and 0.23 cm^3^ g^−1^, respectively (see Table S12 for porosity information of all materials). RCC1 (**24**) and RCC3 (**25**) displayed pore volumes of <0.10 cm^3^ g^−1^, which is attributed to the flexible amine linkages that compromise shape persistence in these cages.

CMPs are constructed via the assembly of rigid building blocks through irreversible covalent bonds, and these materials are amorphous. We evaluated a series of CMPs that were synthesized under different reaction conditions and with different building blocks (Figure S27). Five CMPs comprised building blocks related to CMP‐1^[^ ^50^ ^]^ and were produced by coupling 1,3,5‐triethynylbenzene with different aryl halides. The other five CMPs were synthesized using a novel approach, employing 1,4‐diisocyanatobenzene in combination with compounds containing alkynyl substituents. Using the dye array, seven CMP samples (**28**, **29**, **31**, **32**, **33**, **34**, and **36**) exhibited between two and four “hits”, leading to their classification as porous (Figure 5b). Three CMP samples (**27**, **30**, and **35**) exhibited no hits in the dye array and were classified as nonporous. Subsequent gas uptake measurements confirmed that seven of the CMPs were “porous” and had total pore volumes ranging from 0.29 to 0.79 cm^3^ g^−1^, while the three “nonporous” samples all had pore volumes <0.10 cm^3^ g^−1^. Again, the colorimetric array classified CMP porosity successfully, yielding results that were consistent with gas uptake measurements.

MOFs are crystalline materials composed of metal nodes linked by organic ligands. Their rigid node‐linker topology and coordination bonds provide precise control over both structure and composition, enabling the modular design of function.^[^ ^51^ ^]^ We selected 14 MOFs comprising different metal cores and diverse organic linkers (Table S3). These MOFs were reported to exhibit different pore architectures, including microporous materials (ZIF‐8 and MOF‐808), mesoporous structures (UiO‐67 and NU‐1000), as well as flexible MOFs (MIL‐53 and MIL‐68) that exhibit dynamic changes in pore structure under different conditions.^[^ ^52^ ^]^ Using the dye array, ten MOFs (**38**–**43**, **46**, **48,** and **49**) were identified as porous (Figure 5c), which aligns with their conventional gas adsorption measurements. Among these, different MOFs showed diverse adsorption preferences. For example, MIL‐53‐Al‐BPDC (**39**), MIL‐101‐Cr (**42**), and MOF‐808 (**50**) adsorb large dye molecules in preference to smaller ones (Figure 5c). UiO‐66 (**48**) and NU‐1000‐csq (**49**), both featuring mesopores, showed the strongest response (6/6 hits) across the array. Further, gas uptake measurements confirmed that these two MOFs possessed high porosity levels, with pore volumes exceeding 0.90 cm^3^ g^−1^ and BET surface areas of 1802 and 1944 m^2^ g^−1^, respectively, again suggesting some semiquantitative behavior in this assay. Conversely, four samples were classified as nonporous. Samples **44**, **45**, and **47** exhibited zero or one hit in the dye array, which was largely consistent with subsequent gas uptake measurements, showing pore volumes below 0.10 cm^3^ g^−1^. The single exception was MOF‐801 (**37**), which yielded a false negative. MOF‐801 (**37**) was identified as porous by gas adsorption with a total pore volume of 0.39 cm^3^ g^−1^, but it only produced one “hit” and one “moderate fade” in the dye array, falling just below our threshold for being deemed porous. MOF‐801 is widely used for water harvesting due to its abundance of hydrophilic groups (─COOH and ─OH),^[^ ^53^ ^]^ and has a high water uptake (24 wt% at *P/P_0_* = 0.1 and 36 wt% at *P/P_0_* = 0.9). We hypothesize that the low dye response may be from competitive adsorption, where accessible pores are quickly occupied by water molecules due to hydrogen bonding with hydrophilic groups in the MOF pores, preventing larger dye molecules from accessing these sites. To eliminate the influence of water, we explored the adsorption behavior of MOFs in organic solvents. A solvent mixture of *n*‐heptane with the lowest polarity index and diethyl ether with good solubility for dyes (98/2%) was employed to simulate a nonpolar, hydrophobic environment (Table S4 and S5). Following the dye selection criteria outlined in Figure 1a, three dye molecules with varying molecular weights that demonstrate good solubility in the mixed solvents were selected (see Supporting Information for customized dye array section). However, by using this customized array, MOF‐801 did not yield any hits among the hydrophobic environments (Figure S28). This false negative could be corrected by incorporating “moderate fade” results in the decision algorithm (Figure 4).

We note that false negatives caused by strong water absorption were not observed universally. For example, MOF‐808 (**50**), also a hydrophilic material with high water uptake (12 wt% at *P/P_0_* = 0.1, 59 wt% at *P/P_0_* = 0.9),^[^ ^53^ ^]^ showed a low response (two hits) but was correctly classified as porous. This could be due to its larger pore diameters (8.8 and 18.4 Å) compared to MOF‐801 (7.4, 5.6, and 4.8 Å), allowing the pores to remain accessible to dye molecules. Moreover, the TFB‐CHEDA cage (**26**) shows good water adsorption (26 wt% at *P/P_0_* = 0.9) (Figure S29) but was classified correctly as porous with two hits, albeit a lower number of hits than the other four porous imine cages (Figure 5a). We have also tested three microporous zeolites. These results confirm that while specific highly hydrophilic zeolites (e.g., 13X) may present challenges for the dye adsorption method due to strong water adsorption, other zeolites (e.g., ZSM‐5, H‐USY) can still be effectively classified using this method (Figure S36).

Overall, the multichannel colorimetric array effectively identified and classified the porosity of the MOF materials in this diverse library, with results closely aligning with gas uptake measurements. While applying a single unified benchmark to such a diverse group of materials is challenging, our threshold of two hits successfully categorized all but one of the MOF samples, including those with breathing capabilities or larger pore diameters. Only MOF‐801 (**37**) was misclassified, possibly due to competitive water adsorption, though we note that for some applications (e.g., adsorption of pollutants from water), this might in fact be the correct functional classification.

### Overview of Method Performance

**Figure 6:**
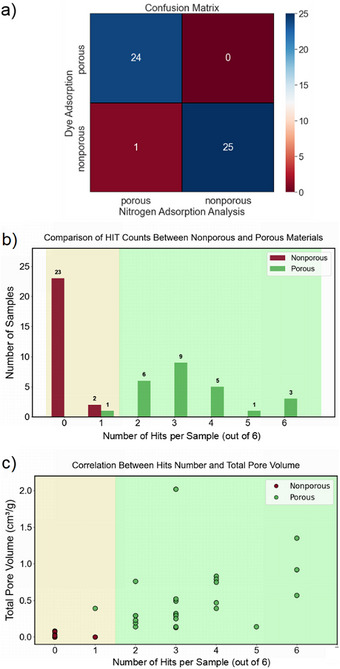
Comparison of dye adsorption screening against conventional gas adsorption measurements. a) Confusion matrix of 50 materials comparing the results of the colorimetric array and nitrogen uptake measurements. b) Distribution of the number of “hits” per sample from the six screened dyes for the nonporous and porous samples studied. c) Correlation between the number of “hits” per sample from the six screened dyes and total pore volume.

Overall, a total of 24 materials were correctly identified by this assay as porous, while 25 materials were classified correctly as nonporous. As such, the assay achieved a 98% accuracy (Figure 6a) in distinguishing porous from nonporous samples based on our definitions, which aligns with gas uptakes, at least for the range of porous solids exemplified here (see Supporting Information Section 5 for gas adsorption summary). The reproducibility test, presented in Figure S30, further demonstrates the robustness of the multichannel dye adsorption approach, and minor variations in the individual channels do not impact on the overall classification accuracy. Moreover, Figure 6b shows that nonporous samples exhibit a significantly higher frequency at zero hits, indicating that most remain largely unaffected. Samples with fewer than two hits have a total pore volume below 0.10 cm^3^ g^−1^ (Figure 6c). The total pore volume of the porous samples ranges from 0.13 to 2.02 cm^3^ g^−1^, while the BET surface area ranges from 67 to 2955 cm^3^ g^−1^.

To further investigate the impact of threshold adjustments, we tested other thresholds. We investigated two alternative classification thresholds: i) Lowering the threshold of a “hit” resulted in two false positives and a classification accuracy of 96% (Figure S31a). ii) Reclassifying a “moderate fade” as a “hit” improved the classification accuracy to 100%, correctly identifying one additional porous MOF, MOF‐801 (Figure S32). A common feature of these thresholds is the elimination of false negatives, ensuring that no porous materials are missed. Given the pre‐screening nature of our method, minimizing false negatives is important to prevent the exclusion of potentially valuable porous materials. By contrast, false positives can be checked by conventional gas adsorption methods though we note that no false positives occurred in these tests according to the thresholds used.

The design of this multichannel colorimetric array is also flexible and can be customized to deal with various material systems. For example, we showed that for the molecular solids, organic cages, and conjugated microporous polymers (CMPs), it was possible to use a reduced subarray consisting of just the three smallest dyes to classify the porosity (Crystal violet, Safranin O, and Acridine orange), while still achieving accurate classification without any false outcomes (Figure S33), thus increasing the speed and lowering the footprint of the assay. This adaptability allows researchers to tailor the array to the specific requirements of their particular study.

### Comparison of Colorimetric Analysis Timescales with Conventional Gas Adsorption Measurements

**Figure 7:**
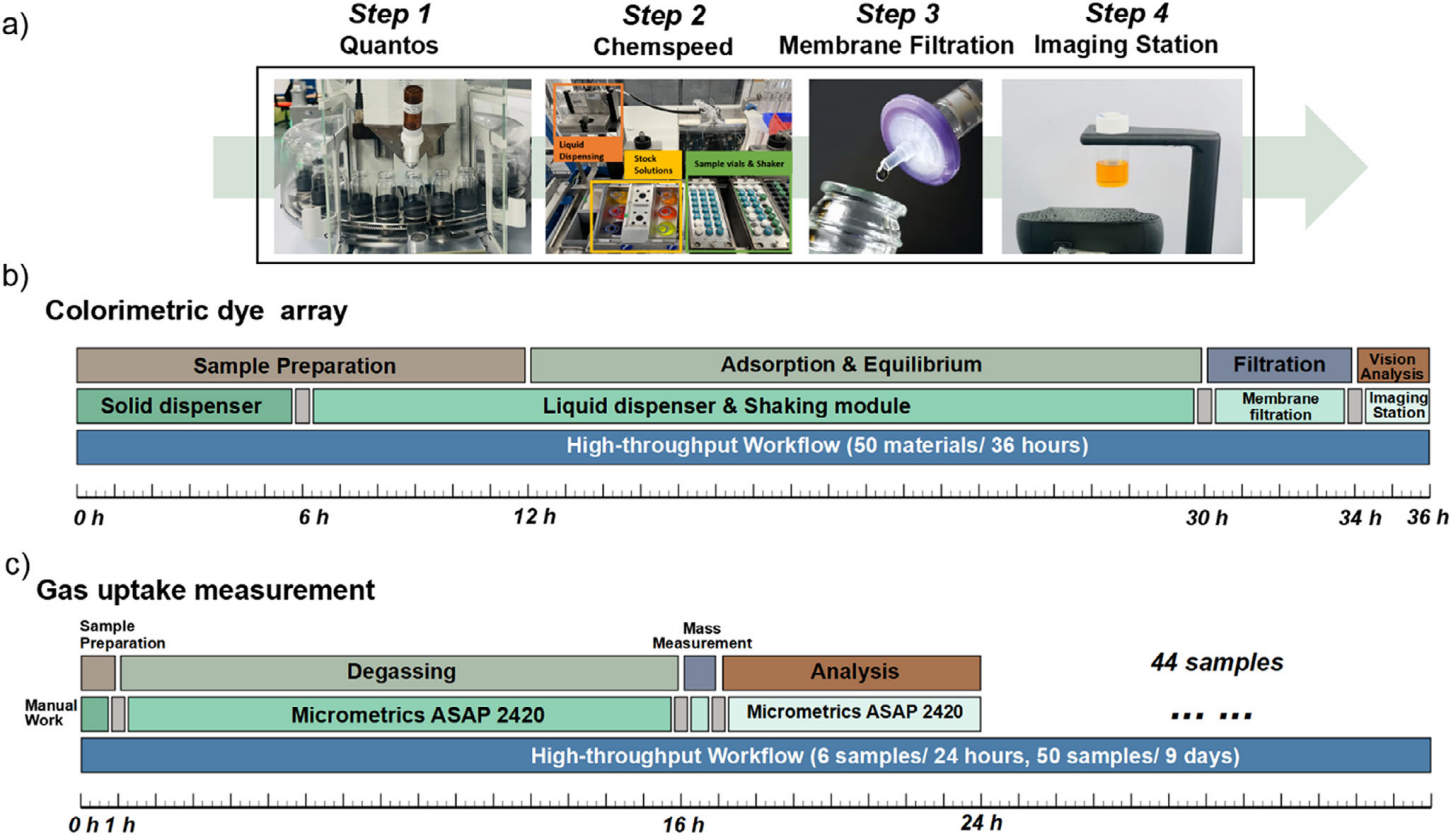
Workflows and timelines for the semiautomated dye adsorption method and a gas adsorption measurement using a six‐port Micromeritics ASAP 2420 parallel sorption analyzer. a) Semiautomated dye adsorption workflow, featuring four stations showing the layout of the robotic platforms used for the process. b) Timeline for screening 50 materials using the colorimetric dye array (36 h, average 43 min per sample). c) Timeline for screening 50 materials using gas uptake measurements by using the six‐port Micromeritics ASAP 2420 analyzer (9 days, average 259 min per sample). Note that the timescale for a more traditional single port sorption analyzer would be about six times as long to analyze 50 samples (i.e., approx. 50 days, or 1 day per sample).

Figure 7a gives an overview of the high‐throughput semiautomated workflow, illustrating the four stations and the layout of the robotic platforms. The procedure begins with automated powder dispensing (using a Mettler–Toledo Quantos), followed by liquid dispensing (on a Chemspeed platform, Figure S34), shaking, membrane filtration, and image acquisition. As shown in Figure 7b, porosity screening of above 50 materials using this workflow was completed in approximately 36 h (including 18 h for the dye adsorption process), which is much faster than traditional gas uptake methods that require around 9 days for 50 materials when using an instrument (Micromeritics 2420) with six parallel analysis ports (Figure 7c). We used this 6‐port instrument for convenience, but we note that most laboratories use sorption analyzers with a single analysis port, which would make the timescale 6 times longer than quoted in Figure 7c. As such, our colorimetric method is around 30 times faster than conventional porosity analysis. Coupled with its 98–100% accuracy for our sample library, makes our approach a viable pre‐screening method for candidate libraries of porous materials.

Beyond speed, this colorimetric array method offers three other advantages. First, it is scalable to larger batch sizes. For example, it could readily process more than 50 samples simultaneously with some technical modifications, making the method even faster. Second, this method requires very little material; for each material tested with 6 dyes, only 36 mg of sample was needed (6 mg per dye), and this would scale to just 18 mg with the reduced three‐channel method discussed above. This is substantially lower than the requirements for gas sorption measurements, with a general sample recommendation of >100 mg, depending on the porosity level. Third, while we present here a semiautomated platform supported by modular robotics, this method can also be implemented using existing, less costly, and accessible equipment, such as mechanical manual pipettes and magnetic plate stirrers. Compared to costly gas adsorption instruments, the dye array method offers an affordable alternative for initial porosity evaluation, making it accessible to resource‐limited labs; in principle, the minimal purchase required to set up this method is 3–6 inexpensive commercial dyes and a standard webcam.

### Limitations

Our screening method also has certain limitations, in addition to the false negative caused by strong water adsorption discussed above. First, false positives could result from material swelling, for example, in flexible materials such as certain polymers that can absorb water and expand in volume, thus facilitating dye absorption. Of course, this is a form of absorption, but it does not correlate with dry‐state porosity, if that is the topic of interest. Secondly, while most (if not all) porous materials used in industry applications are water stable, some synthetic materials will lose porosity if they are moisture sensitive or immersed in water for a prolonged period. For example, although HKUST‐1 has good porosity (BET surface area of 892 m^2^ g^−1^), it only showed one positive hit in our dye array due to its limited water stability^[^ ^54^ ^]^ (Figure S35). In fact, we view this as a potential advantage given the relatively niche application scope for porous materials that are highly water sensitive. A minor limitation is the difficulty of sample recycling in this assay, although this is mitigated by the small amount of sample that is required.

## Conclusions

With the advent of high‐throughput robotic synthesis methods, property measurements can become a new bottleneck. This is true for porous materials, where the rate of synthesis can easily outstrip the rate of analysis, even in laboratories that do not use automated synthesis methods. To address this, we developed a high‐throughput screening method that combines a colorimetric array and computer vision analysis for rapid porosity classification. This method successfully screened organic cages, CMPs, and MOFs with a wide range of porosity levels, achieving a classification accuracy of 98–100% in alignment with conventional gas uptake measurements. The inherent flexibility of the threshold system ensures adaptability, allowing researchers to customize the dye array and the thresholds according to their specific screening objectives.

Porous materials are inherently modular and can be tailored through the careful selection of organic building blocks and architectures.^[^ ^55^ ^]^ This is “molecular Lego” approach enables the design of many related classes of materials with similar characteristics. For instance, metal–organic cages are another growing class of porous solids that we did not investigate here, and like Zr‐MOFs, a series of MOCs assembled from zirconium oxide clusters were reported to exhibit high water stability.^[^ ^56^ ^]^ Moreover, covalent organic framework (COF) is another large and diverse class of porous materials that could be investigated using this method.

## Conflict of Interests

The authors declare no conflict of interest.

## Supporting information



Supporting Information

## Data Availability

The code for computer vision analysis is available at https://doi.org/10.5281/zenodo.15364478. The corresponding repository and imaged data used for this work can be accessed at https://github.com/yushuhankungfuGigi/Colorimetric_Dye_Array.
