# Supplementary material for: Accelerated Porosity Screening Using a Multichannel Colorimetric Array
Source: Angew Chem Int Ed Engl. 2025 Aug 21;64(40):e202510400. doi: 10.1002/anie.202510400 (PMC12462744; doi:10.1002/anie.202510400)
Supplement: Supplementary file 1 — Supporting Information [file ANIE-64-e202510400-s001.docx]

**Supporting Information**

**Accelerated Porosity Screening using a Multichannel Colorimetric Array**

Yushu Han,^[a]^ Isaiah Borne,^[a]^ Biplab Dutta,^[a]^ Rob Clowes,^[a]^ Hang Qu,^[a]^ Alex James,^[a]^ Charlotte E. Boott,*^[a]^ Marc A. Little, *^[a, b]^ and Andrew I. Cooper *^[a]^

[a] Department of Chemistry and Materials Innovation Factory
University of Liverpool
51 Oxford Street, Liverpool L7 3NY, UK
E-mail: c.boott@liverpool.ac.uk, aicooper@liverpool.ac.uk

[b] Institute of Chemical Sciences
Heriot-Watt University
EH14 4AS, Edinburgh, UK

E-mail: m.little@hw.ac.uk

Table of Contents

Section 1.[Experimental Materials 2](#_Toc201267941)

Section 2.[Experimental methods 13](#_Toc201267942)

Section 3.[The Design of Dye Array 16](#_Toc201267943)

Section 4.[Experimental results 24](#_Toc201267944)

Section 5.[Gas Adsorption Summary 47](#_Toc201267945)

Section 6.[References 67](#_Toc201267946)

This PDF file includes:

Figures S1 to S73

Tables S1 to S12

# **Experimental Materials**

Activated carbon **A (**DARCO**^®^)**, Activated carbon **B (**NORIT^®^ A SUPRA EUR USP**),** polycaprolactone **C** and Tenax® Polymer Adsorbent **D** and were obtained from Sigma-Aldrich.

TBAP (**13** and **14**) was prepared and crystallized according to previous methods.^1^ CC1, CC3, CC4, CC19, TFB-CHEDA-Cage, RCC1 and RCC3 (**20** – **26**) were prepared and crystallized according to previous methods.^2–6^ MOF 801, UiO-67, NU-1000, MOF808, Ni-BTC, MIL-125, Ni-PYMS, (**37, 45** – **50**) were prepared and crystallized according to previous methods.^7–13^ Other MOFs and three zeolites were purchased from Sigma-Aldrich and Beijing Huawei Ruike Chemical Co., Ltd., and Zeolyst international, and activated before using. General synthesis procedure for DAT-Series Molecules (**15** and **16**) and porous polymers (**27** – **36**) is provided below. All other tested materials not explicitly mentioned were obtained from Sigma-Aldrich, TCI Europe, Fisher, BLDpharm and ET Co., Ltd., and used as received. All sample sources and details are provided in Tables S1 below. All materials were stored under ambient conditions and activated prior to gas adsorption measurements. All dye molecules, Acridine Orange (dye 6), Safranin O (dye 5), Crystal Violet (dye 4), Methyl Orange (dye 3), Ponceau Xylidine (dye 2), and Lucifer Yellow (dye 1), were obtained from Sigma-Aldrich and Alfa Aesar. Anhydrous solvents were purchased from Acros Organics and used without further purification. All gases for sorption analysis were supplied by BOC at a purity of ≥ 99.999%.


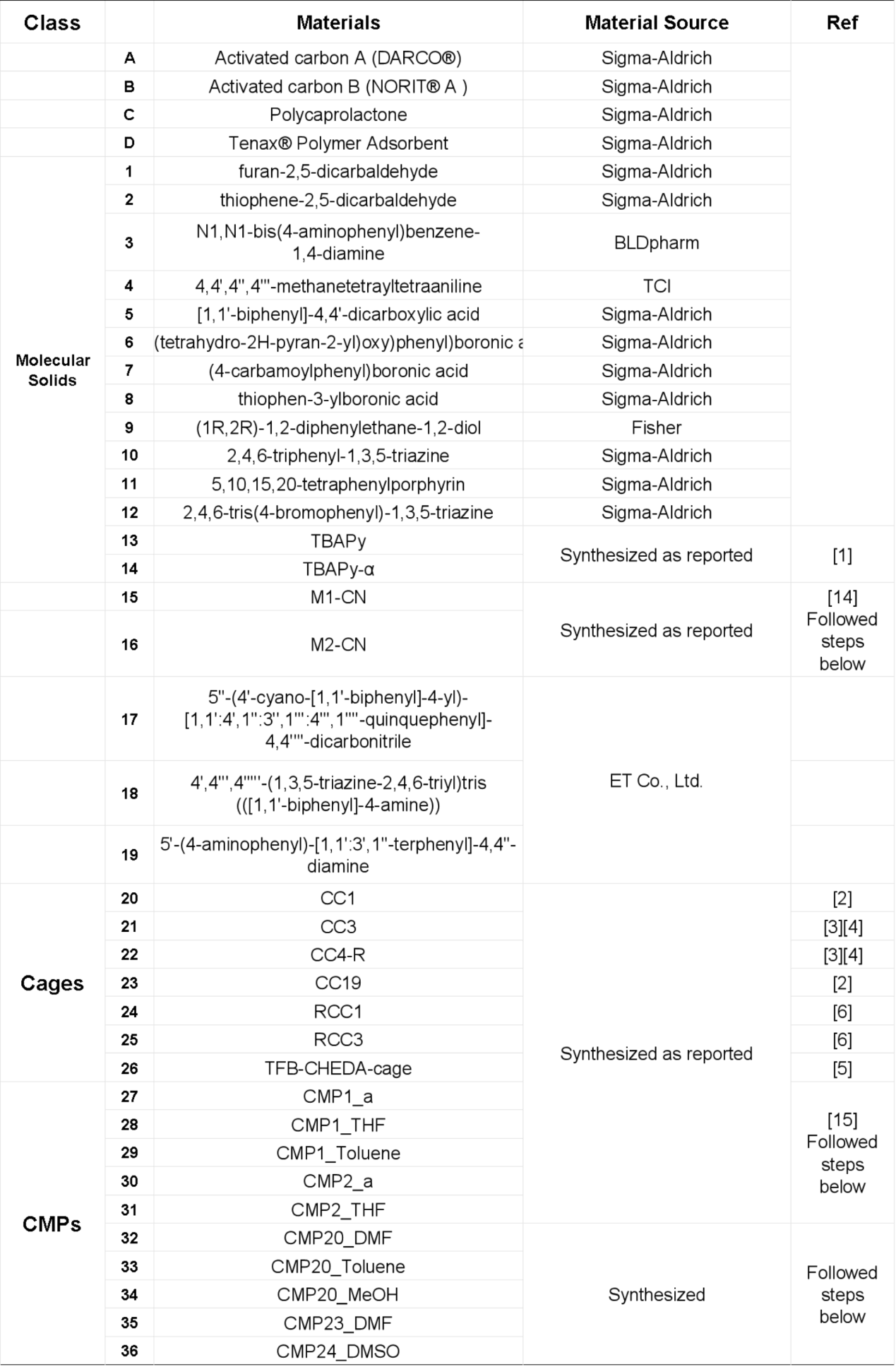


**Table S1 The sources and details of 58 tested materials**


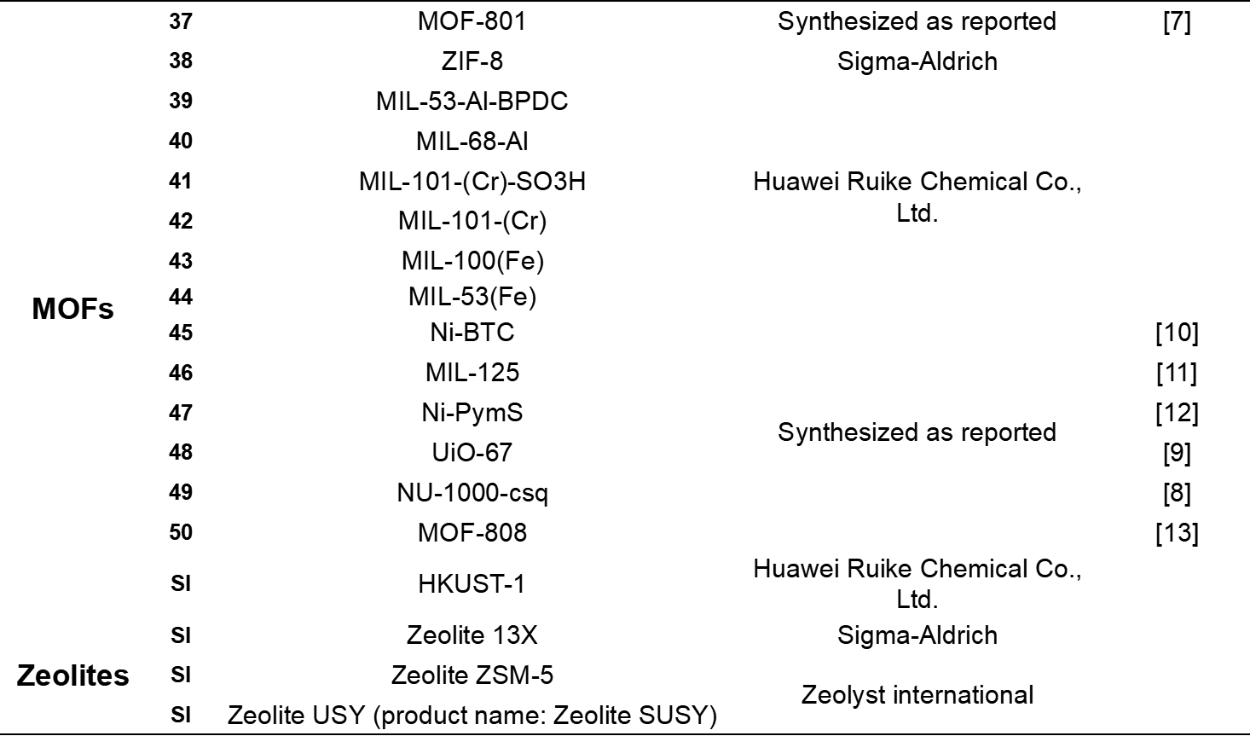


*Synthesis of* ***15 (M1-CN)***

According to previous method,^14^ 4-(2-cyanoacetyl)benzonitrile (368 mg), benzaldehyde (106 mg), and ammonium acetate (540 mg) were discharged into the reaction flask, then 14 ml acetic acid was added. The reaction mixture was heated to 110 ^o^C overnight. The resultant precipitates were filtered and washed with methanol. Then, the solids were oxidized with 2,3-dichloro-5,6-d icyano-p-benzoquinone (530 mg) in acetic acid solution (30 ml) under 110 ^o^C for 1 h, followed by filtration and methanol washing. The solids were purified by column chromatography, giving the final product as an orange powder (326 mg, 80%).

^1^H NMR (400 MHz, DMSO-*d6*) δ 8.24 (m, 4H), 8.14 (m, 4H), 7.83 (m, 2H), 7.69 (m, 3H).

*Synthesis of* ***16 (M2-CN)***

According to previously reported method^14^, 4-(2-Cyanoacetyl)benzonitrile (90 mg, 1-naphthaldehyde (37.2 mg) and ammonium acetate (95.2 mg) in 5 mL acetic acid and then 135 mg DDQ were used to generate the final product as solid (94.7 mg, 87%).

^1^H NMR (400 MHz, CDCl_3_) δ 8.24 (dt, *J*_HH_ = 8.7 Hz, 1.64, 4H), 8.14 (d, *J*_HH_ = 8.3 Hz, 1H), 8.04 (d, *J*_HH_ = 7.5 Hz, 1H), 7.89 (dt, *J*_HH_ = 5.1 Hz, 1.96, 4H), 7.61 (m, 3H), 7.41 (d, *J*_HH_ = 8.4 Hz, 1H).

*Synthesis of* **27**, **28**, and **29 (CMP-1)**

Typical procedure for CMP-1 followed previous method^15^: 1,3,5-triethynylbenzene (300 mg), 1,4-diiodobenzene (659 mg), tetrakis-(triphenylphosphine)palladium (100 mg), and copper iodide (30 mg) were dissolved in a mixture of solvents. The reaction mixture was heated to 80 ℃, stirred for 24 h under a nitrogen atmosphere, and then cooled to room temperature. The precipitated network polymer was filtered and washed four times with chloroform, water, methanol, and acetone to remove any unreacted monomer or catalyst residues. The further purification of the polymers was carried out by Soxhlet extraction from methanol for 48 h. The product was dried in vacuum for 24 h at 70 °C.

**27**: This network was synthesized in toluene (3 mL) and Et_3_N (3 mL) and had been stored under air for over ten years. IR (cm^-1^): no peak observed from (−C≡C−H), 2200 (−C≡C−)

**28**: This network was freshly synthesized in tetrahydrofuran (THF, 6 mL). IR (cm^-1^): 3036 (−C≡C−H), 2208 (−C≡C−)

**29**: This network was freshly synthesized in toluene (6 mL). IR (cm^-1^): no peak observed from (−C≡C−H), 2201 (−C≡C−)

*Synthesis of* **30 and 31 (CMP-2)**

Typical procedure for CMP-2 followed previous method^15^: 1,3,5-triethynylbenzene (300 mg), 4,4′-diiododiphenyl (812 mg), tetrakis-(triphenylphosphine)palladium (120 mg), and copper iodide (30 mg) were dissolved in a mixture of solvents. The reaction mixture was heated to 80 ℃, stirred for 24 h under a nitrogen atmosphere, and then cooled to room temperature. The precipitated network polymer was filtered and washed four times with chloroform, water, methanol, and acetone to remove any unreacted monomer or catalyst residues. The further purification of the polymers was carried out by Soxhlet extraction from methanol for 48 h. The product was dried in vacuum for 24 h at 70 °C.

**30**: This network was synthesized in a mixture of toluene (3 mL) and Et_3_N (3 mL) and had been stored under air for over ten years. IR (cm^-1^): 3297 (−C≡C−H), 2206 (−C≡C−).

**31**: This network was freshly synthesized in THF (6 mL). IR (cm^-1^): no peak observed from (−C≡C−H), 2204 (−C≡C−).

*Synthesis of* **32, 33 and 34 (CMP-20)**

1,3,5-triethynylbenzene (150 mg, 1.0 mmol), 1,4-diisocyanatobenzene (160 mg), copper iodide (190 mg) and Et_3_N (100 mg) were dissolved in a mixture of solvents. The reaction mixture was heated to 100 ℃, stirred for 48 h under a nitrogen atmosphere, and then cooled to room temperature. The precipitated network polymer was filtered and washed four times with chloroform, water, methanol, and acetone to remove any unreacted monomer or catalyst residues. The further purification of the polymers was carried out by Soxhlet extraction from methanol for 48 h. The product was dried in vacuum for 24 h at 70 °C.

**32**: This network was synthesized in Dimethylformamide (DMF, 4 mL). IR (cm^-1^): 2846 (−C≡C−H), 2152 (−C≡C−).

**33**: This network was freshly synthesized in Toluene (4 mL). IR (cm^-1^): no peak observed from (−C≡C−H), 2207 (−C≡C−).

**34**:This network was freshly synthesized in Methanol (MeOH, 4 mL). IR (cm^-1^): no peak observed from (−C≡C−H), 2187 (−C≡C−).

*Synthesis of* **35 (CMP-23)**

Tri(prop-2-yn-1-yl)amine (131 mg), 1,4-diisocyanatobenzene (160 mg), copper iodide (190 mg) and potassium tert-butoxide (120 mg )were dissolved in a mixture of solvents. The reaction mixture was heated to 100 ℃, stirred for 48 h under a nitrogen atmosphere, and then cooled to room temperature. The precipitated network polymer was filtered and washed four times with chloroform, water, methanol, and acetone to remove any unreacted monomer or catalyst residues. The further purification of the polymers was carried out by Soxhlet extraction from methanol for 48 h. The product was dried in vacuum for 24 h at 70 °C.

**35**: This network was synthesized in DMF (4 mL). IR (cm^-1^): no peak observed from (−C≡C−H), 2201 (−C≡C−). 1205 (−C−N−)

*Synthesis of* **36 (CMP-24)**

Tetrakis(4-ethynylphenyl)methane (31.2 mg), 1,4-diisocyanatobenzene(16 mg), copper iodide (19 mg) and potassium tert-butoxide (12 mg) were dissolved in DMSO (0.8 mL). The reaction mixture was heated to 100 ℃, stirred for 48 h under a nitrogen atmosphere, and then cooled to room temperature. The precipitated network polymer was filtered and washed four times with chloroform, water, methanol, and acetone to remove any unreacted monomer or catalyst residues. The further purification of the polymers was carried out by Soxhlet extraction from methanol for 48 h. The product was dried in vacuum for 24 h at 70 °C. IR (cm^-1^): 3039 (−C≡C−H), 2152 (−C≡C−).


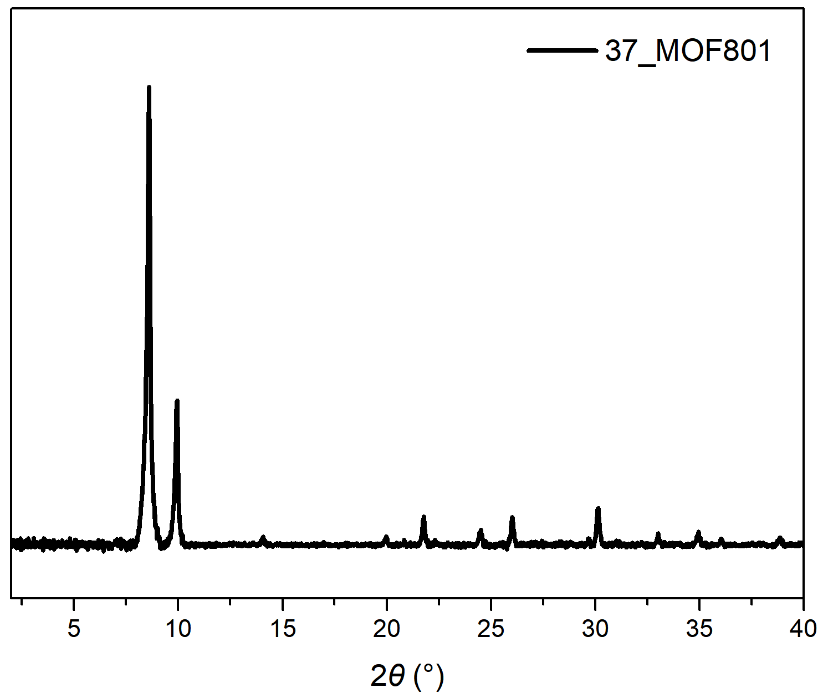


**Figure S1**. PXRD patterns for **37**


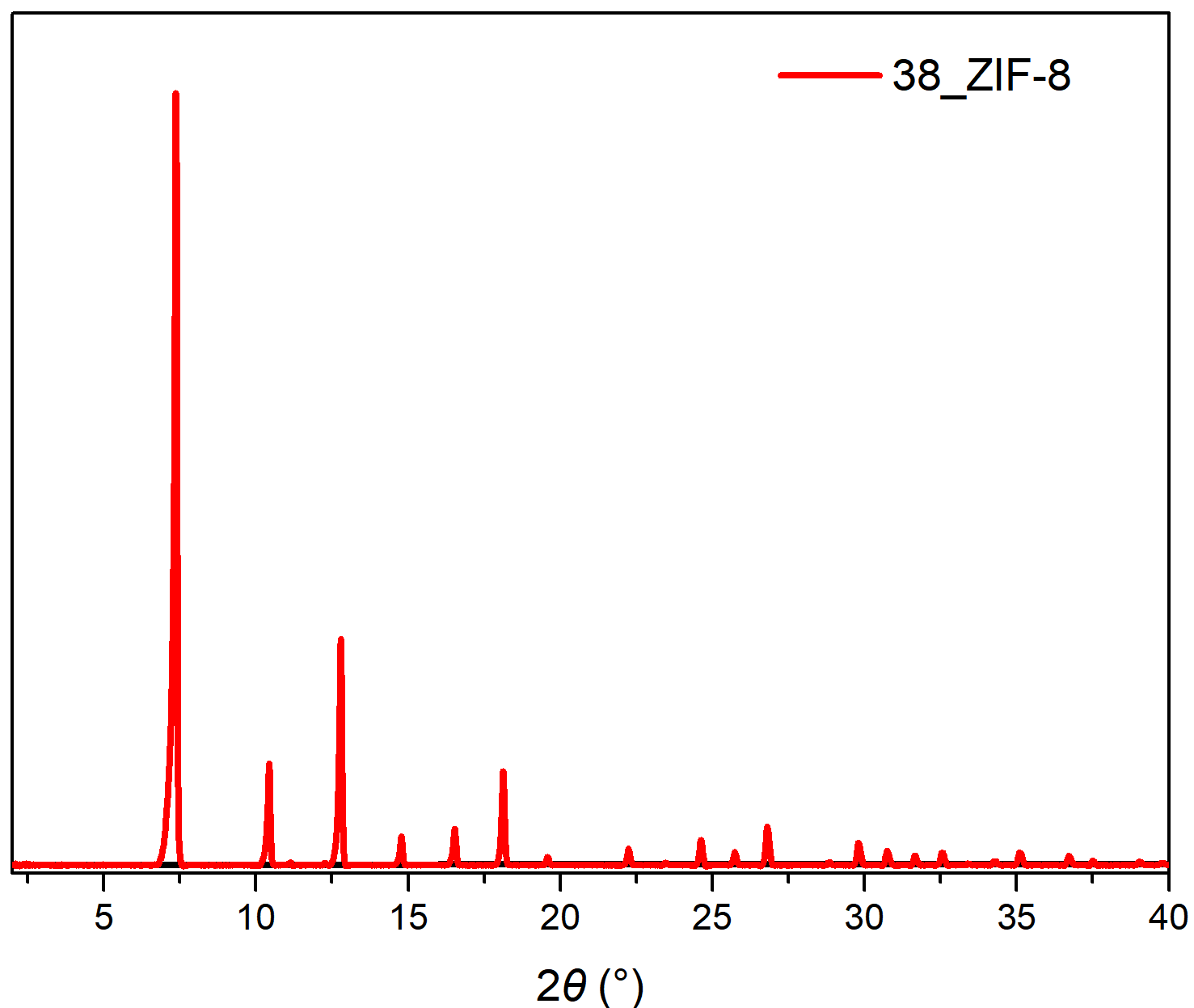


**Figure S2**. PXRD patterns for **38**

**
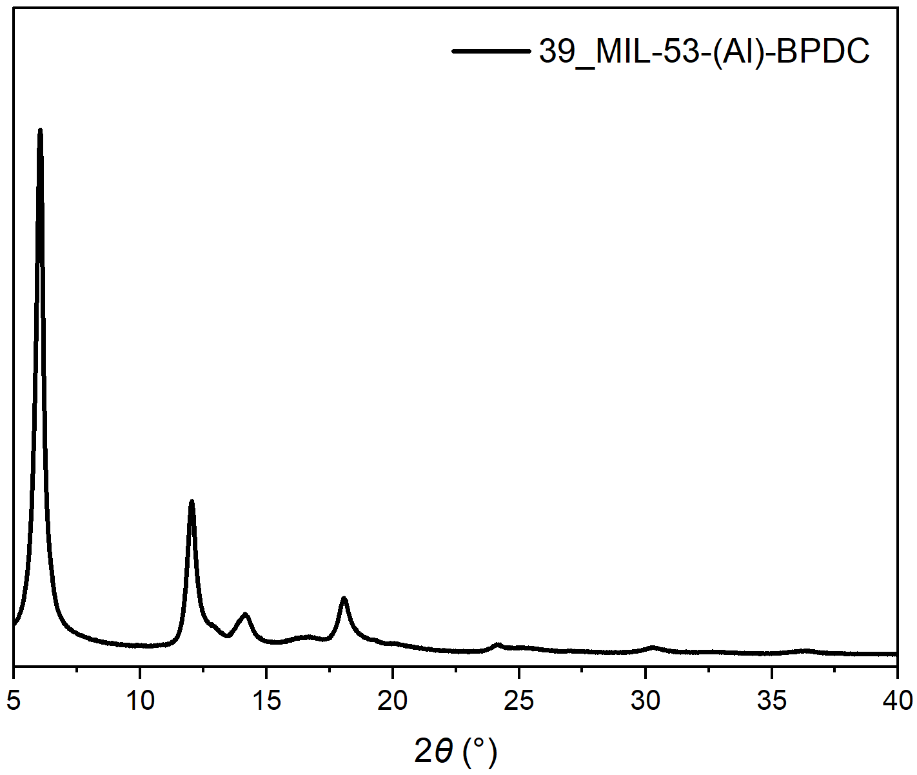
**

**Figure S3**. PXRD patterns for **39**

**
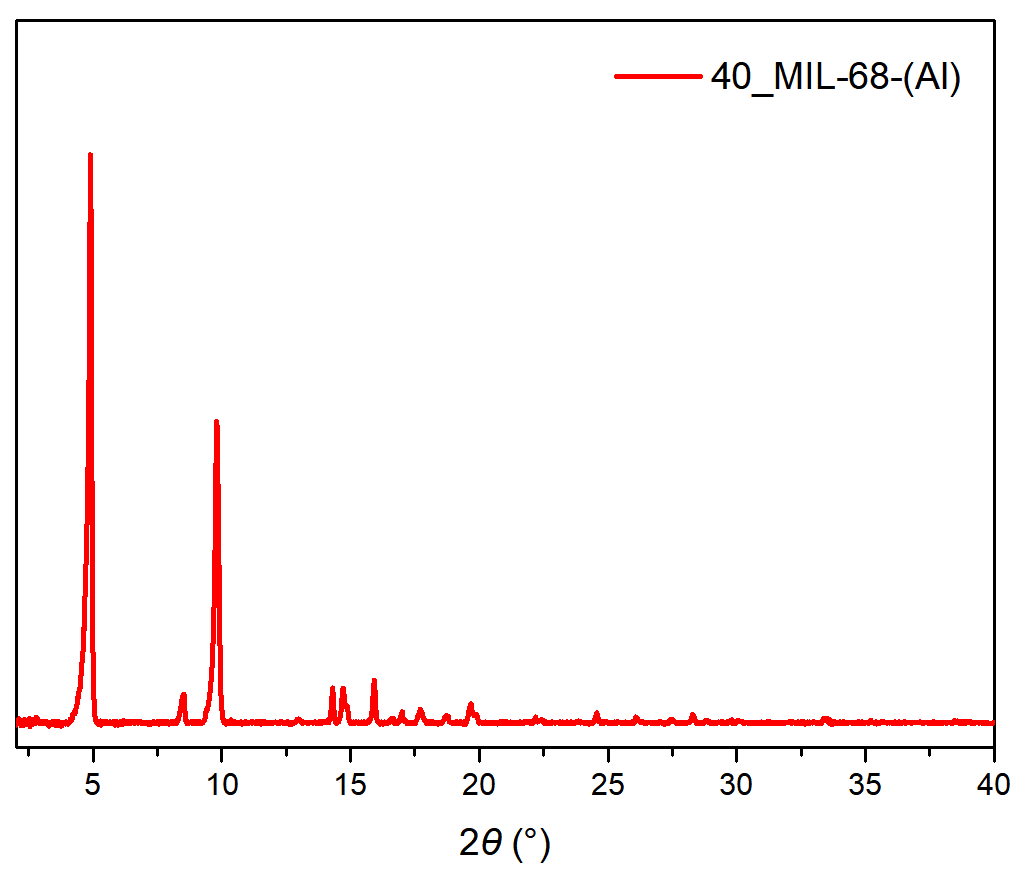
**

**Figure S4**. PXRD patterns for **40**

**
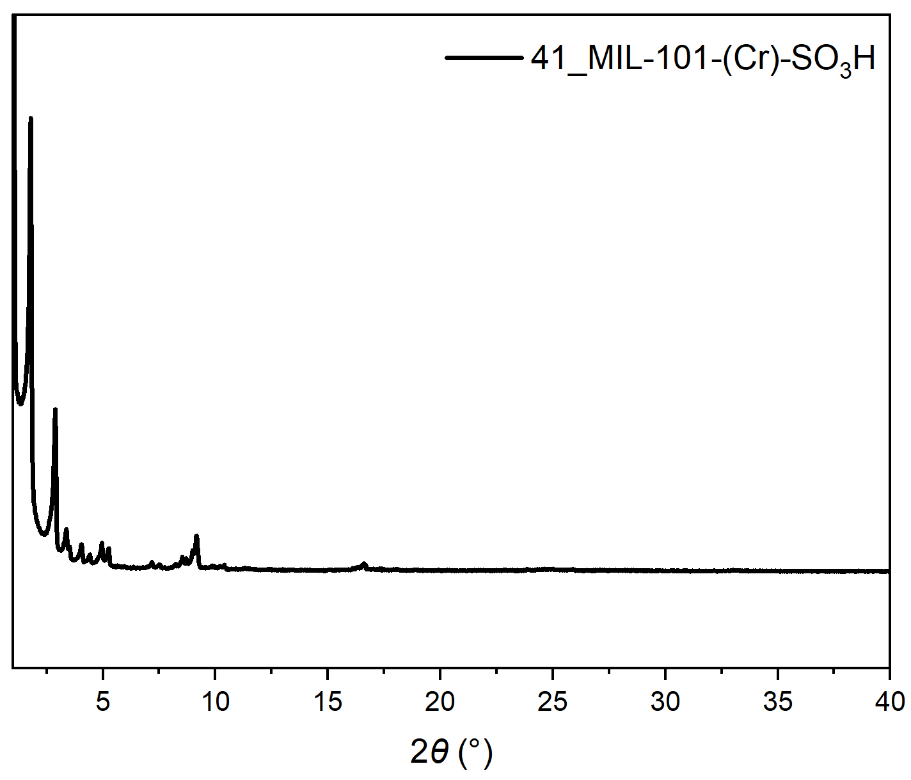
**

**Figure S5**. PXRD patterns for **41**

**
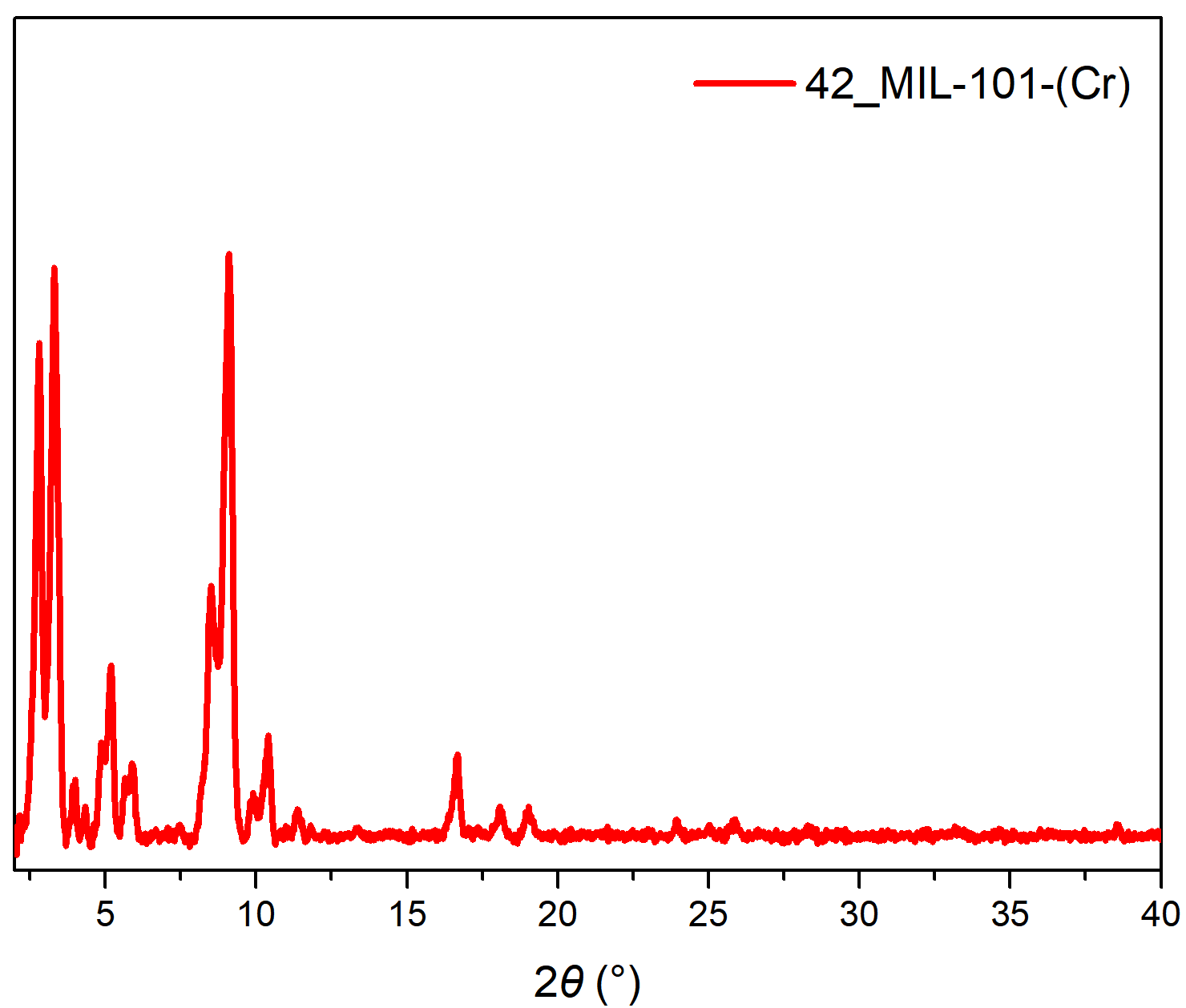
**

**Figure S6**. PXRD patterns for **42**

**
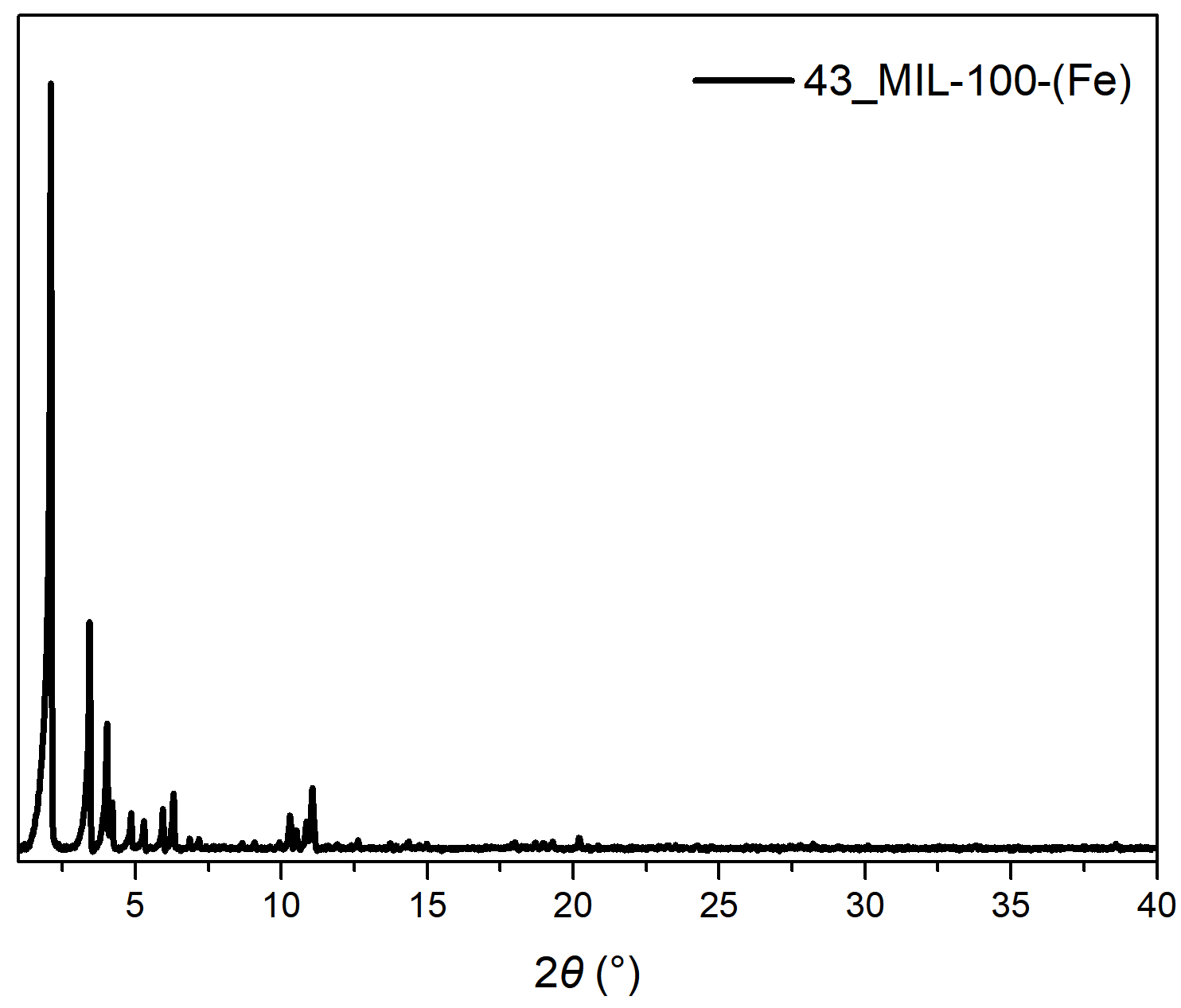
**

**Figure S7**. PXRD patterns for **43**

**
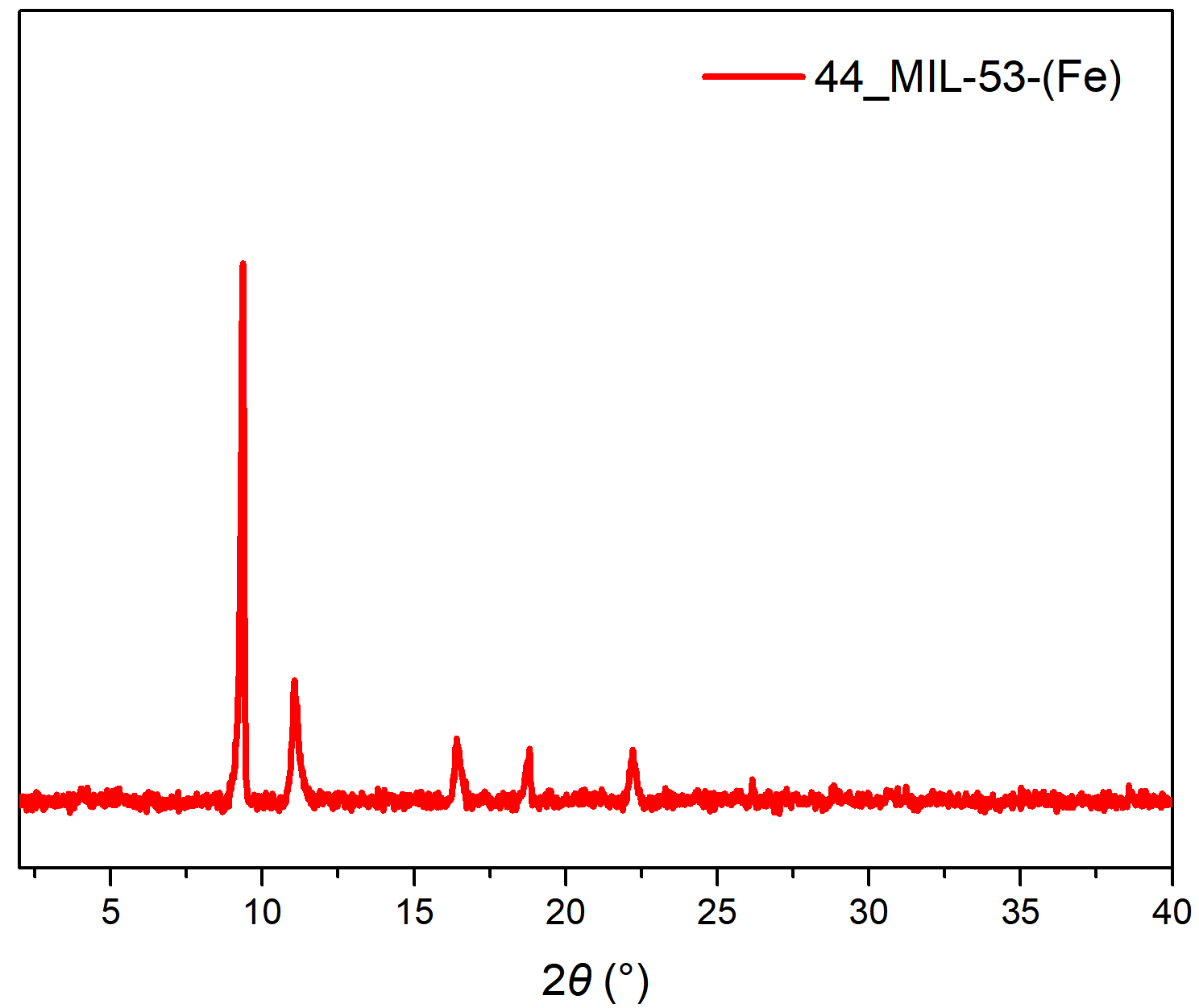
**

**Figure S8**. PXRD patterns for **44**

**
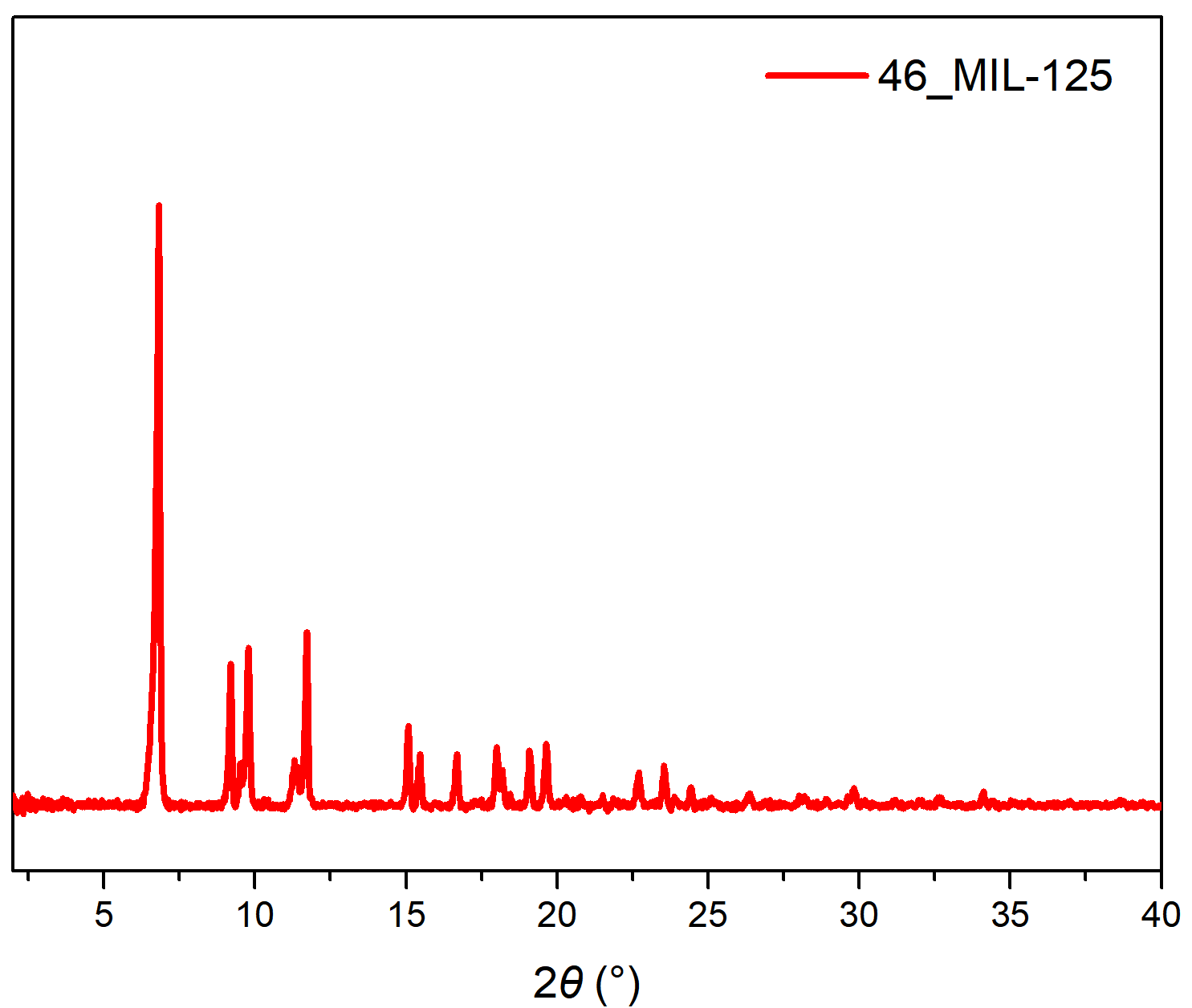

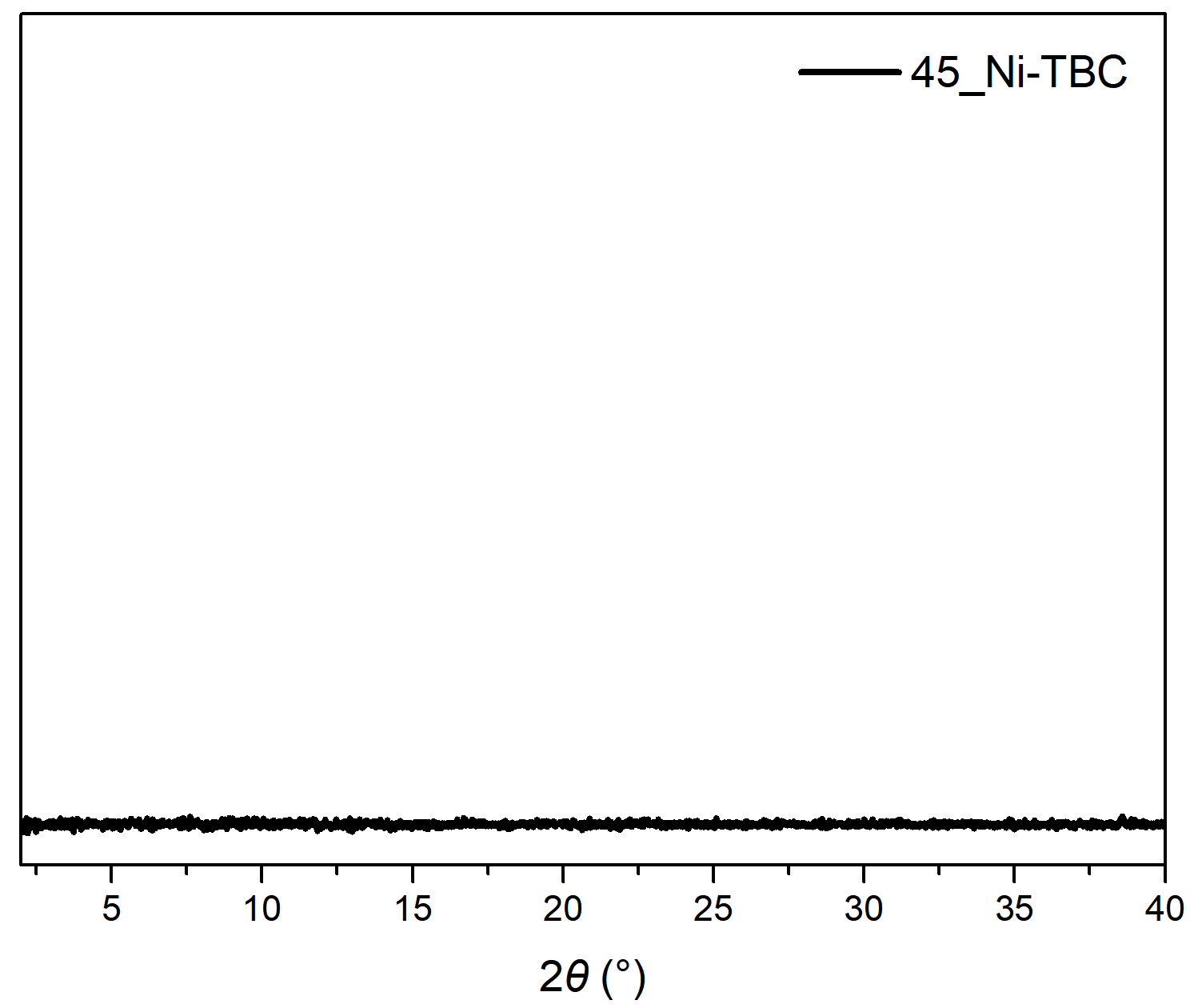
**

**Figure S10**. PXRD patterns for **46**

**Figure S9**. PXRD patterns for **45**. No distinct diffraction peaks were observed, indicating the absence of long-range crystallinity. Combined with the lack of gas uptake in N₂ adsorption measurements and no response in the colorimetric assay, these results confirm that material 45 is nonporous.

**
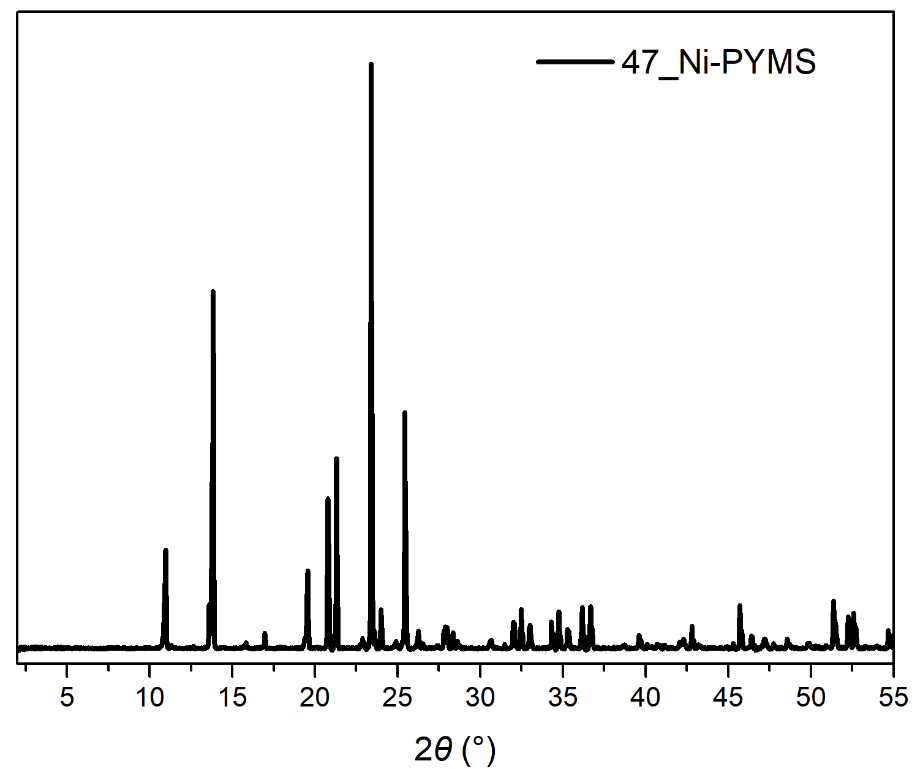
**

**Figure S11**. PXRD patterns for **47. 47** was confirmed to be nonporous based on its negligible gas uptake in N₂ adsorption measurements and minimal response in the colorimetric assay.

**
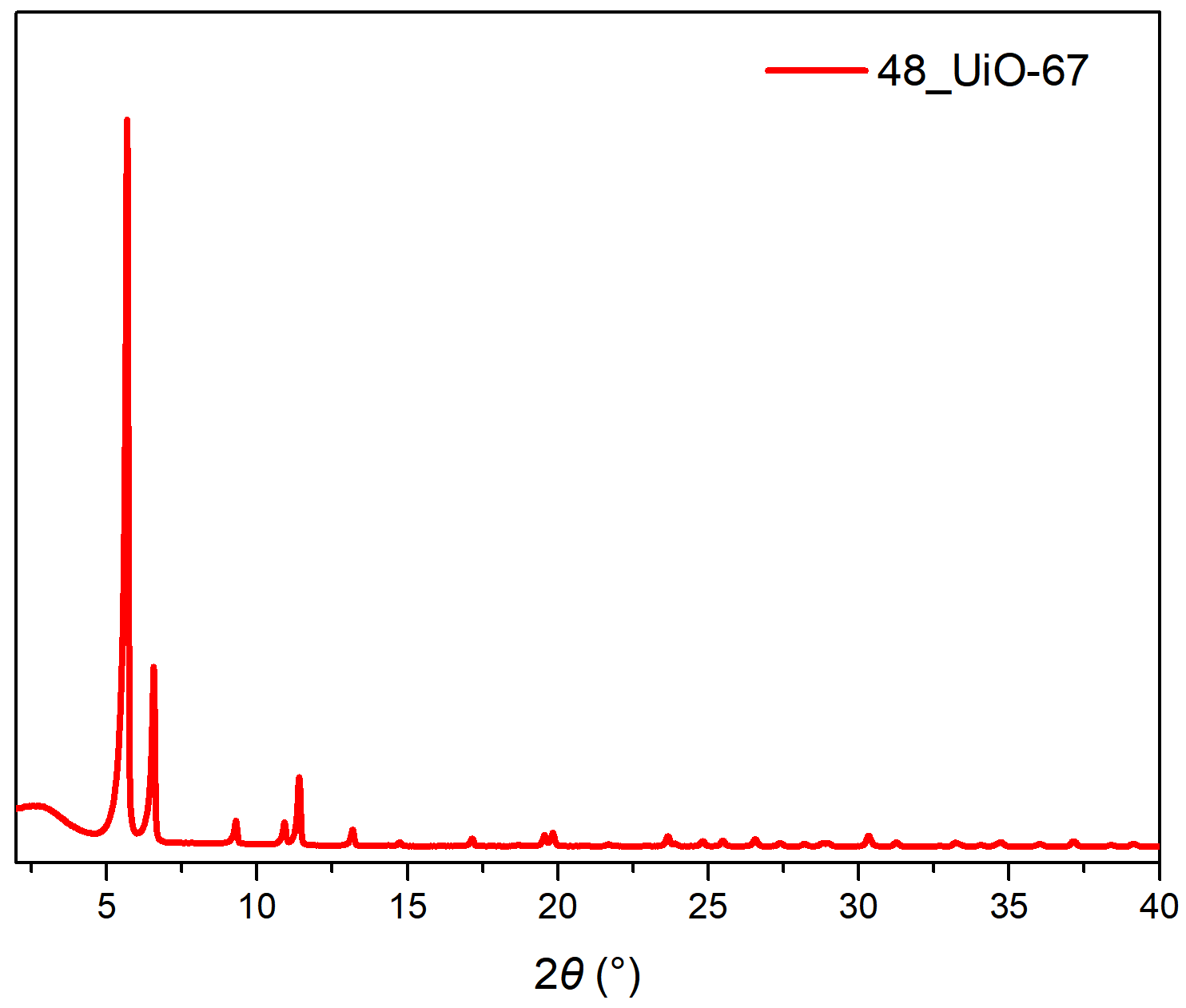
**

**Figure S12**. PXRD patterns for **48**

**
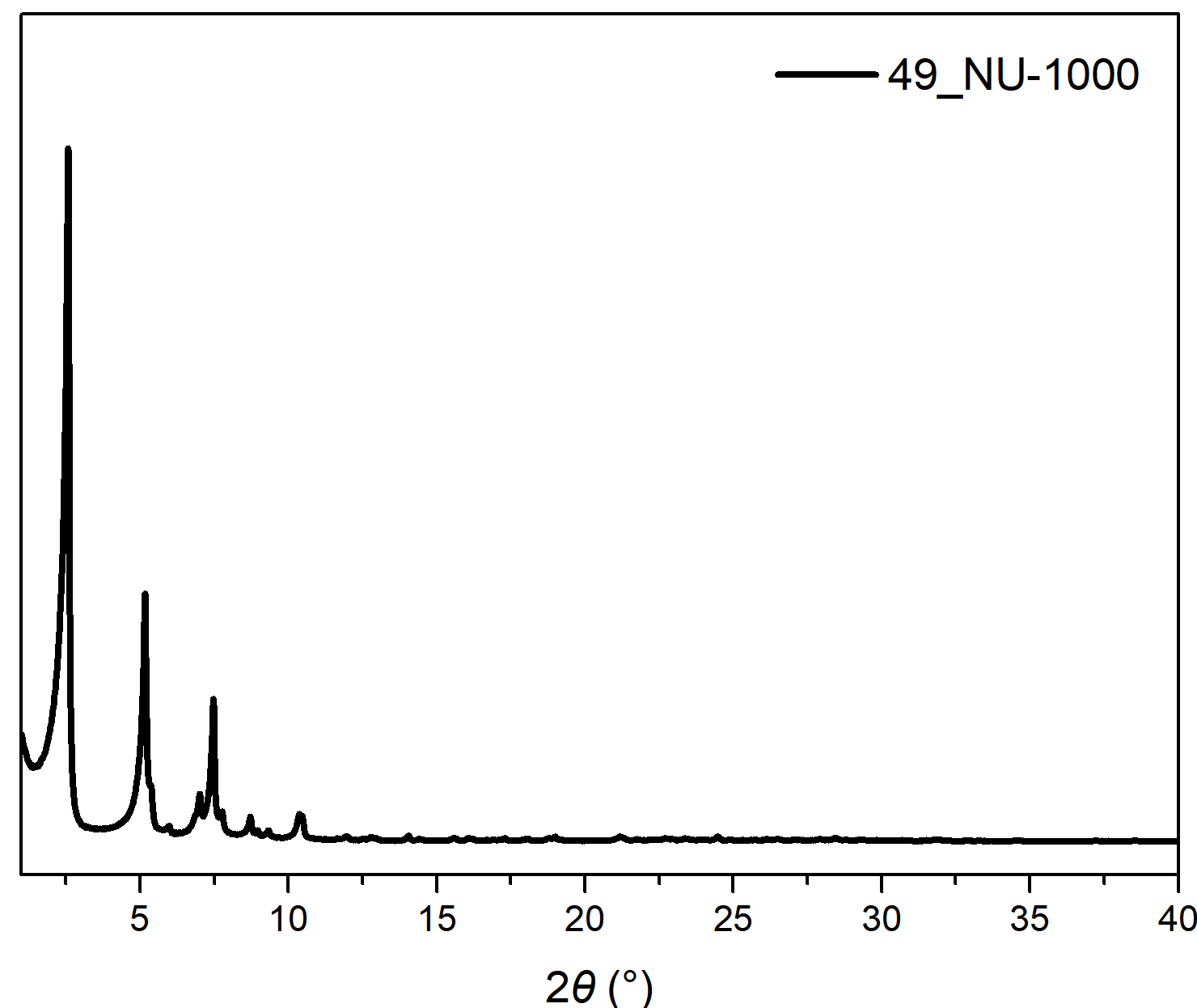
**

**Figure S13**. PXRD patterns for **49**

**
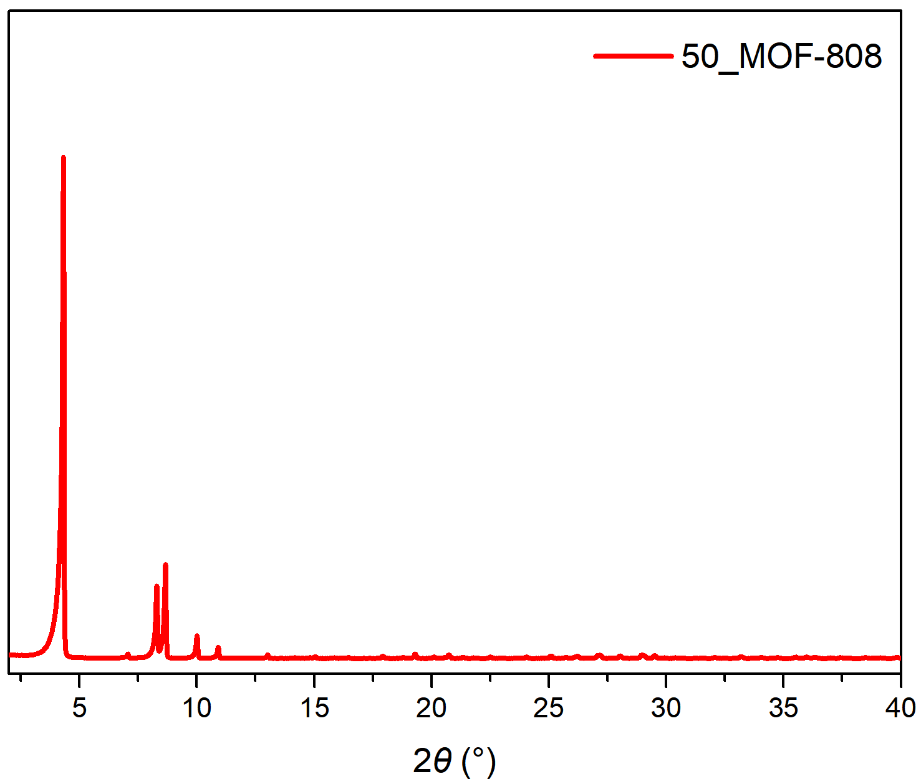
**

**Figure S14**. PXRD patterns for **50**

# **Experimental methods**

**Nuclear magnetic resonance spectroscopy**

NMR spectra were recorded on a Bruker 400 NMR spectrometer at 400 MHz (^1^H) and referenced against the residual ^1^H signal of the solvent.

**Fourier transform infrared**

FT-IR spectra were measured in transmission on a Bruker Alpha-P ATR FTIR (diamond crystal) at room temperature.

**Powder X-ray diffraction**

Laboratory powder X-ray diffraction (PXRD) data patterns were collected in transmission mode on samples held on thin Mylar film in aluminium well plates on a Panalytical Empyrean diffractometer, equipped with a high throughput screening (HTS) XYZ stage, X-ray focusing mirror, and PIXcel detector, using Cu-Kα radiation. Before measurement of dye adsorption, PXRD patterns of all crystallinity frameworks were acquired over a 2θ range of 1–40° with a step size of 0.013° and a total scan time of 10 minutes, or over a range of 2–60° with a step size of 0.05° for 18 minutes.

**UV-Vis measurements**

The UV-visible absorption spectra of the dye solution were recorded on a BioTek PowerWave HT

scanning microplate spectrophotometer.

**Gas uptake measurement**

Gases of the following purities were used: hydrogen (99.9995% - BOC gases) and carbon dioxide (SCF grade – BOC gases). Total pore volume and surface areas were measured by nitrogen adsorption and desorption at 77.3 K using a Micromeritics ASAP 2020 or Micromeritics 2420 volumetric adsorption analyzer. Samples were degassed offline at their activation temperature for 15 h under vacuum (10^-5^ bar) before analysis. Carbon dioxide isotherms were measured at 298 K using a Micromeritics 2420 volumetric adsorption analyzer using the same degassing procedure.

**Water sorption**

Water vapor isotherms were collected at 25 °C on a Micromeritics 3Flex. ~20 mg of sample was degassed under vacuum (<0.0133 mbar) for 12 h at 110 °C.

**Dye solution preparation**

The colorimetric array consisted of Lucifer yellow, ponceau xylidine, methyl orange, crystal violet, safranin O, acridine orange, coumarin 6, citrus red 2, and sudan I, all purchased from Sigma-Aldrich. Each dye sensor solution was prepared by dissolving 10 mg of dye powder in 1000 mL of water (or a solvent mixture of *n*-heptane and diethyl ether (98/2%) for the organic soluble dyes see Figure S28 for more details) to achieve a concentration of 10 ppm. The solutions were vigorously stirred at room temperature for 10 minutes and subsequently stored in the dark for later use.

**High throughput experiments by using semi-automation platforms**

The procedure begins with automated powder dispensing using a Quantos solid dispenser, where 6 mg of solid material (with a 5% error margin) is weighed into a 20 mL vial (Chemspeed Technologies). Each sample is dispensed into six vials. Next, both the samples and prepared stock dye solutions (stored in 500 mL jars) are loaded into the robotic platform, which automatically dispenses 9 mL of dye solution into each vial (see Figure S34 for Chemspeed SWING robotic platform configuration). When the liquid is added, septa capped vials, ensuring that the vial remains sealed during subsequent operations. The samples were then shaken in two modules at 500 rpm for 18 h at room temperature. Following shaking, the samples are processed through membrane filtration using a 0.22 µm pore size filter (polyethersulfone membrane for aqueous solutions and polytetrafluoroethylene membrane for non-aqueous solutions) to obtain the dye solution. 5 mL filtrate was then transferred into 9 mL sample vials for visual analysis. Last, images of the dye array were captured before and after exposure to the analyte. These images were processed using an in-house Python script discussed below.

**Computer vision scripts**

The Python scripts developed for image processing and concentration calculation, as well as the complete set of original images for all tested materials, are available in the project repository. You can access the code and data at: <https://github.com/yushuhankungfuGigi/Colorimetric_Dye_Array>

# **The Design of Dye Array**


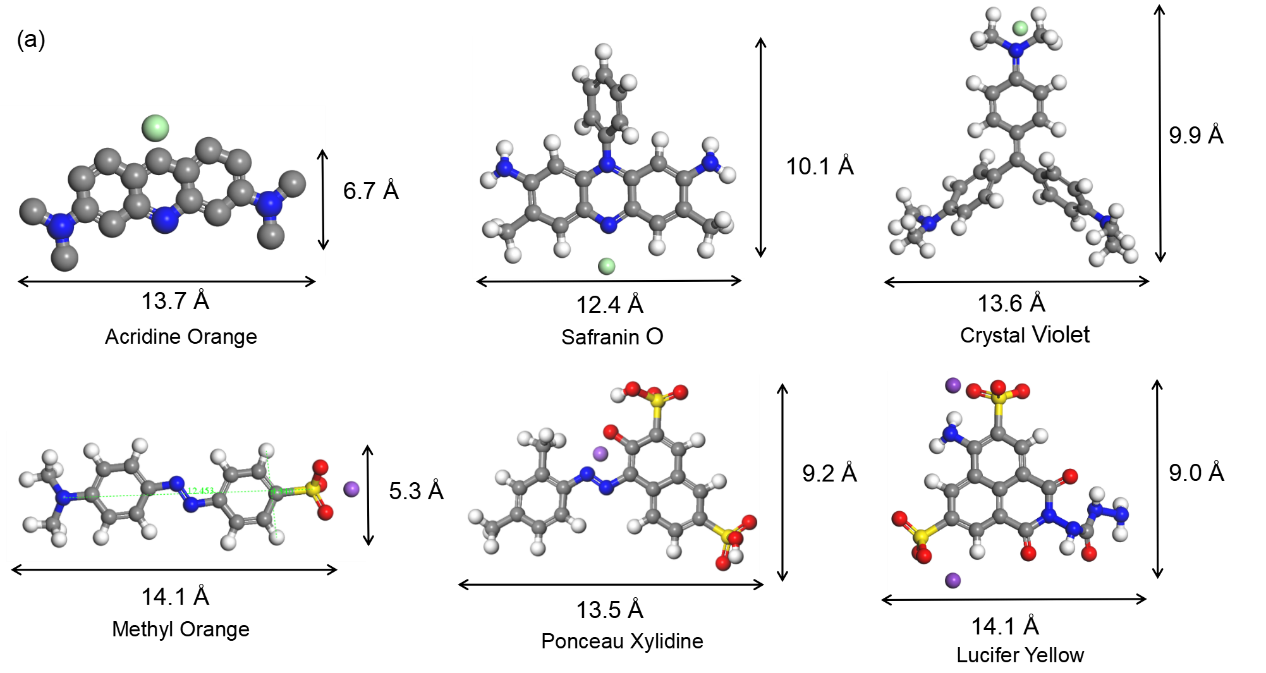


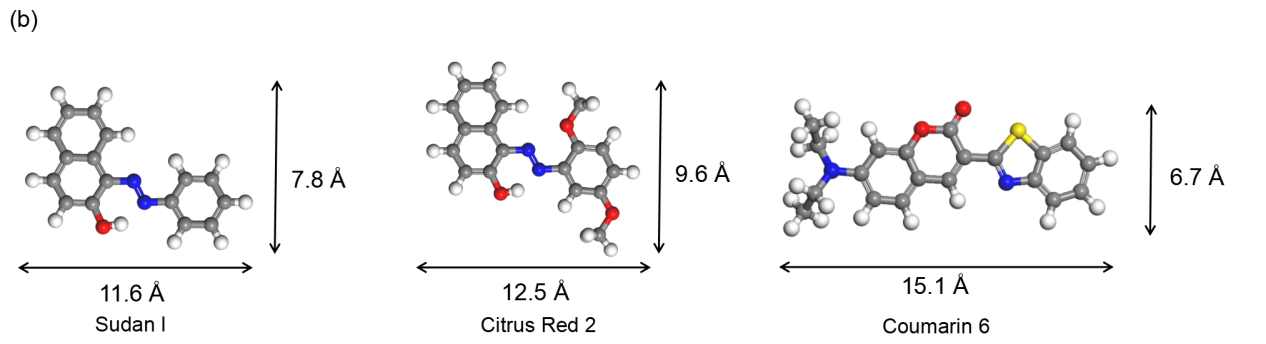
**Figure S15**. Geometry optimization and molecular dimensions of selected (a) water-soluble dye molecules and (b) oil-soluble dye molecules. Molecular structures were constructed using Materials Studio 7.0 (BIOVIA, licensed version). Geometry optimizations were performed using the Forcite module with the Ac6+3 forcefield library. The electrostatic interactions were treated with the atom-based electrostatic summation method, employing cubic spline truncation with a cutoff radius of 12.5 Å, a spline width of 1 Å, and a buffer width of 2 Å. All calculations were conducted using ADF2017 for post-optimization analysis where applicable.


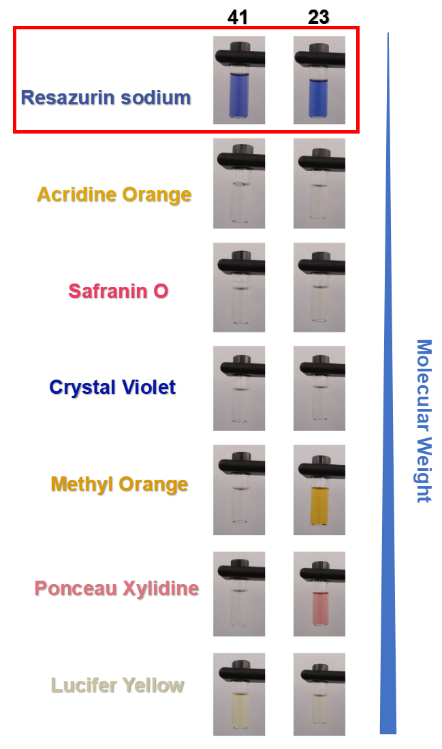
**a**

**
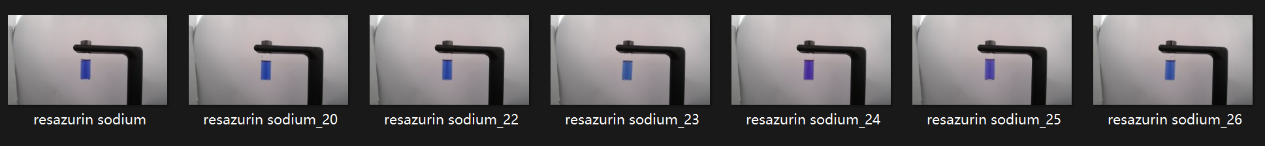
b.**

**Figure S16**. (a) Images of porous materials **41** (MIL-101-(Cr)-SO₃H) and **23** (CC19) after filtration after exposure to resazurin sodium. Compared to other dye probes, resazurin sodium exhibits low adsorption capacity and minimal color change on porous materials, making it an unsuitable indicator for porosity screening of porous materials. (b) Images of six organic cages after filtration with resazurin sodium. Similarly, resazurin sodium exhibits low adsorption capacity and minimal color change on porous materials (**20**, **22**, **23**, and **26**). Additionally, a slight color shift from blue to purple is observed on nonporous materials (**24** and **25**), further confirming its unsuitability as an indicator for porosity screening

**
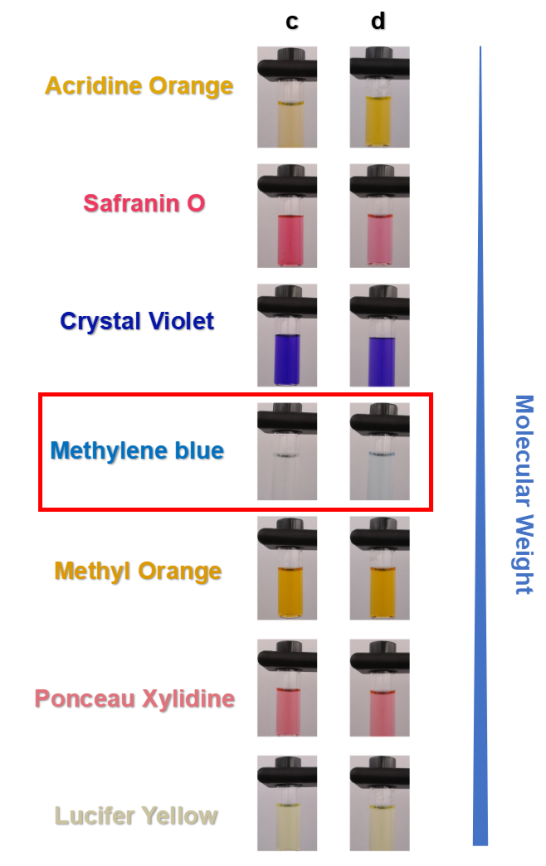
**

**Figure S17**. Images of polycaprolactone (c) and Tenax® Polymer (based on 2,6-diphenyl-p-phenylene oxide) (d) after filtration after exposure to methylene blue. Compared to other dye probes, methylene blue exhibits unusually high adsorption capacity and significant color change on non-porous materials, making it an unsuitable indicator for porosity screening of nonporous materials.


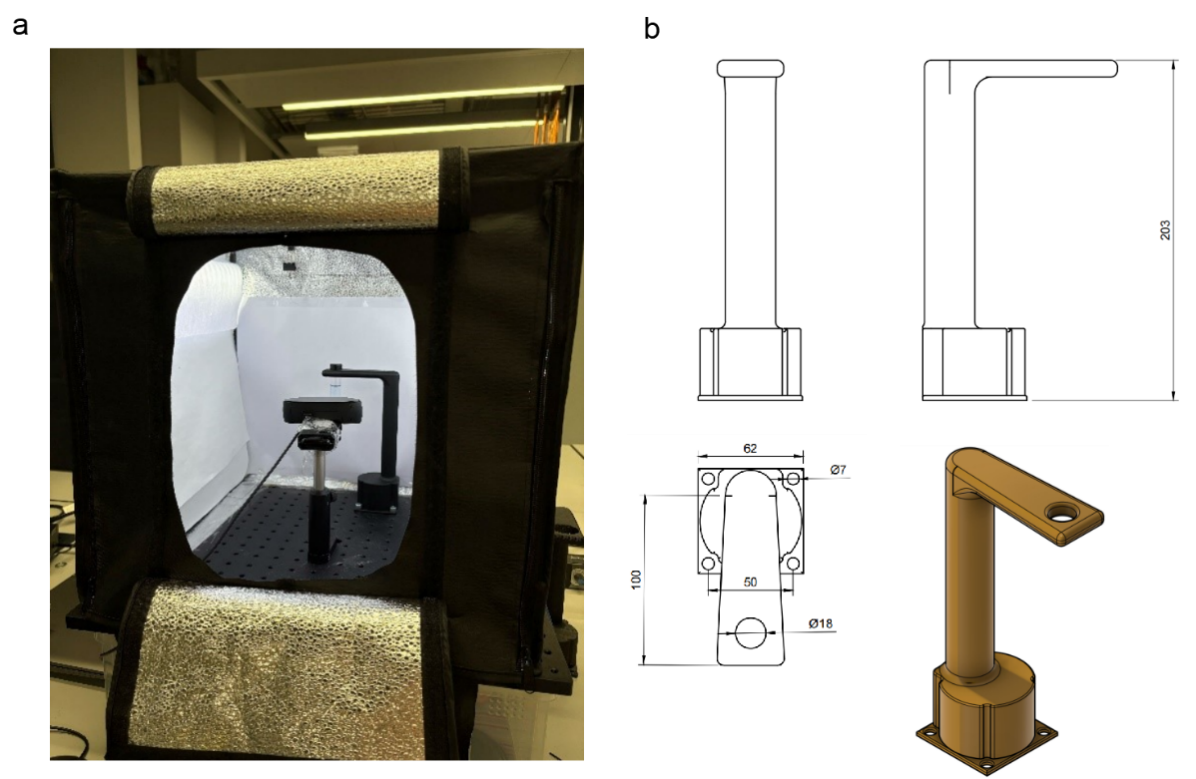


**Figure S18** (a) All images were captured using this imaging setup, housed within a controlled lighting environment. A black enclosure was used to ensure consistent illumination and to minimize peripheral light interference during the experimental procedures. Two 6 W light-emitting diodes placed on either side were connected to a power modulator to control light intensity. A web camera was positioned inside aligned with the sample holder. The camera, light sources, and sample holder were securely fixed to minimize environmental fluctuations. This setup ensures minimal interference from ambient light, simplifies image processing, and allows easy extraction of visual outputs using computer vision (CV) algorithms without the need for complex masking or segmentation. (b) A schematic illustration of the 3D-printed sample holder.

**
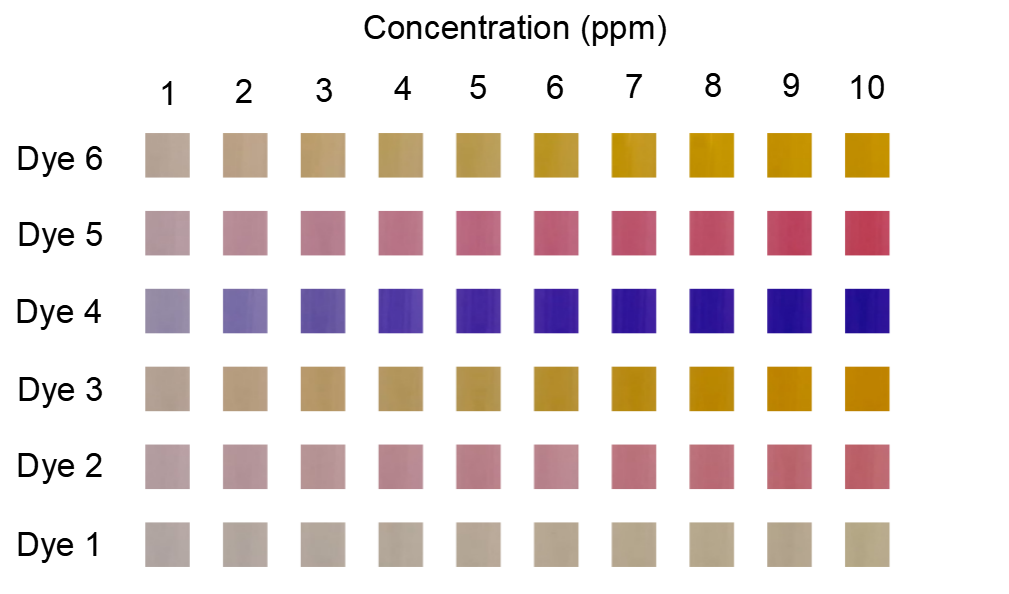
**


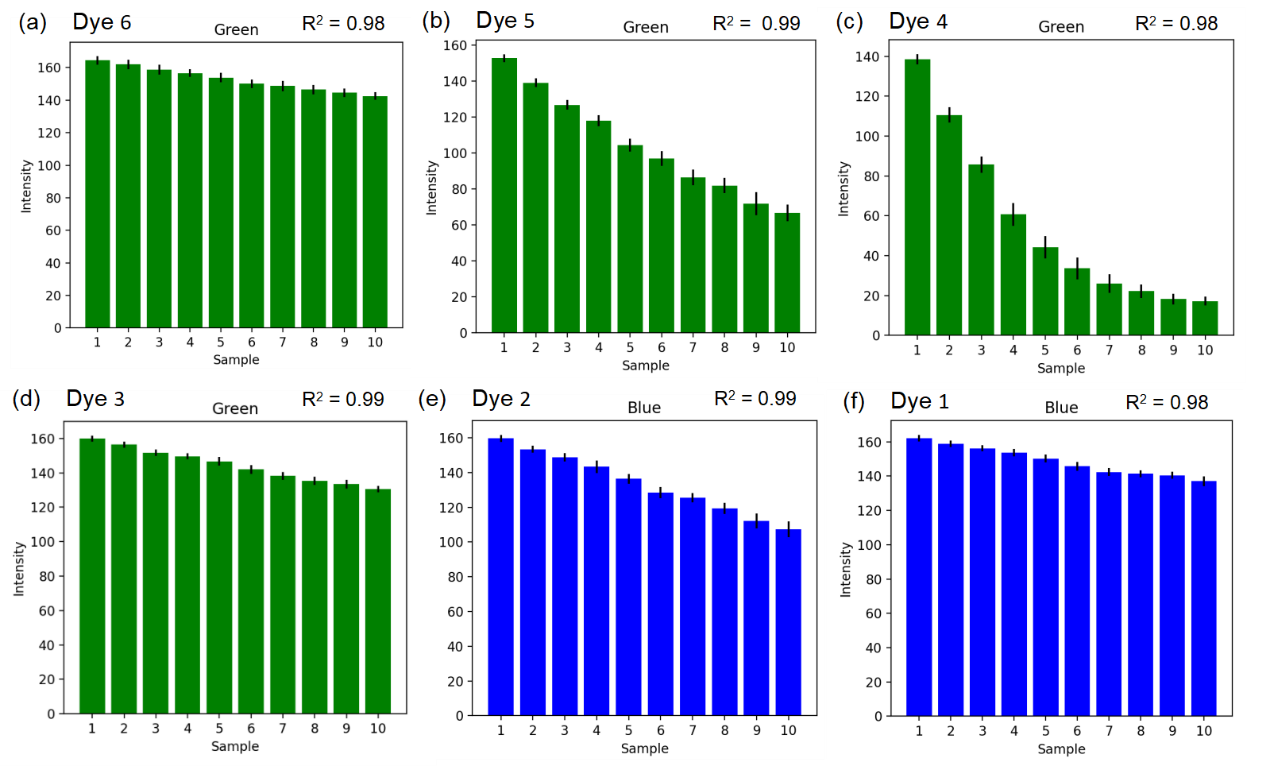


**Figure S19**. Images of six water-soluble dye solutions at concentrations ranging from 1–10 ppm

**
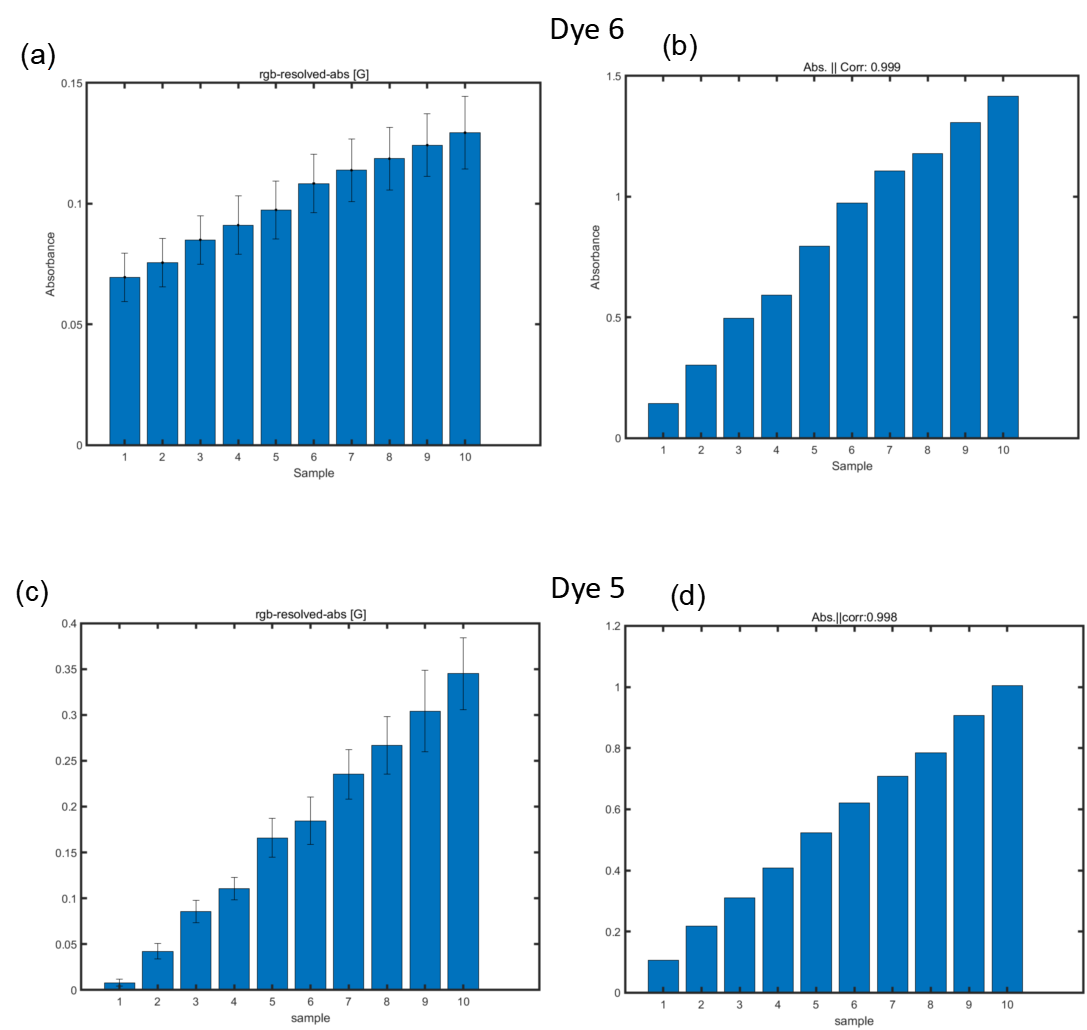
Figure S20**. Respective R, G, or B channel intensity sensitivity of six water-soluble dye solution at concentrations ranging from 1–10 ppm (sample No.1 to No.10)


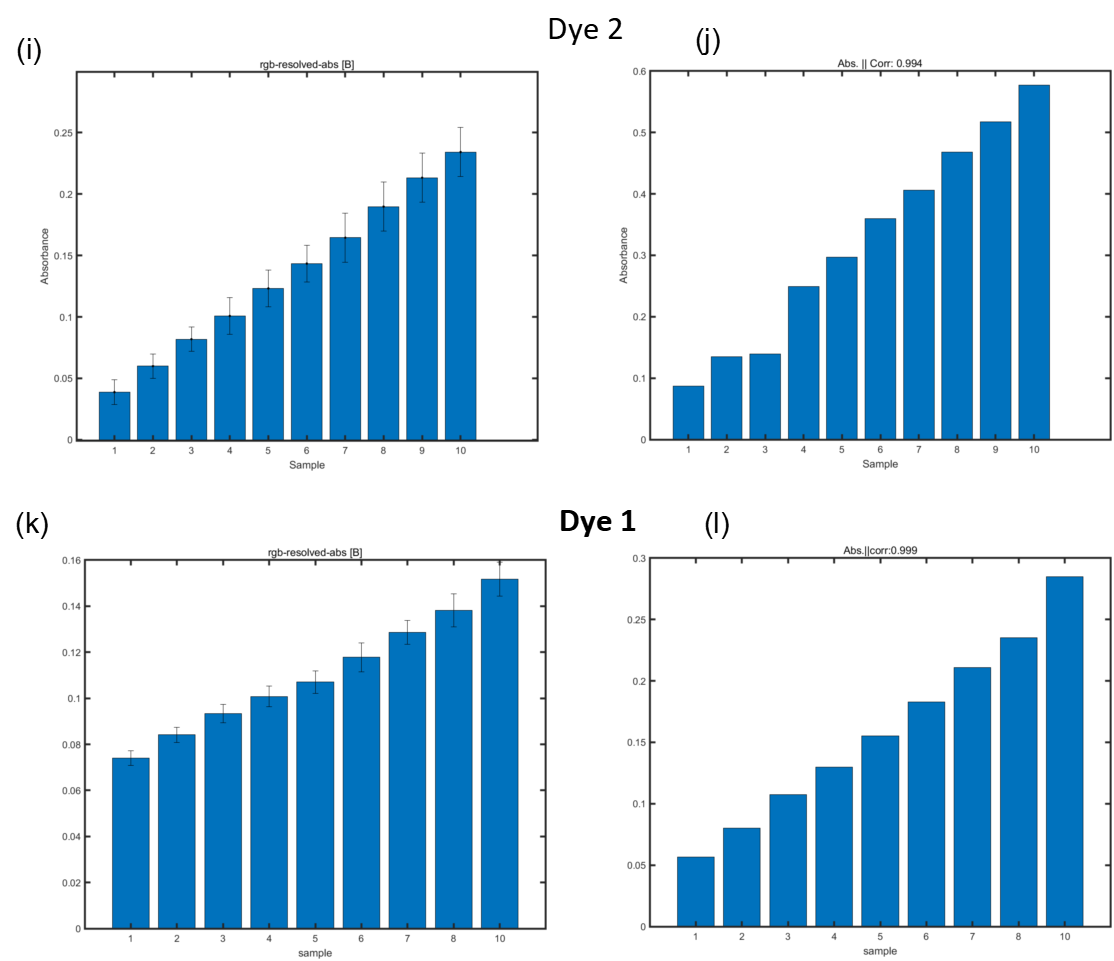

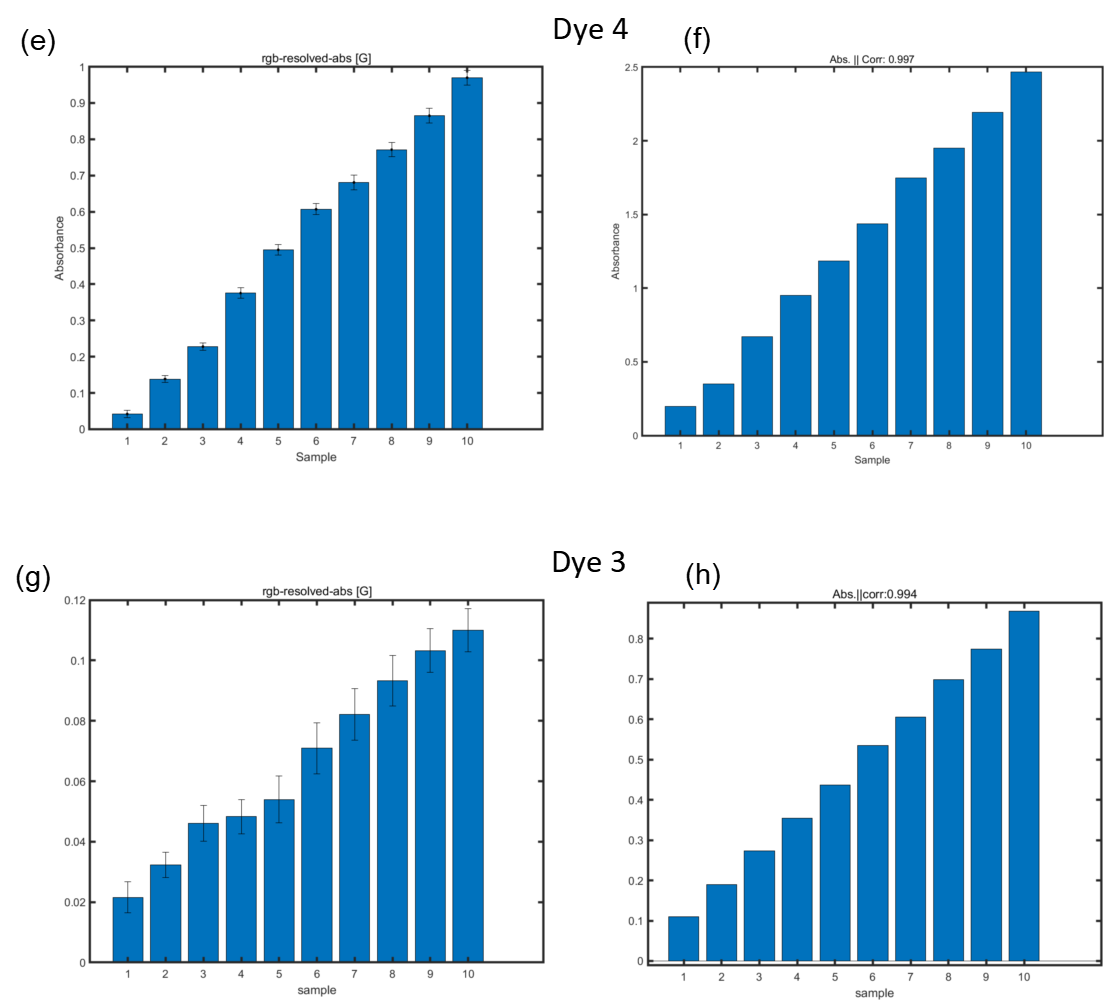


**Figure S21**. (a), (c), (e), (g), (i) and (k) UV-Vis spectroscopic analysis of all dye sensors at concentrations ranging from 1-10 ppm; (b), (f), (h), (j) and (i) Pearson correlation (>0.99) between the respective RGB channel intensity, and the maximum absorbance obtained from UV-Vis spectroscopic analysis

**Figure S22** (a), (b), (c), (d), (e), (f) Respective R, G, or B channel intensity sensitivity (Top) and L*, a* and b* channel intensity sensitivity (Bottom) of six water-soluble dye solution at concentrations ranging from 0–0.5 ppm.


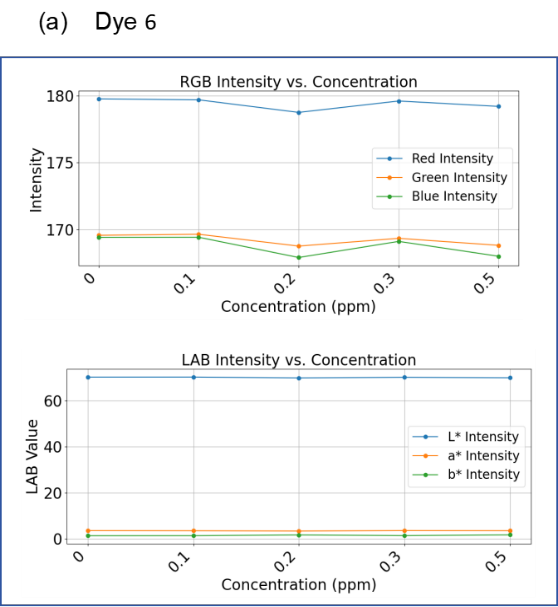

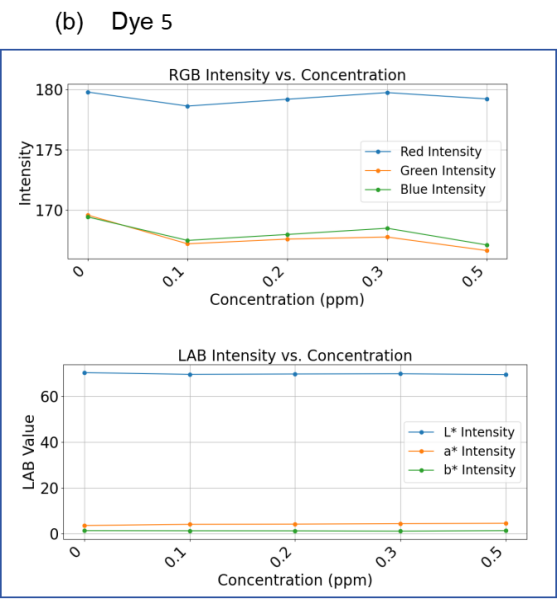

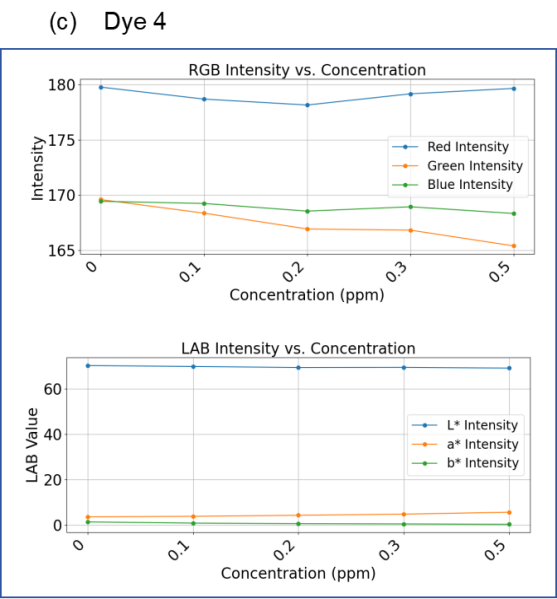

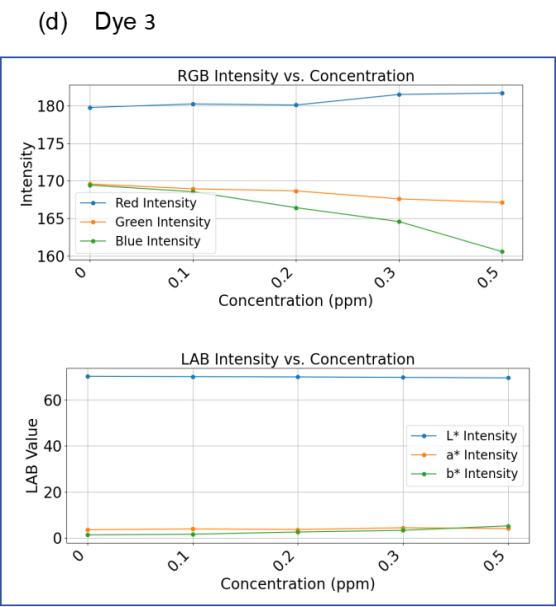

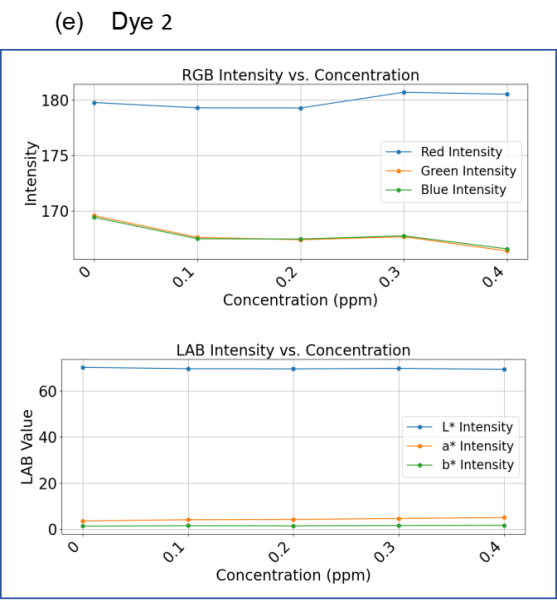

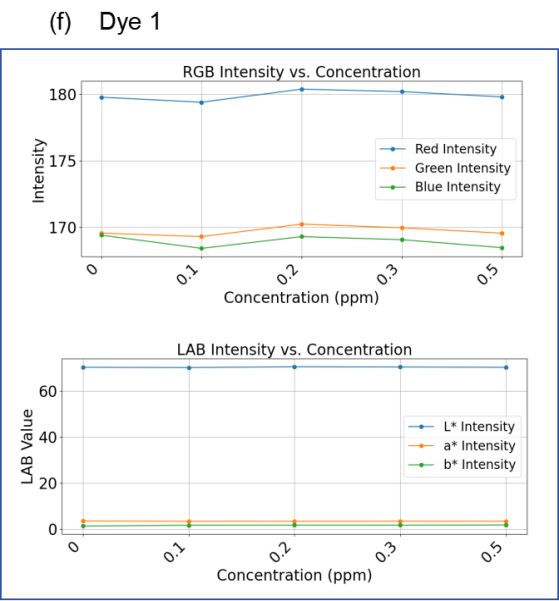


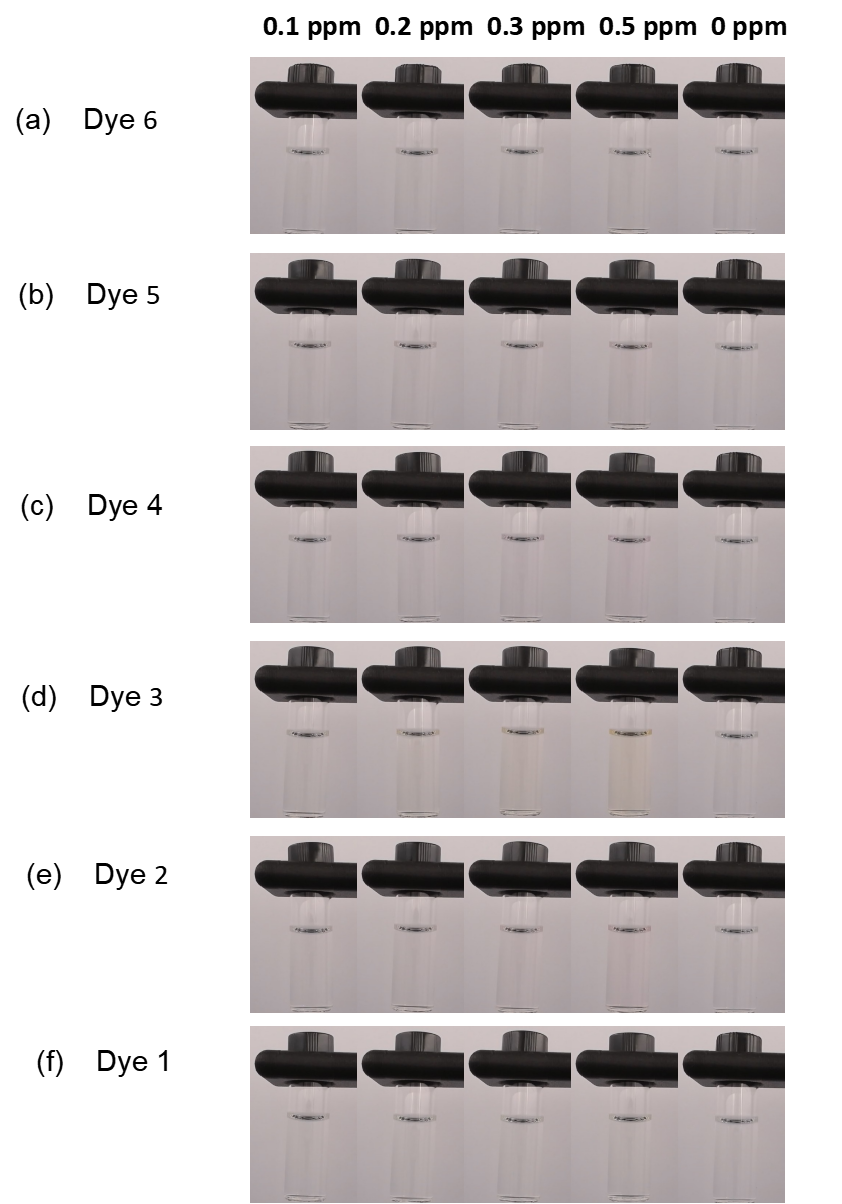


**Figure S23** (a), (b), (c), (d), (e), (f) Images of six water-soluble dye solution at concentrations ranging from 0–0.5 ppm. Calibration were curves generated using the RGB channels demonstrated a nearly linear response in the concentration range of 1 to 10 ppm (Figure S20). However, in the lower concentration range (below 1 ppm), the RGB signals became nearly identical (Figure S22), likely due to the limited sensitivity of the imaging sensor at very low analyte concentrations. Specifically, the optical density of samples below 1 ppm is insufficient to produce a measurable change in the RGB values, resulting in a non-linear response. Although this behavior indicates that the camera cannot reliably distinguish between 0 ppm and low ppm levels, this does not compromise the method’s efficacy for our intended application. Since samples below 1 ppm are visually indistinguishable from a blank (water), they are classified as full color fade (hit), and the calibration curve is applied only within the effective dynamic range (≥1 ppm).

# **Experimental results**


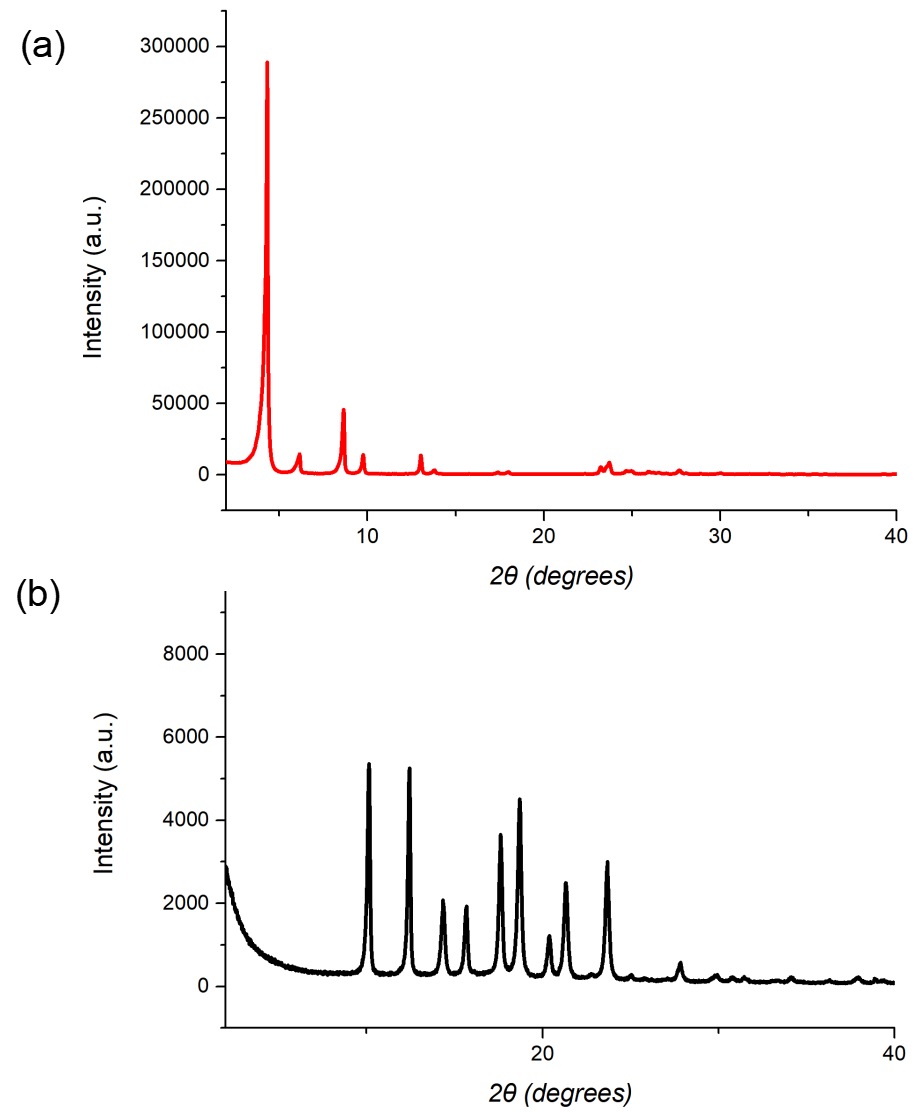
**Figure S24**. PXRD patterns of (a) **14** (TBAPy-α) and (b) **13** (TBAPy)


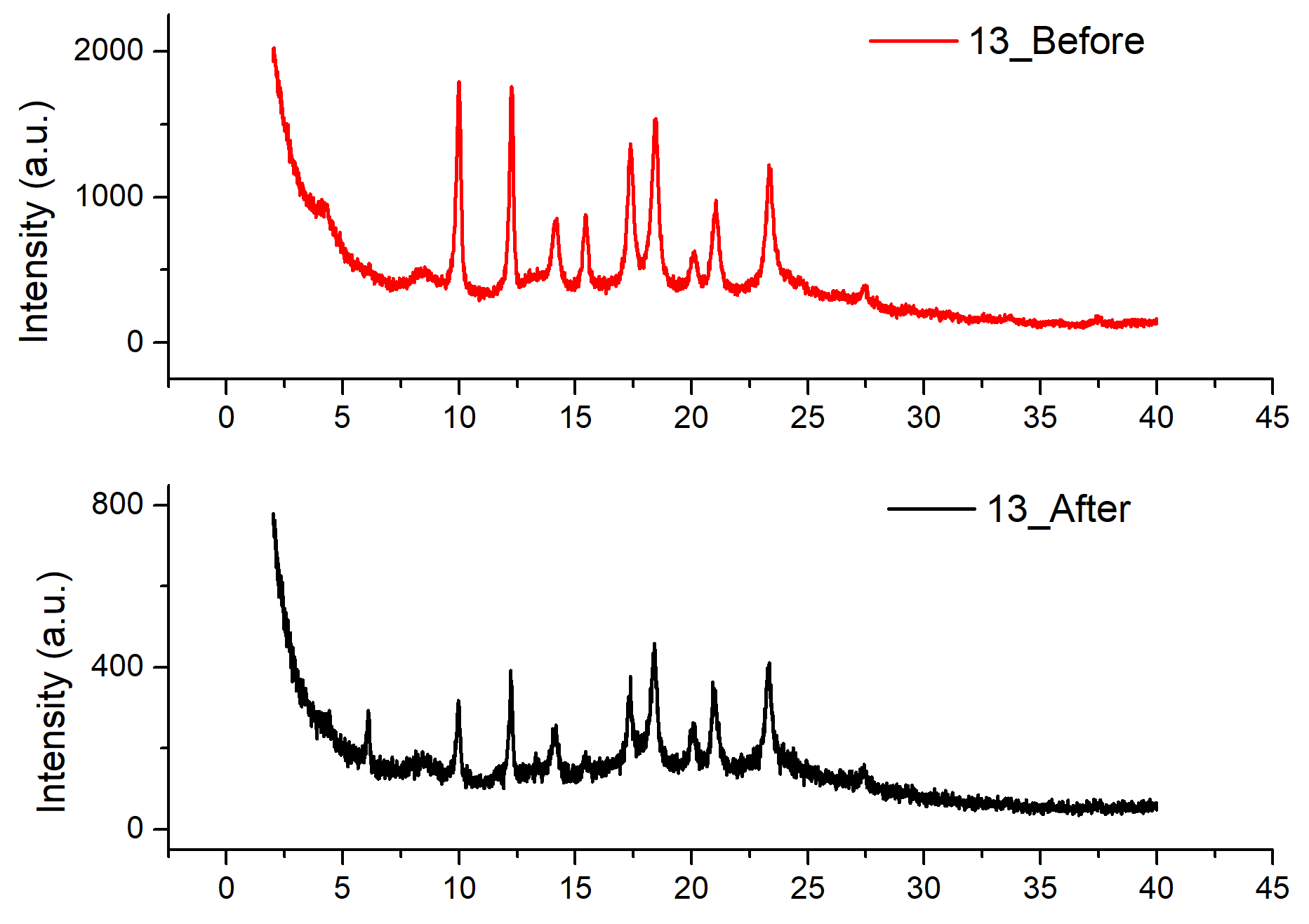


**Figure S25**. PXRD patterns of **13** before dye adsorption (top) and after dye adsorption (bottom). Due to the low sample amount used for each dye solution, we collected all used materials from all six dye solutions and combined them there. Following filtration and drying under vacuum at 80 °C, a PXRD pattern of the recovered material was recorded.


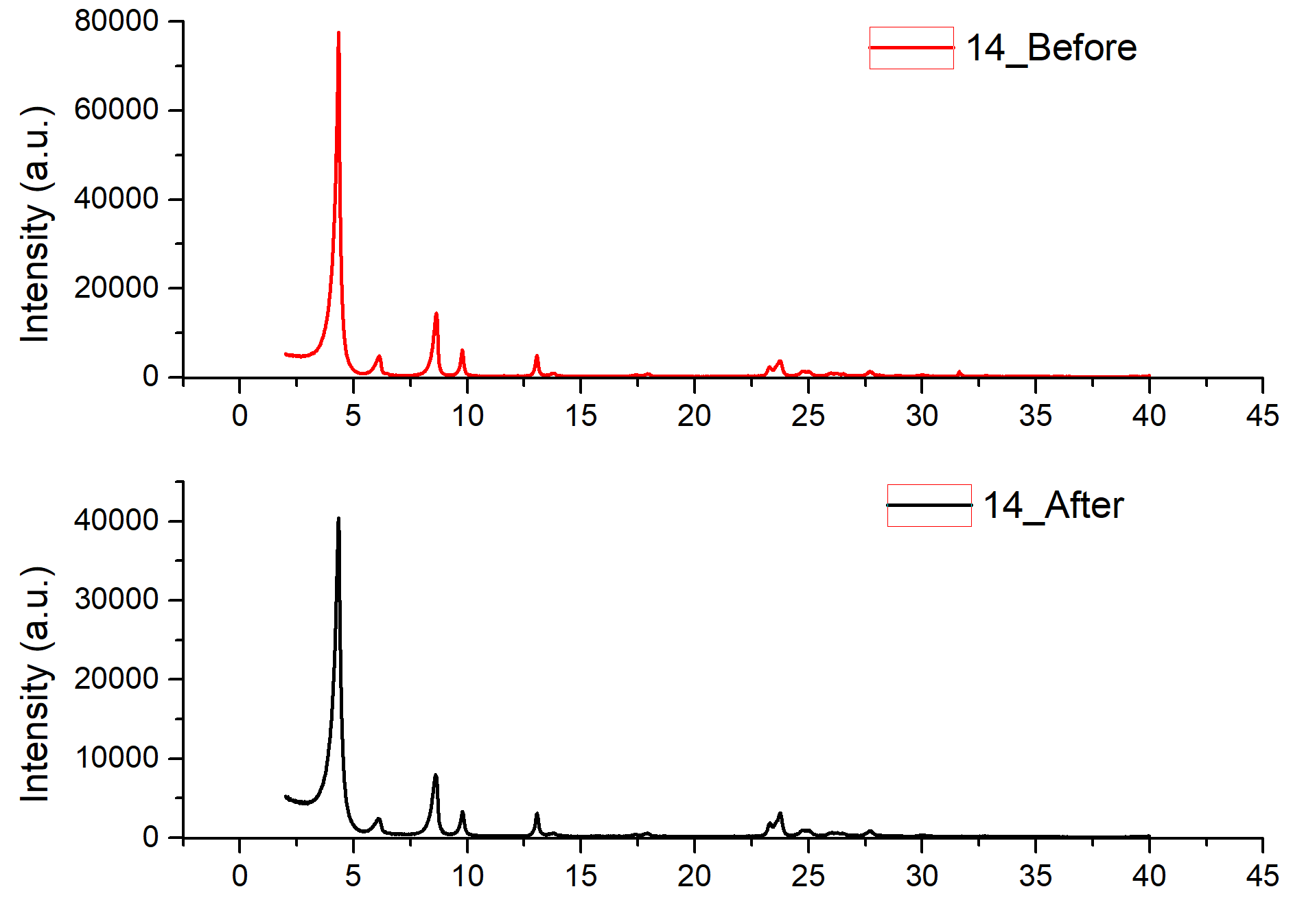


**Figure S26**. PXRD patterns of **14** before dye adsorption (top) and after dye adsorption (bottom). Due to the low sample amount used for each dye solution, we collected all used materials from all six dye solutions and combined them there. Following filtration and drying under vacuum at 80 °C, a PXRD pattern of the recovered material was recorded.


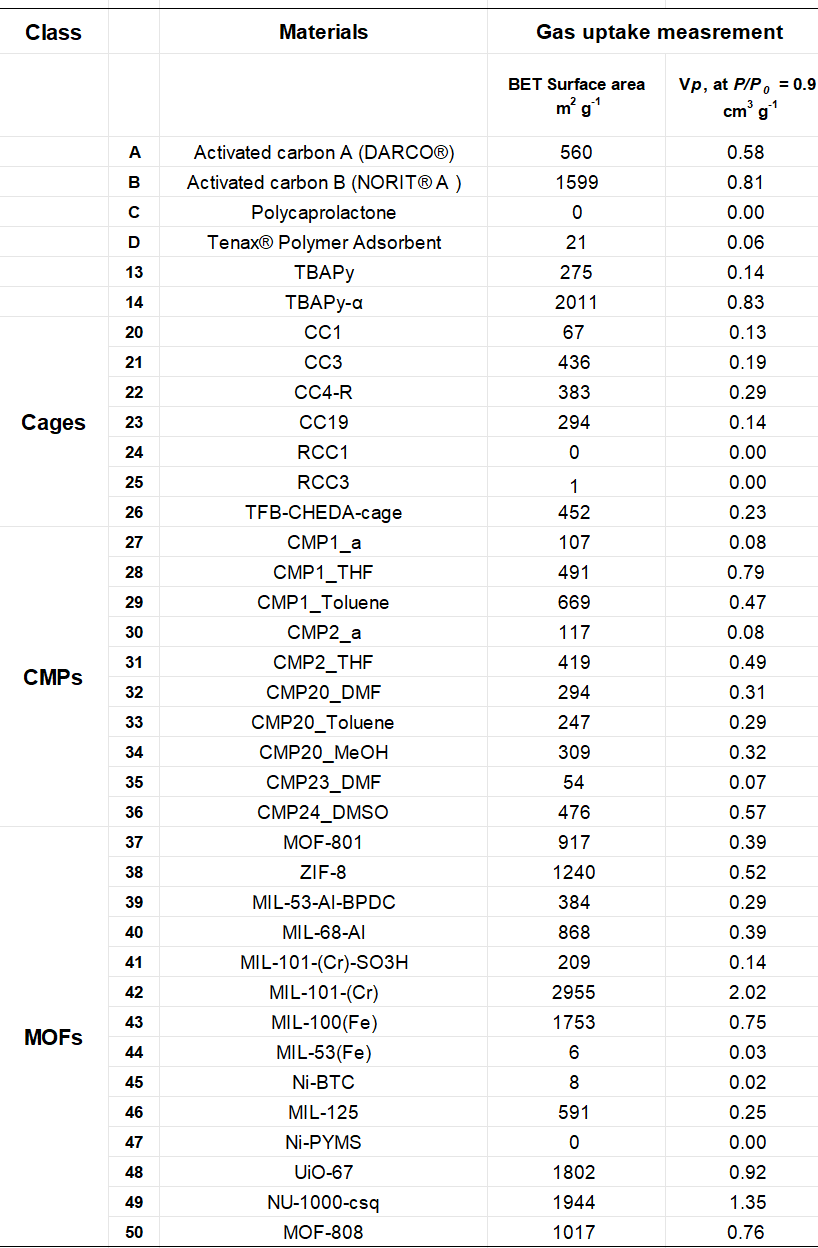

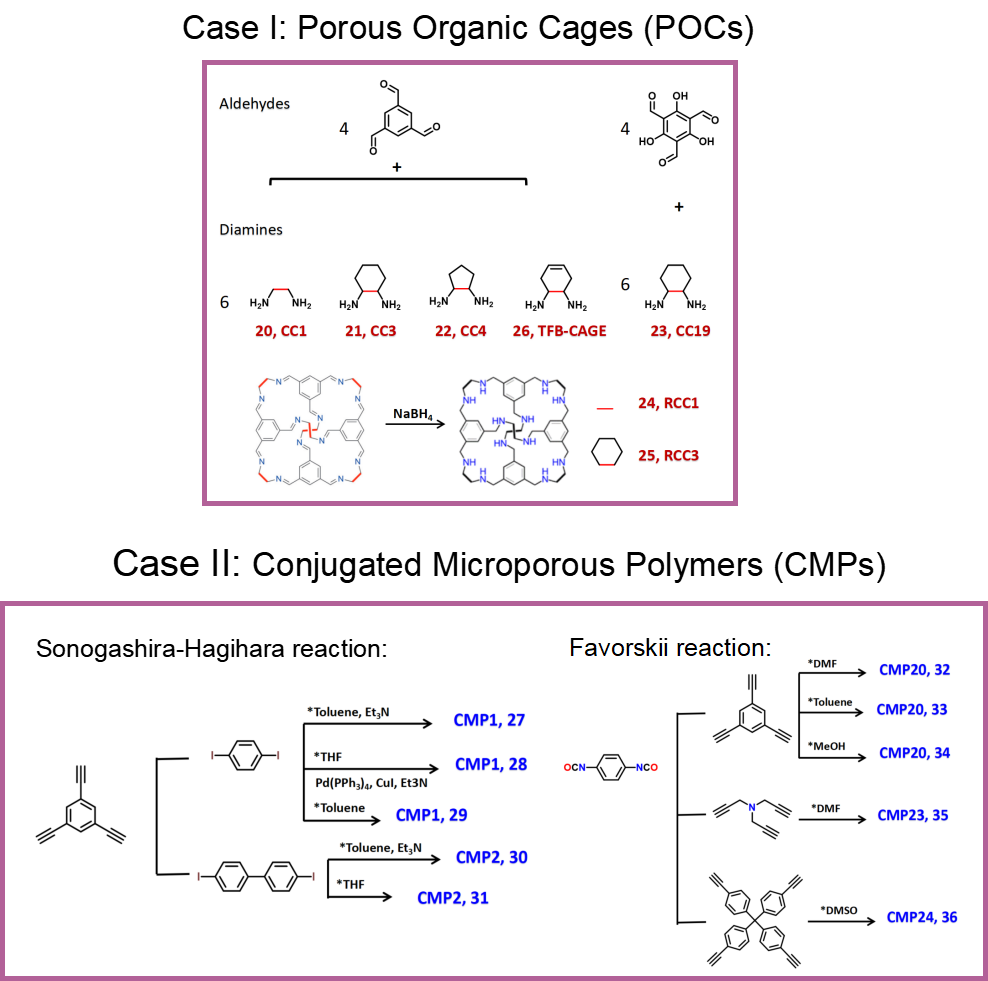


**Table S2**. BET surface areas and total pore volumes of the materials tested, as measured by nitrogen adsorption at 77 K.

**Figure S27** The structures and constituent linkers of (a) POCs (**20– 26**); and (b) CMPs (**27–35**).

**Table S3**. The metal cores and organic ligands of the MOFs studied here


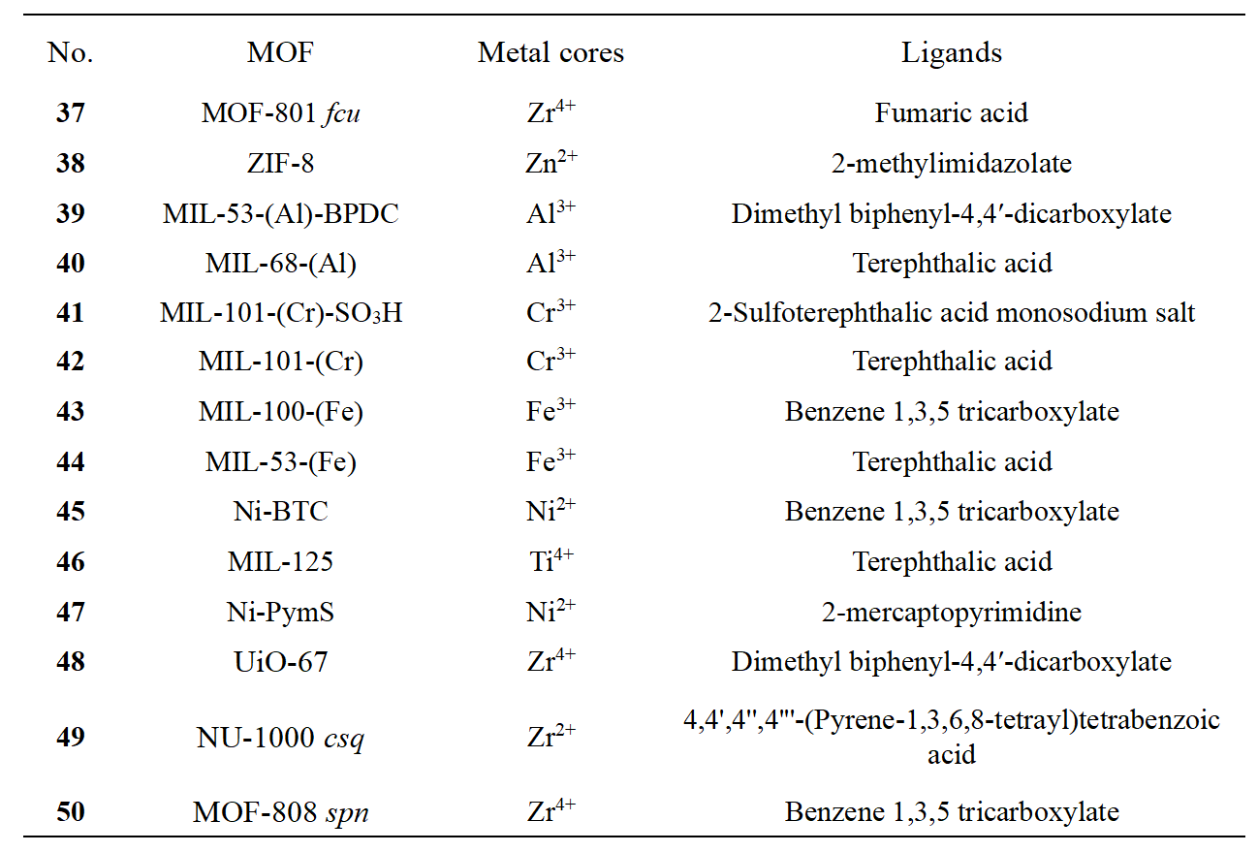


**Customized dye array**

Beyond their variations in porosity, the MOFs also showed a range of hydrophilic and hydrophobic properties. In MOFs, factors such as open metal sites, metal nodes, and organic linkers play a critical role in determining their hydrophobicity, which further influences their adsorption performance. Here, we customized a subarray with oil-soluble formulations to address materials with varying hydrophilicity. This customized subarray of three dyes was used in conjunction with the parent array of six sensors.

However, based on the colorimetric map in Figure S28, the oil-soluble dye pool contributed little to distinguishing the porosity of these 14 MOFs. We therefore chose not to include it in the main screening method.


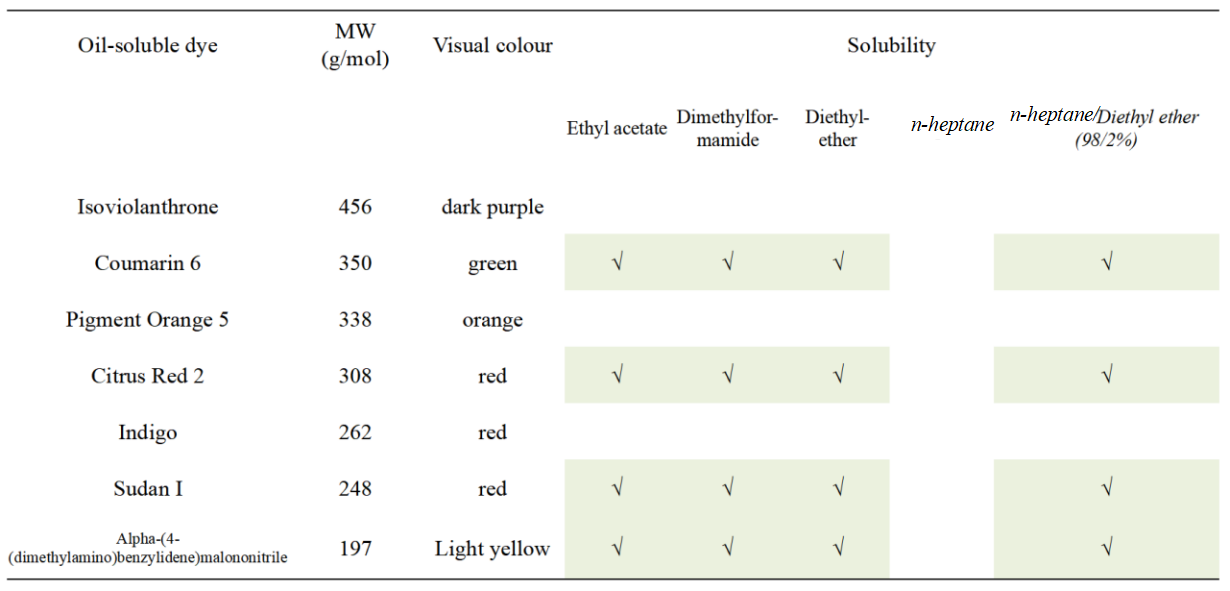


**Table S4**. The solubility of potential hydrophobic dye candidates in different solvents solvents

* In accordance with the dye selection criteria outlined in Figure 1a, three dye molecules with varying molecular weights that demonstrate good solubility in the mixed solvents were selected. Alpha-(4-(dimethylamino)benzylidene)malononitrile was not selected into the designed array due to their light color changes that are not easy to observe.

**Table S5**. Polarity index of solvents

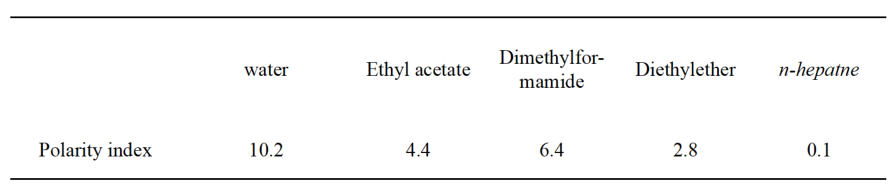


*As shown in Table S3 and S4, due to low polarity of n-heptane and good solubility of diethyl ether, a solvent mixture of n-heptane and diethyl ether (98/2%) was employed to simulate a non-polar, hydrophobic environment.


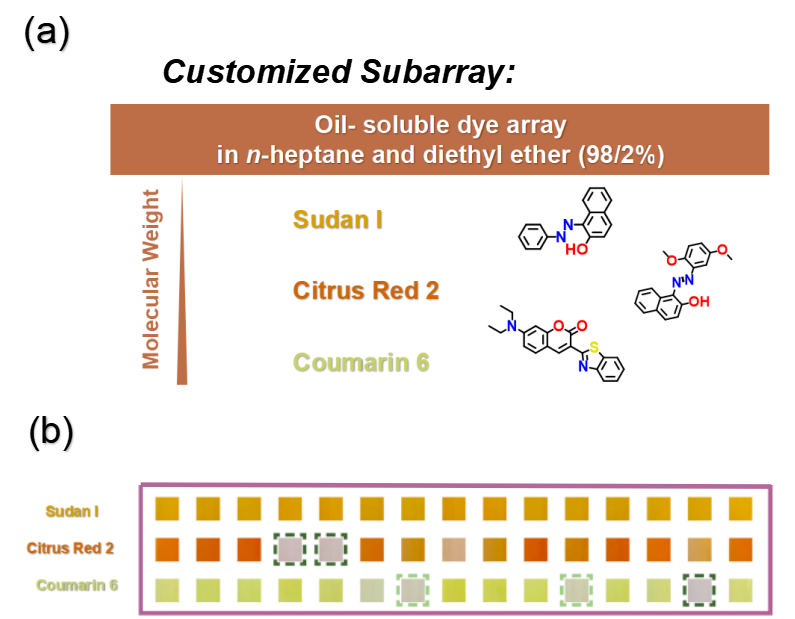


**Figure S28** (a) An oil-soluble dye pool was implemented as a compensation solution for MOFs with diverse hydrophilicities. Considering polarity and solubility (Tables S3 and S4), a solvent mixture of n-heptane and diethyl ether (98/2%) was employed to simulate a non-polar, hydrophobic environment. In accordance with the dye sensor selection criteria outlined in Figure 1a, three dye molecules with varying molecular weights that demonstrate good solubility in the mixed solvents were selected. (b) Colorimetric maps for MOFs analyzed using this subarray.


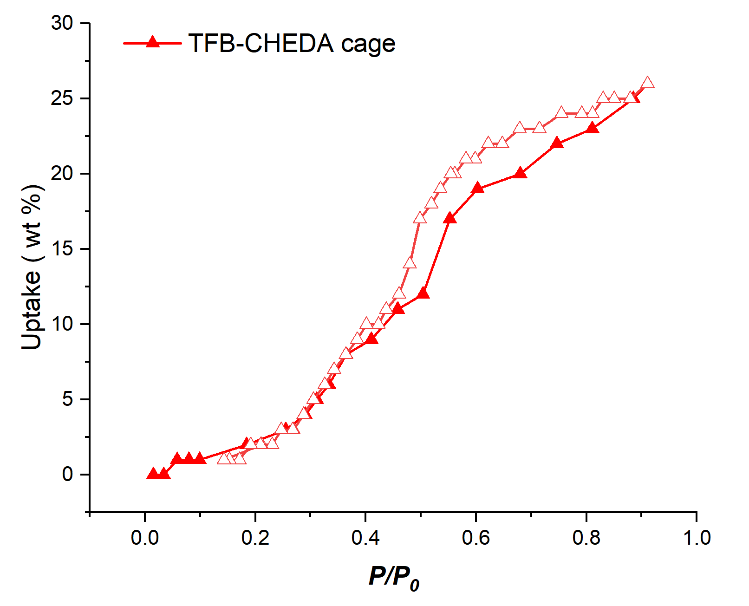


**Figure S29** Water adsorption isotherm for the TFB-CHEDA cage (26). The solid triangle represents the adsorption branch, and the hollow triangle represents the desorption branch.

**Table S6**. Target ROI channel intensity and corresponding concentration for each cage (**20**–**26**)

| Acridine  Orange | Materials_dye X | NO. | Target Main G | Target C (ppm) |
| --- | --- | --- | --- | --- |
|  | Cage_dye6 | **20** | 162 | < 1 |
|  |  | **21** | 161 | < 1 |
|  |  | **22** | 157 | 1.2 |
|  |  | **23** | 164 | < 1 |
|  |  | **24** | 142 | 8.4 |
|  |  | **25** | 153 | 3.4 |
|  |  | **26** | 162 | < 1 |
|  |  |  |  |  |
| Safranin O | Cage_dye5 | **NO.** | Target Main G | Target C (ppm) |
|  |  | **20** | 162 | < 1 |
|  |  | **21** | 142 | 1.5 |
|  |  | **22** | 161 | < 1 |
|  |  | **23** | 162 | < 1 |
|  |  | **24** | 102 | 5.7 |
|  |  | **25** | 79 | 8.1 |
|  |  | **26** | 102 | 5.7 |
|  |  |  |  |  |
| Crystal Violet | Cage_dye4 | **NO.** | Target Main G | Target C (ppm) |
|  |  | **20** | 161 | < 1 |
|  |  | **21** | 161 | < 1 |
|  |  | **22** | 144 | < 1 |
|  |  | **23** | 161 | < 1 |
|  |  | **24** | 77 | 3.1 |
|  |  | **25** | 32 | 6.7 |
|  |  | **26** | 161 | < 1 |
|  |  |  |  |  |
| Methyl  Orange | Cage_dye3 | **NO.** | Target Main G | Target C (ppm) |
|  |  | **20** | 130 | 9.7 |
|  |  | **21** | 129 | 10.1 |
|  |  | **22** | 130 | 9.8 |
|  |  | **23** | 134 | 8.7 |
|  |  | **24** | 129 | 10.1 |
|  |  | **25** | 138 | 7.4 |
|  |  | **26** | 134 | 8.7 |
|  |  |  |  |  |
| Ponceau  Xylidine | Cage_dye2 | **NO.** | Target Main B | Target C (ppm) |
|  |  | **20** | 114 | 8.7 |
|  |  | **21** | 116 | 8.4 |
|  |  | **22** | 113 | 8.9 |
|  |  | **23** | 110 | 9.4 |
|  |  | **24** | 128 | 6.4 |
|  |  | **25** | 127 | 6.5 |
|  |  | **26** | 116 | 8.4 |
|  |  |  |  |  |
| Lucifer  Yellow | Cage_dye1 | **NO.** | Target Main B | Target C (ppm) |
|  |  | **20** | 147 | 6.1 |
|  |  | **21** | 138 | 9.3 |
|  |  | **22** | 137 | 9.7 |
|  |  | **23** | 150 | 5.1 |
|  |  | **24** | 140 | 8.5 |
|  |  | **25** | 136 | 9.9 |
|  |  | **26** | 141 | 8.1 |
|  |  |  |  |  |

**Table S7**. Target ROI channel intensity and corresponding concentration for each CMP (**27**–**36**)

| Materials_dye X |  |  |  |
| --- | --- | --- | --- |
| CMP_dye6 | NO. | Target Main G | Target C (ppm) |
|  | 27 | 148 | 5.3 |
|  | 28 | 165 | < 1 |
|  | 29 | 165 | < 1 |
|  | 30 | 153 | 3.2 |
|  | 31 | 165 | < 1 |
|  | 32 | 165 | < 1 |
|  | 33 | 165 | < 1 |
|  | 34 | 164 | < 1 |
|  | 35 | 157 | 1.6 |
|  | 36 | 165 | < 1 |
|  |  |  |  |
| CMP_dye5 | NO. | Target Main G | Target C (ppm) |
|  | 27 | 86 | 7.4 |
|  | 28 | 165 | < 1 |
|  | 29 | 166 | < 1 |
|  | 30 | 96 | 6.4 |
|  | 31 | 166 | < 1 |
|  | 32 | 166 | < 1 |
|  | 33 | 141 | 1.6 |
|  | 34 | 152 | < 1 |
|  | 35 | 95 | 6.4 |
|  | 36 | 166 | < 1 |
|  |  |  |  |
| CMP_dye4 | NO. | Target Main G |  |
|  | 27 | 48 | 5.1 |
|  | 28 | 166 | < 1 |
|  | 29 | 165 | < 1 |
|  | 30 | 45 | 5.4 |
|  | 31 | 165 | < 1 |
|  | 32 | 165 | < 1 |
|  | 33 | 162 | < 1 |
|  | 34 | 163 | < 1 |
|  | 35 | 22 | 8.3 |
|  | 36 | 165 | < 1 |
|  |  |  |  |
| CMP_dye3 | NO. | Target Main G |  |
|  | 27 | 133 | 9.0 |
|  | 28 | 164 | < 1 |
|  | 29 | 164 | < 1 |
|  | 30 | 149 | 4.0 |
|  | 31 | 158 | 1.4 |
|  | 32 | 156 | 2.0 |
|  | 33 | 158 | 1.3 |
|  | 34 | 151 | 3.5 |
|  | 35 | 133 | 9.0 |
|  | 36 | 164 | < 1 |
|  |  |  |  |
| CMP_dye2 | NO. | Target Main B | Target C (ppm) |
|  | 27 | 115 | 8.6 |
|  | 28 | 148 | 3.0 |
|  | 29 | 138 | 4.7 |
|  | 30 | 124 | 7.1 |
|  | 31 | 137 | 4.9 |
|  | 32 | 152 | 2.4 |
|  | 33 | 131 | 5.9 |
|  | 34 | 147 | 3.2 |
|  | 35 | 115 | 8.6 |
|  | 36 | 165 | < 1 |
|  |  |  |  |
| CMP_dye1 | NO. | Target Main B | Target C (ppm) |
|  | 27 | 140 | 8.7 |
|  | 28 | 149 | 5.3 |
|  | 29 | 145 | 6.7 |
|  | 30 | 141 | 8.0 |
|  | 31 | 145 | 6.6 |
|  | 32 | 148 | 5.8 |
|  | 33 | 147 | 6.0 |
|  | 34 | 152 | 4.5 |
|  | 35 | 140 | 8.5 |
|  | 36 | 168 | < 1 |

**Table S8**. Target ROI channel intensity and corresponding concentration for each MOF (**37–50**)

| Materials_dye X |  |  |  |
| --- | --- | --- | --- |
| MOF_dye6 | NO. | Target Main G | Target C (ppm) |
|  | 37 | 142 | 8.4 |
|  | 38 | 159 | < 1 |
|  | 39 | 155 | 2.2 |
|  | 40 | 162 | < 1 |
|  | 41 | 163 | < 1 |
|  | 42 | 148 | 5.6 |
|  | 43 | 162 | < 1 |
|  | 44 | 146 | 6.5 |
|  | 45 | 152 | 3.5 |
|  | 46 | 163 | < 1 |
|  | 47 | 163 | < 1 |
|  | 48 | 166 | < 1 |
|  | 49 | 168 | < 1 |
|  | 50 | 145 | 6.8 |
|  |  |  |  |
| MOF_dye5 | NO. | Target Main G | Target C (ppm) |
|  | 37 | 68 | 9.4 |
|  | 38 | 160 | < 1 |
|  | 39 | 85 | 7.6 |
|  | 40 | 145 | 1.2 |
|  | 41 | 162 | < 1 |
|  | 42 | 81 | 8.0 |
|  | 43 | 155 | < 1 |
|  | 44 | 71 | 9.1 |
|  | 45 | 67 | 9.4 |
|  | 46 | 156 | < 1 |
|  | 47 | 110 | 4.9 |
|  | 48 | 160 | < 1 |
|  | 49 | 166 | < 1 |
|  | 50 | 75 | 8.6 |
|  |  |  |  |
| MOF_dye4 | NO. | Target Main G | Target C (ppm) |
|  | 37 | 25 | 7.8 |
|  | 38 | 159 | < 1 |
|  | 39 | 34 | 6.5 |
|  | 40 | 159 | < 1 |
|  | 41 | 162 | < 1 |
|  | 42 | 34 | 6.5 |
|  | 43 | 157 | < 1 |
|  | 44 | 42 | 5.6 |
|  | 45 | 97 | 2.2 |
|  | 46 | 163 | < 1 |
|  | 47 | 44 | 5.4 |
|  | 48 | 167 | < 1 |
|  | 49 | 166 | < 1 |
|  | 50 | 25 | 7.8 |
|  |  |  |  |
| MOF_dye3 | NO. | Target Main G | Target C (ppm) |
|  | 37 | 135 | 8.4 |
|  | 38 | 135 | 8.3 |
|  | 39 | 166 | < 1 |
|  | 40 | 162 | < 1 |
|  | 41 | 162 | < 1 |
|  | 42 | 162 | < 1 |
|  | 43 | 157 | 1.6 |
|  | 44 | 131 | 9.7 |
|  | 45 | 133 | 8.9 |
|  | 46 | 136 | 8.0 |
|  | 47 | 128 | 10.3 |
|  | 48 | 167 | < 1 |
|  | 49 | 167 | < 1 |
|  | 50 | 146 | 5.1 |
|  |  |  |  |
| MOF_dye2 | NO. | Target Main B | Target C (ppm) |
|  | 37 | 163 | < 1 |
|  | 38 | 126 | 6.7 |
|  | 39 | 165 | < 1 |
|  | 40 | 161 | < 1 |
|  | 41 | 161 | < 1 |
|  | 42 | 163 | < 1 |
|  | 43 | 158 | 1.2 |
|  | 44 | 120 | 7.8 |
|  | 45 | 117 | 8.4 |
|  | 46 | 123 | 7.2 |
|  | 47 | 142 | 4.1 |
|  | 48 | 166 | < 1 |
|  | 49 | 166 | < 1 |
|  | 50 | 166 | < 1 |
|  |  |  |  |
| MOF_dye1 | NO. | Target Main B | Target C (ppm) |
|  | 37 | 160 | 1.6 |
|  | 38 | 143 | 7.7 |
|  | 39 | 166 | < 1 |
|  | 40 | 161 | 1.4 |
|  | 41 | 140 | 8.8 |
|  | 42 | 162 | < 1 |
|  | 43 | 163 | < 1 |
|  | 44 | 142 | 8.0 |
|  | 45 | 137 | 9.6 |
|  | 46 | 143 | 7.7 |
|  | 47 | 137 | 9.7 |
|  | 48 | 167 | < 1 |
|  | 49 | 166 | < 1 |
|  | 50 | 166 | < 1 |

**Reproducibility test**

To address the reviewer’s concern regarding reproducibility, we selected seven representative materials from the library (**30, 47, 26, 31, 40, 41, 49**) and conducted two independent replicate tests using freshly prepared dye solutions and the same samples as in the original screening. The results from all three rounds of testing (initial test plus two replicates) are shown in Figure S30. These materials exhibited between 0 and 6 dye responses across the array. Based on both dye adsorption and colorimetric analysis, five of the seven materials (**26, 31, 40, 41, 49**) were consistently classified as porous, each showing between 2 and 6 hits. By contrast, the amine cages CMP2-a (**30**) and Ni-PYMS (**47**) were correctly classified as non-porous. The target ROI channel intensities and corresponding dye concentrations for the seven materials examined in the reproducibility test have been added to the Experimental Results section in the SI (Tables S8 and S9). Discrepancies observed included the absence of a hit for dye 6 in Ni-PYMS (Figure S30 (b), (c)), which had previously been detected (Figure S30 (a)). We consider this outcome reasonable, as non-porous materials are expected to exhibit negligible dye adsorption. Another minor variation was the observation of moderate fading for some dyes, such as dye 4 in **30** and dye 5 in **47**, which did not reach the threshold for a hit (Figure S30 (b) and (c)). Overall, although some variation in fading intensity may occur across the assay, these minor discrepancies did not impact classification accuracy. In summary, the multichannel approach allows consistent classification results to be obtained across the three independent tests, highlighting the robustness and reliability of our multichannel method.


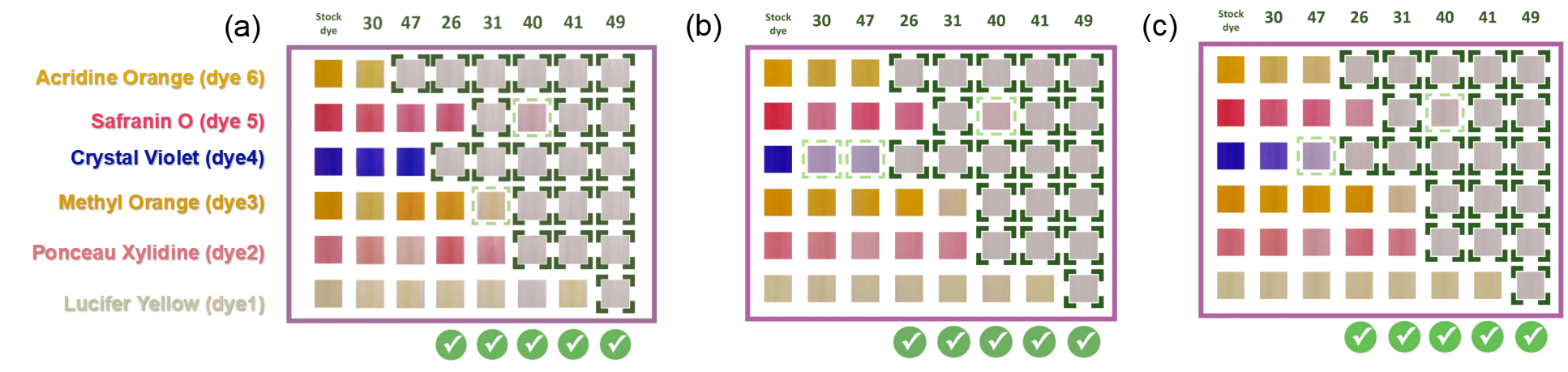


**Figure S30** Colorimetric maps for seven materials (**30，47，26，31，40，41，49**): (a) initial test results from the high-throughput screening; (b) results from the second replicate; and (c) results from the third replicate. The six stock dyes, listed from top to bottom, are Acridine Orange (dye 6), Safranin O (dye 5), Crystal Violet (dye 4), Methyl Orange (dye 3), Ponceau Xylidine (dye 2), and Lucifer Yellow (dye 1).


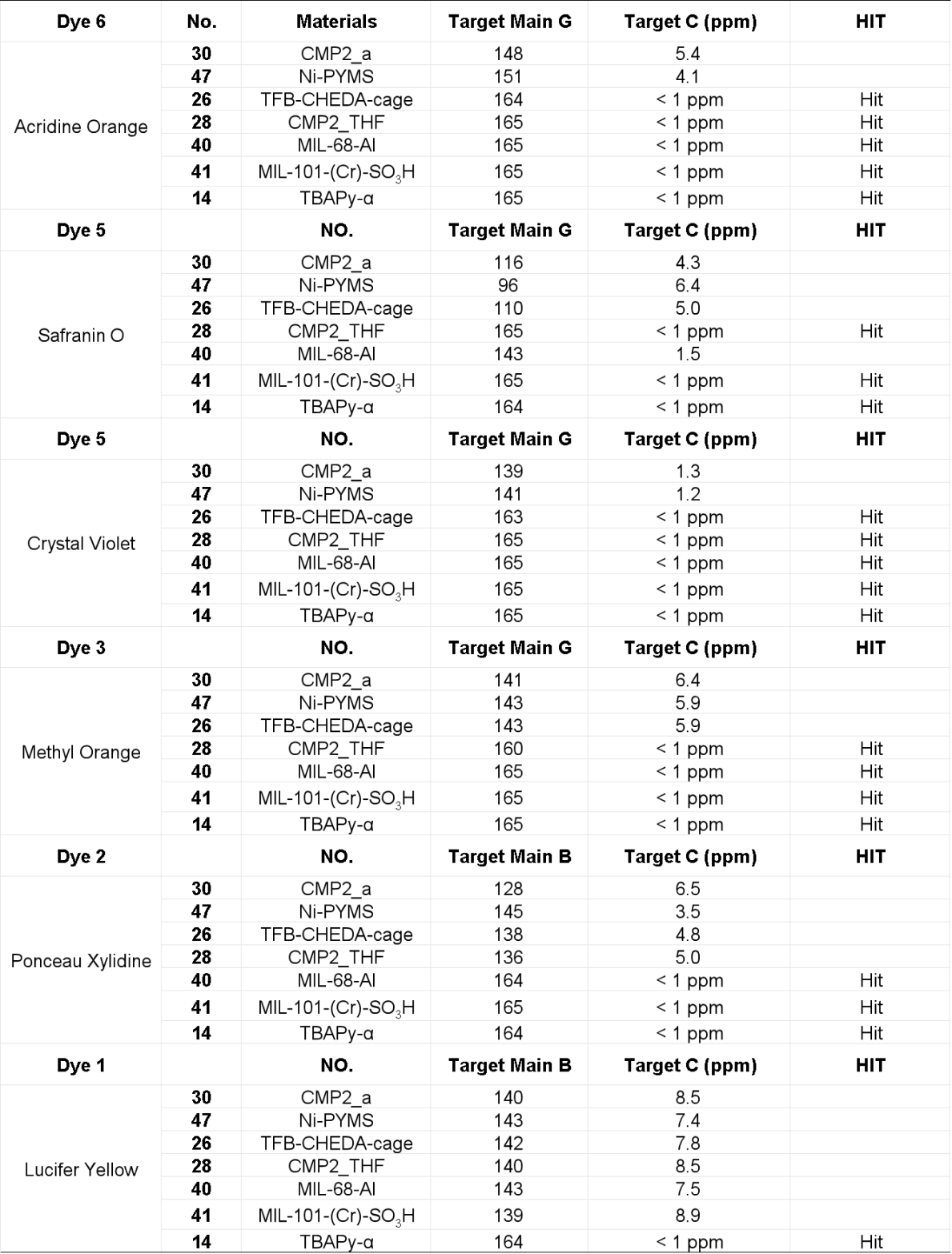


**Table S9.** Target ROI channel intensity and corresponding concentration for seven materials for 2^nd^ reproducibility test


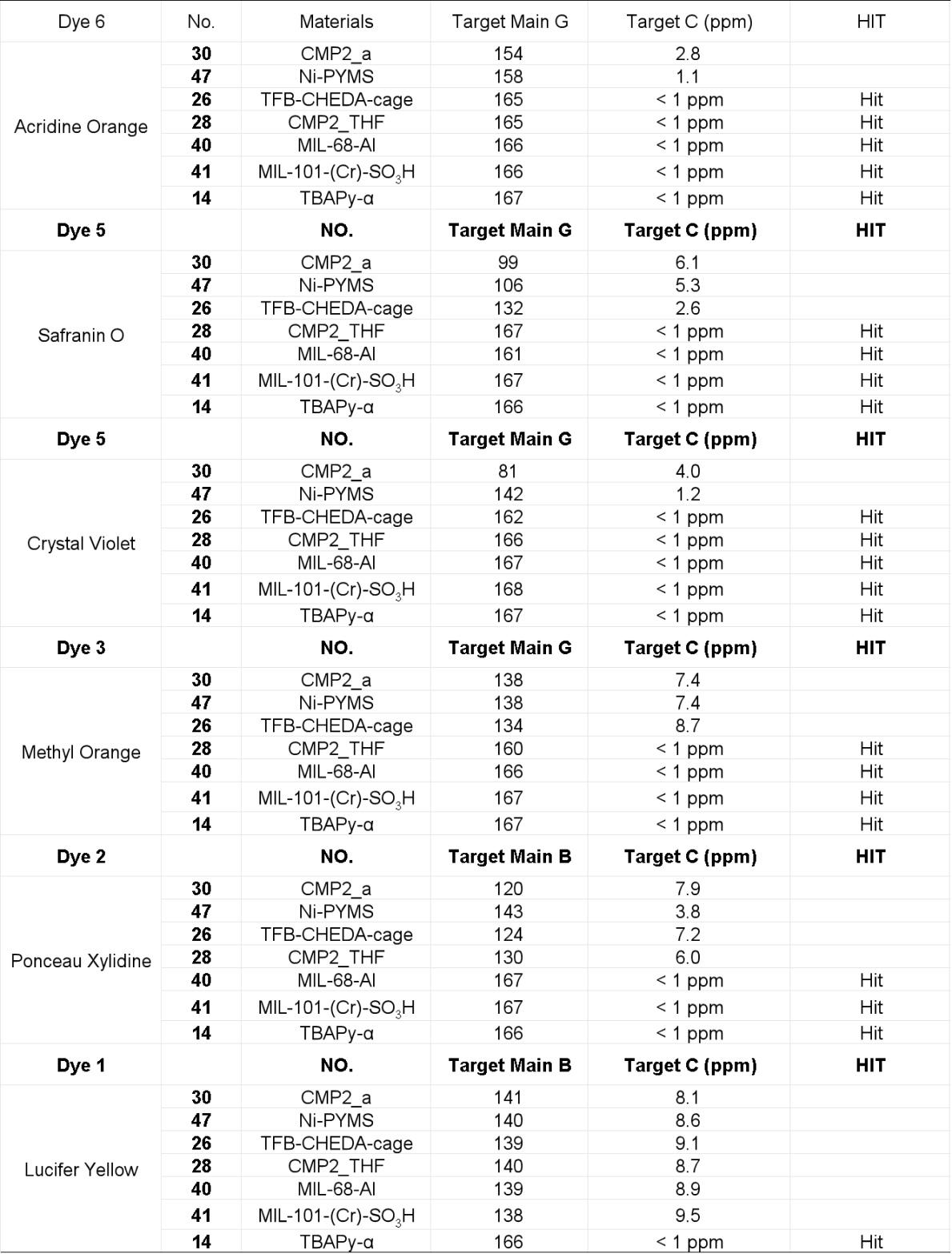


**Table S10.** Target ROI channel intensity and corresponding concentration for seven materials for 3^rd^ reproducibility test


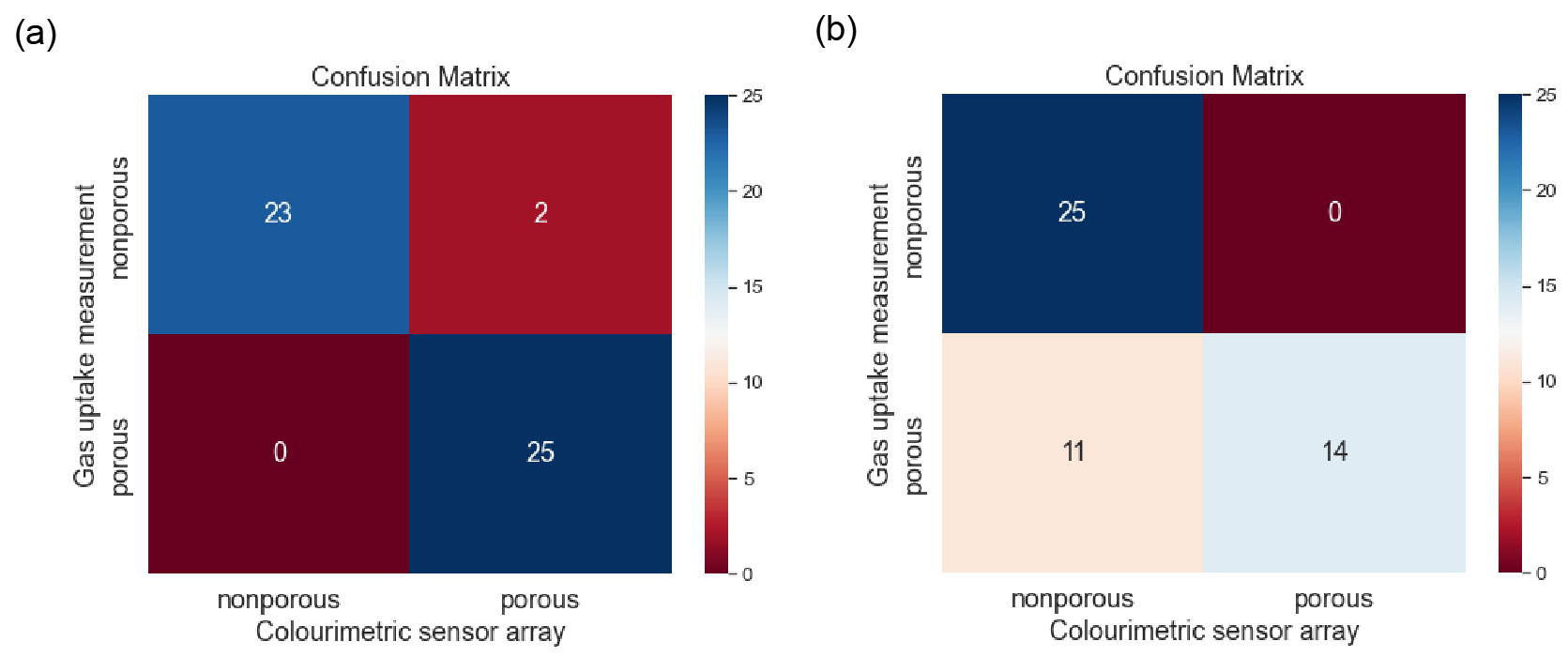


**Figure S31**. Confusion matrix for porosity classification of the 50 materials tested using different thresholds (a) assigning porosity to a single hit (or more); (b) assigning a threshold of at least three hits. The classification accuracy dropped to 96% in the first case (two false positives outputs) and to 78 % in the second case (eleven false negatives).


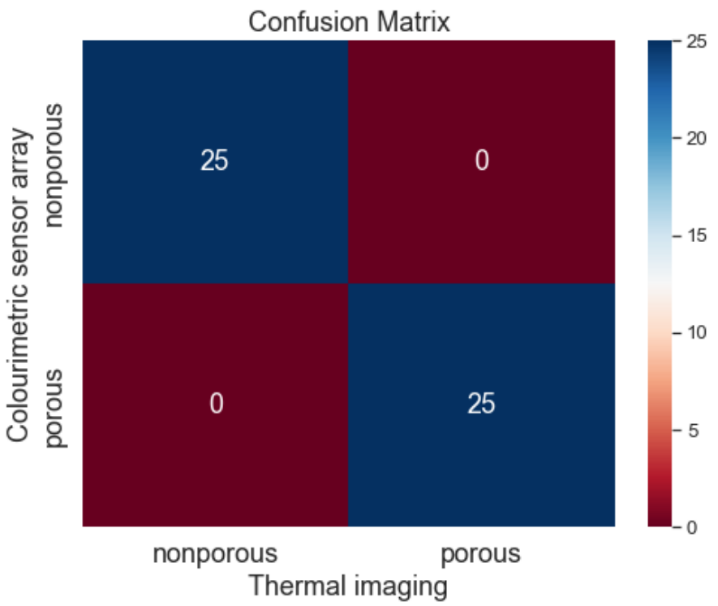


**Figure S32**. Confusion matrix for porosity classification of the 50 materials tested incorporating the ‘Moderate fade’ results into the threshold and defining these points to be a ‘hit’, too. With this adjustment, the classification accuracy improves to 100% by correctly identifying one additional porous MOF, MOF-801, according to our definition of porosity corresponding to >0.1 cm^3^ g^-1^ pore volume as measured by N_2_ sorption at 77 K.


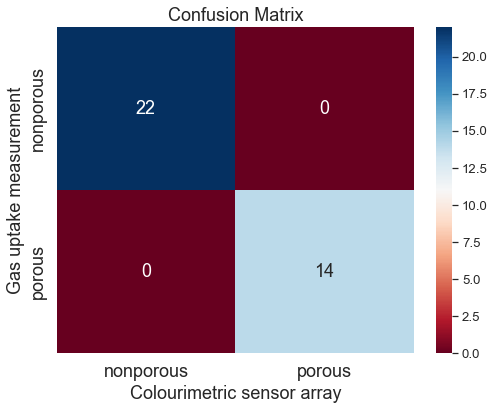


**Figure S33**. Confusion matrix for porosity classification of 36 materials including small molecules, cages, and CMPs (**1**–**36**) showing the correlation with gas uptake measurements with 3 dye channels. Our goal is to identify porous materials that exhibit at least two hits. Using a reduced subarray of only three dyes (crystal violet, safranin O, and acridine orange), we can still achieve a classification accuracy of 100% with no false negatives


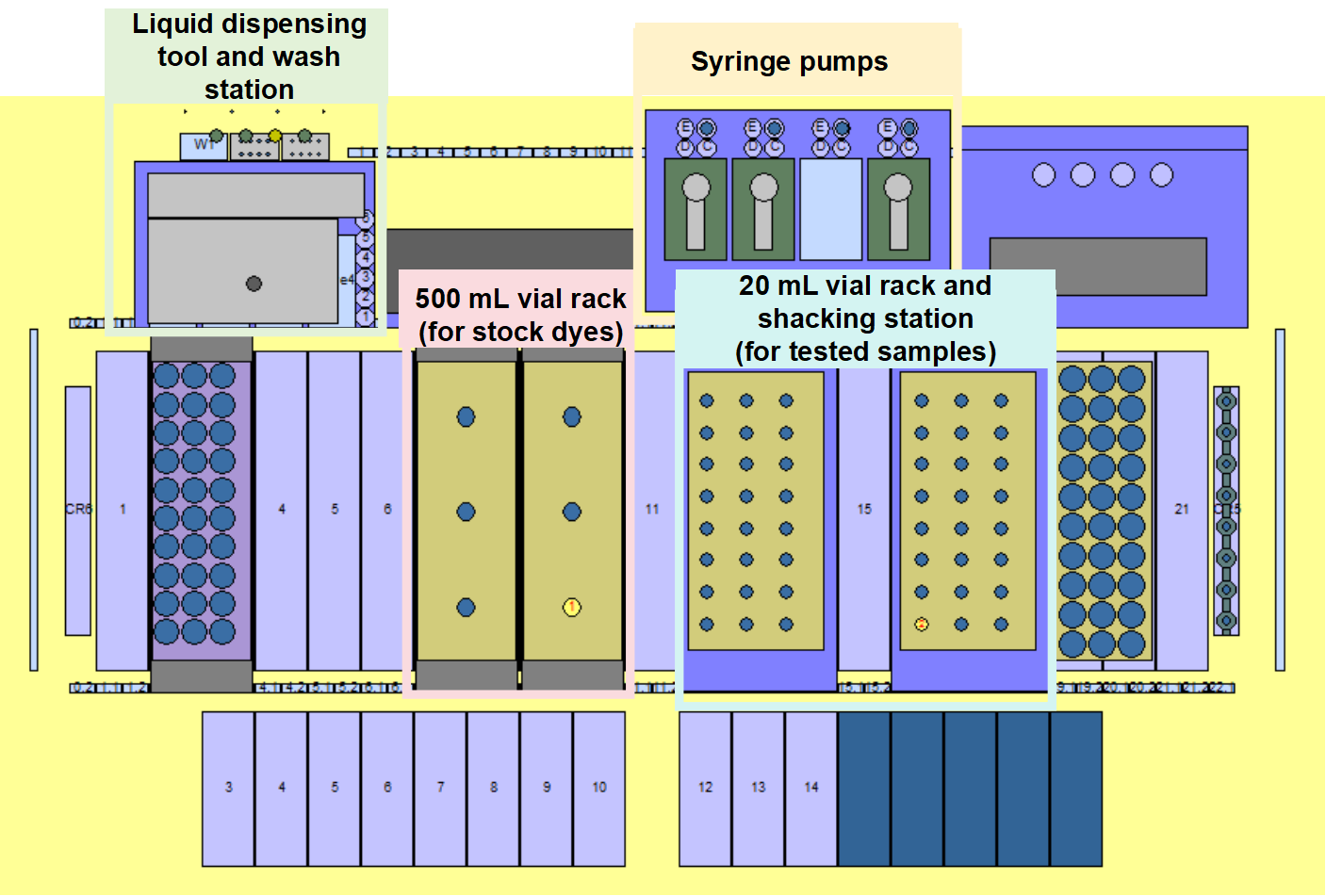


**Figure S34**: Chemspeed SWING robotic platform configuration for semi-automated porosity screening. Liquid handling was carried out using the four needle overhead dispensing tool with 4 syringe pumps. Dye solutions were transferred from each 500 mL stock vial rack to a 20 mL vial rack containing the test samples. To avoid cross-contamination, the dispensing needles were rinsed with water after each dye transfers. Liquid handling and shacking were performed at RT in a closed system.


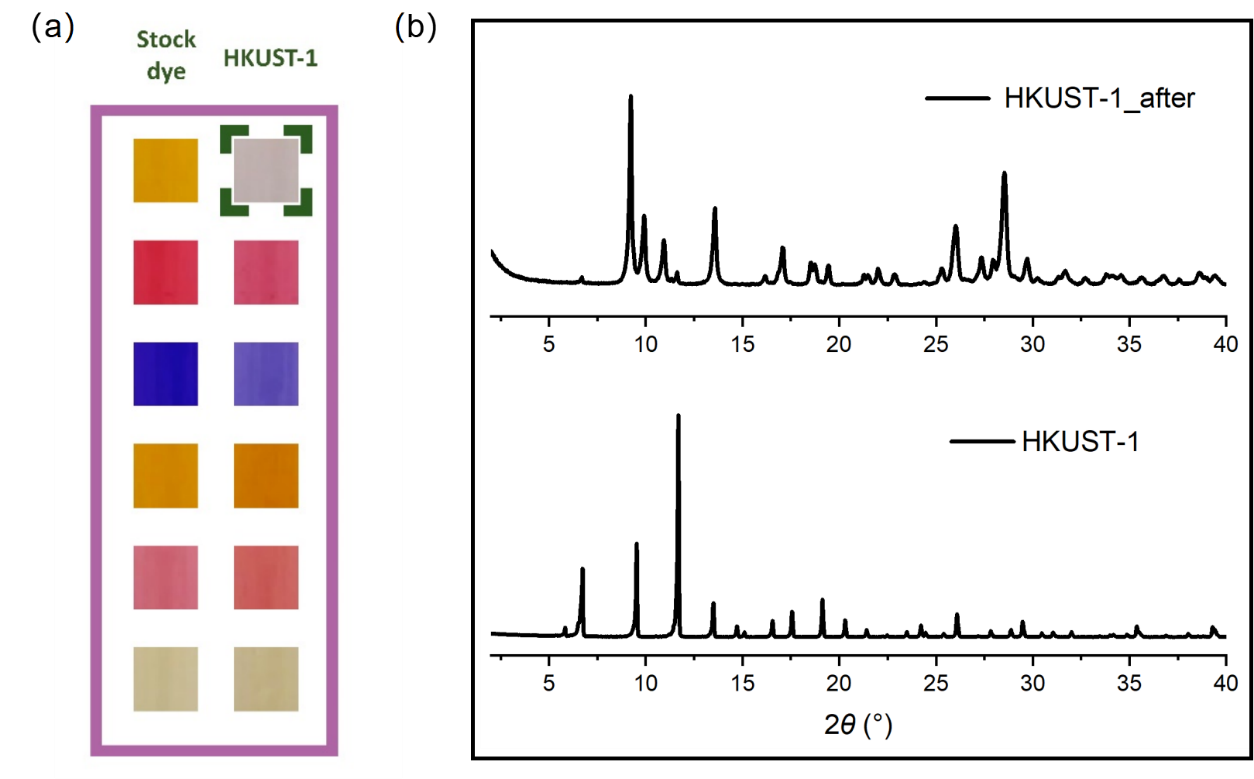


**Figure S35** (a) Colorimetric maps for HKUST-1 and stock dye. The six stock dyes, listed from top to bottom, are Acridine Orange (dye 6), Safranin O (dye 5), Crystal Violet (dye 4), Methyl Orange (dye 3), Ponceau Xylidine (dye 2), and Lucifer Yellow (dye 1). (b) PXRD patterns of HKUST-1 before dye adsorption (bottom) and after dye adsorption (top). Due to small sample amount used for each dye solution, we collected materials from all six dye solutions and combined them. Following filtration and drying under vacuum at 100 °C, a PXRD pattern of the recovered material was recorded.


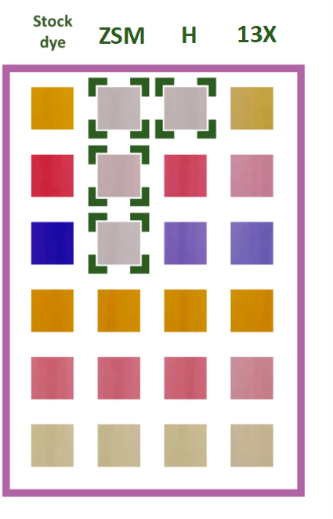
In order to further verify the applicability of the method for water absorbents, we have tested three microporous zeolites: ZSM-5^16^ (0.53 nm pore size), H-USY^17^ (0.74 nm), and 13X^18^ (1 nm pore size) using the dye array. As shown in Figure S36, ZSM-5 had three hits, H-USY had one hit, and 13X had no hits. We attribute the lack of hits for 13X to its strong water adsorption capacity, which leads to pore saturation and limits dye uptake, consistent with its known behavior as a molecular sieve^19^. These results confirm that while specific highly hydrophilic zeolites (*e.g.*, 13X) may indeed present challenges for the dye adsorption method due to strong water adsorption, other zeolites (*e.g.*, ZSM-5, H-USY) can still be effectively classified using this method.

**Figure S36** Colorimetric maps for three representative porous zeolites ZSM-5, H-SUSY, and 13X. The six stock dyes, listed from top to bottom, are Acridine Orange (dye 6), Safranin O (dye 5), Crystal Violet (dye 4), Methyl Orange (dye 3), Ponceau Xylidine (dye 2), and Lucifer Yellow (dye 1). The target ROI channel intensities and corresponding dye concentrations of three materials have been added to Table S11.


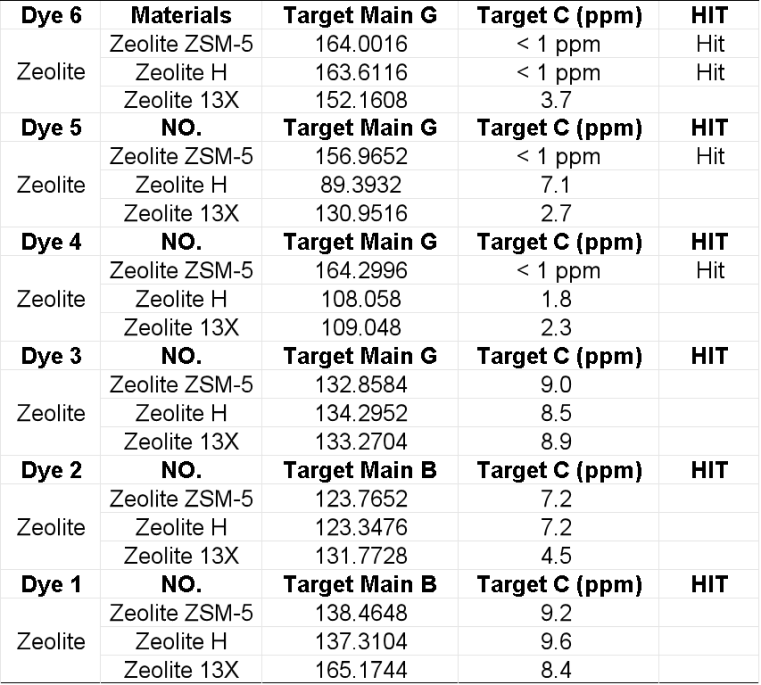


**Table S11.** Target ROI channel intensity and corresponding concentration for seven materials for 3^rd^ reproducibility test

# **Gas Adsorption Summary**

**Gas uptake measurement**

**
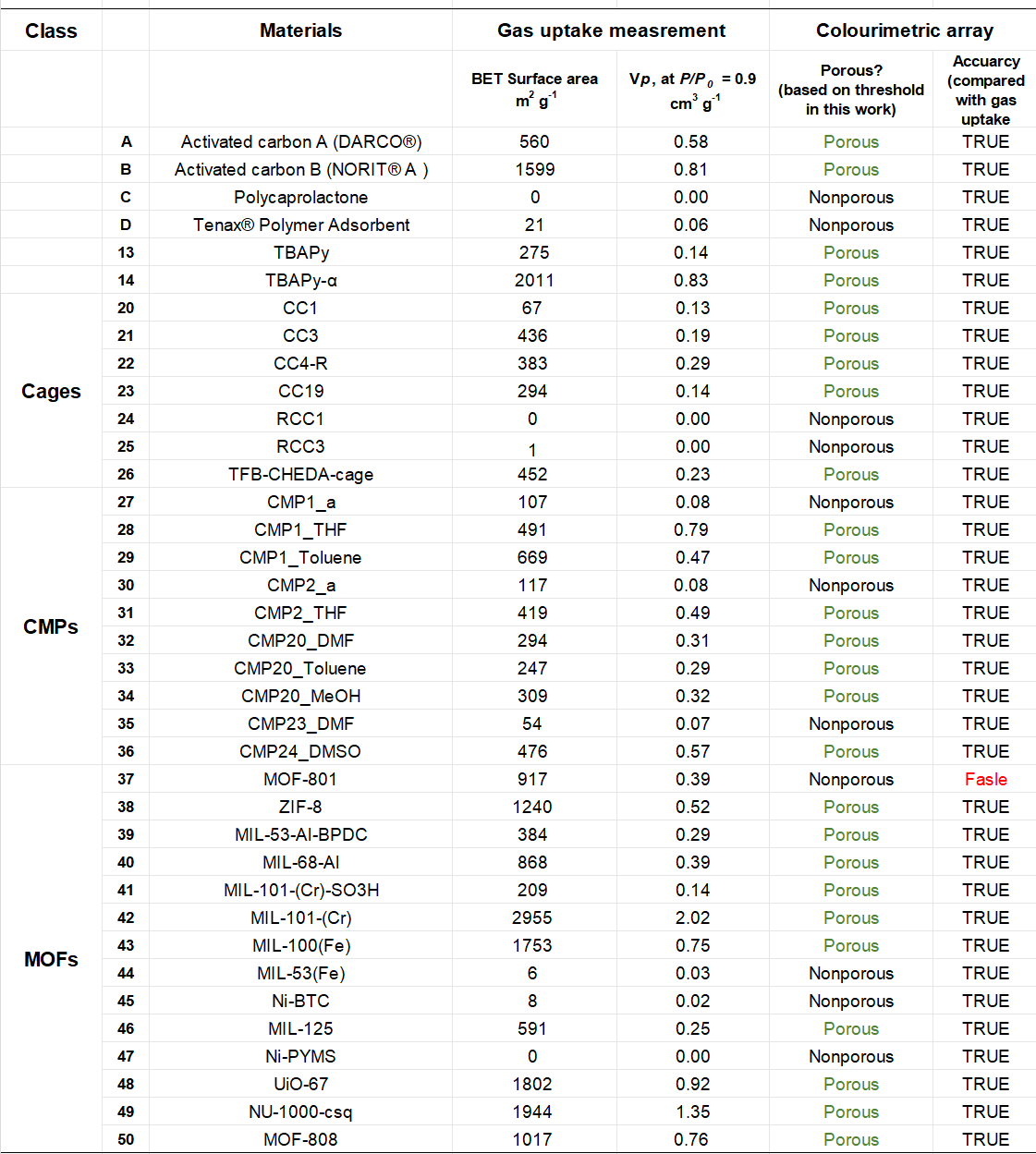
**Gases of the following purities were used: hydrogen (99.9995% - BOC gases) and carbon dioxide (SCF grade – BOC gases). Total pore volume and surface areas were measured by nitrogen adsorption and desorption at 77.3 K using a Micromeritics ASAP 2020 volumetric adsorption analyzer. Samples were degassed offline at their activation temperature for 15 h under vacuum (10^-5^ bar) before analysis. Carbon dioxide isotherms were measured at 298 K using a Micromeritics 2420 volumetric adsorption analyzer using the same degassing procedure.

**Table S12**. Comparison of porosity classification accuracy between colorimetric array and conventional gas uptake data

**
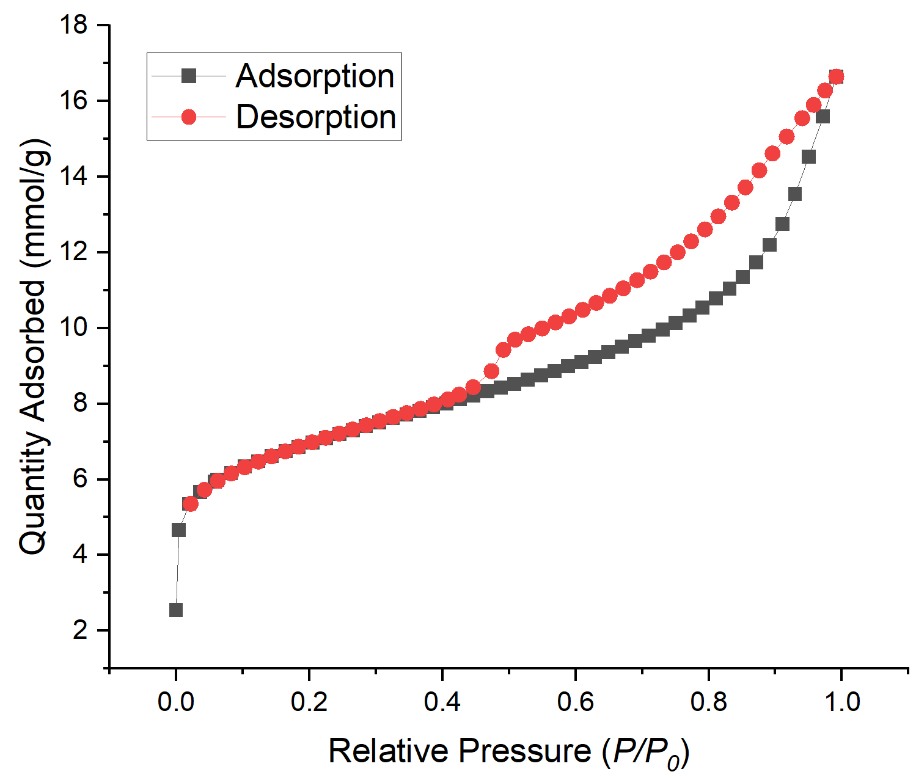
**

**Figure S37** Nitrogen adsorption isotherm of **A**_Activated carbon (DARCO®)

**
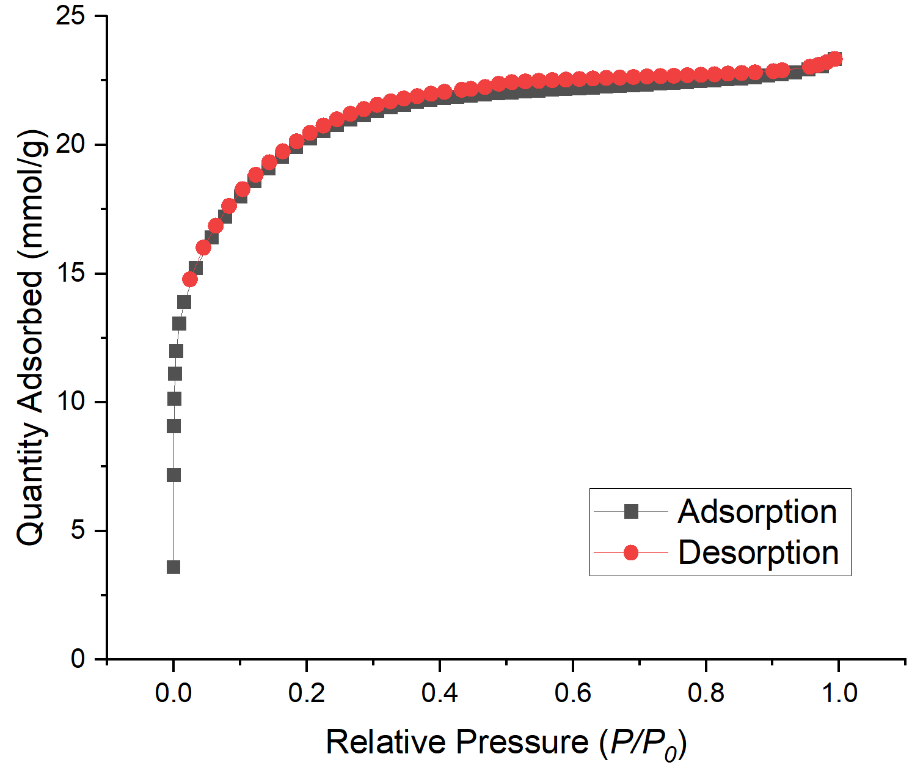

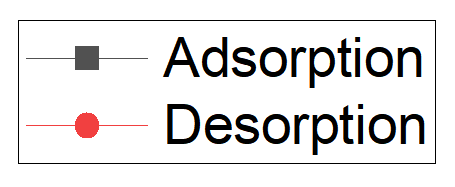
**

**Figure S38** Nitrogen adsorption isotherm of **B**_ Activated carbon (NORIT® A)


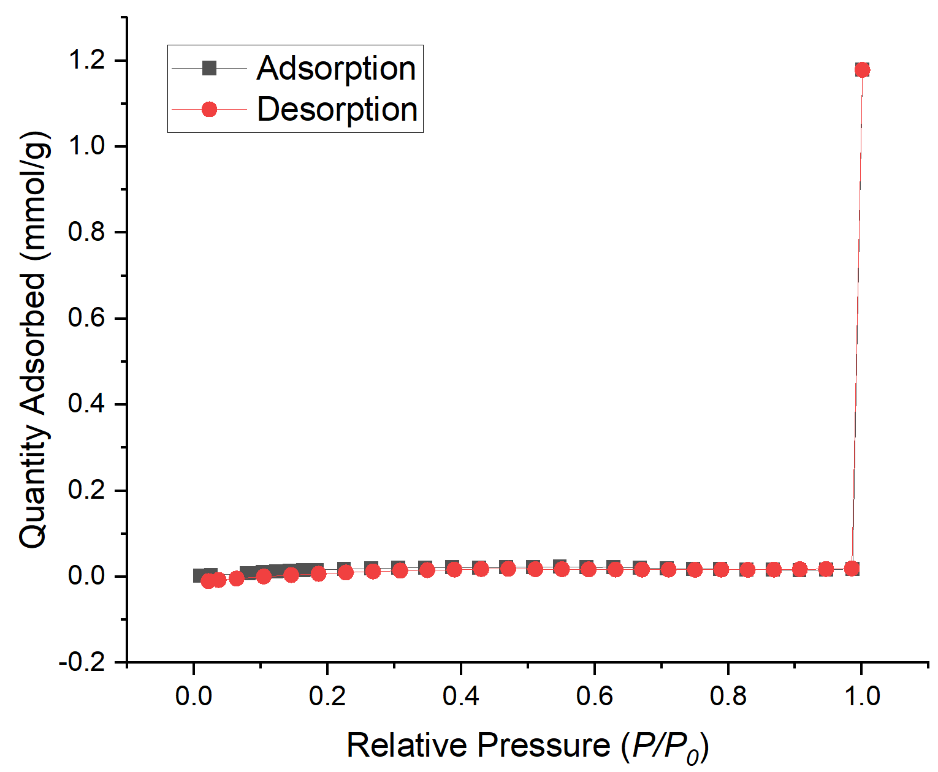


**Figure S39** Nitrogen adsorption isotherm of **C**_ Polycaprolactone


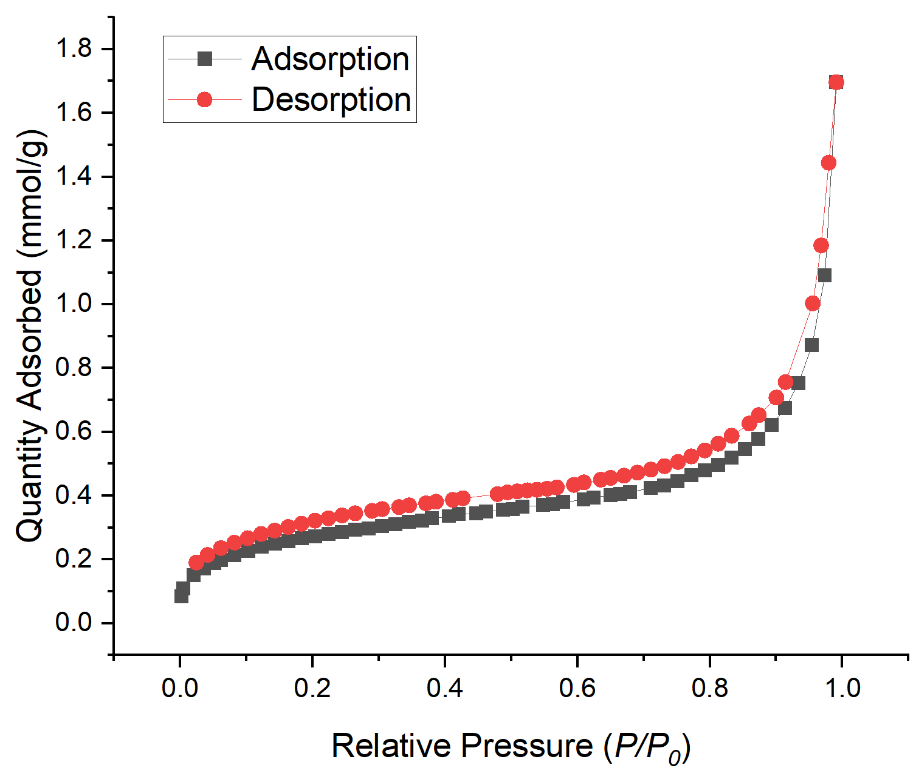


**Figure S40** Nitrogen adsorption isotherm of **D**_Tenax® Polymer Adsorbent


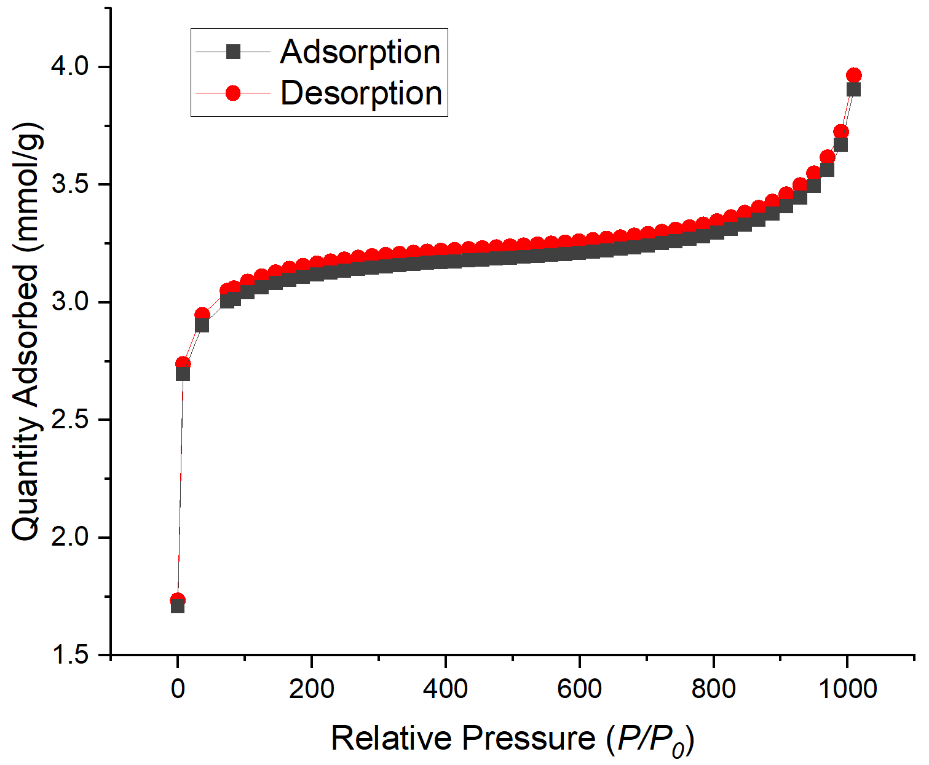


**Figure S41** Nitrogen adsorption isotherm of **13_TBAP-β**


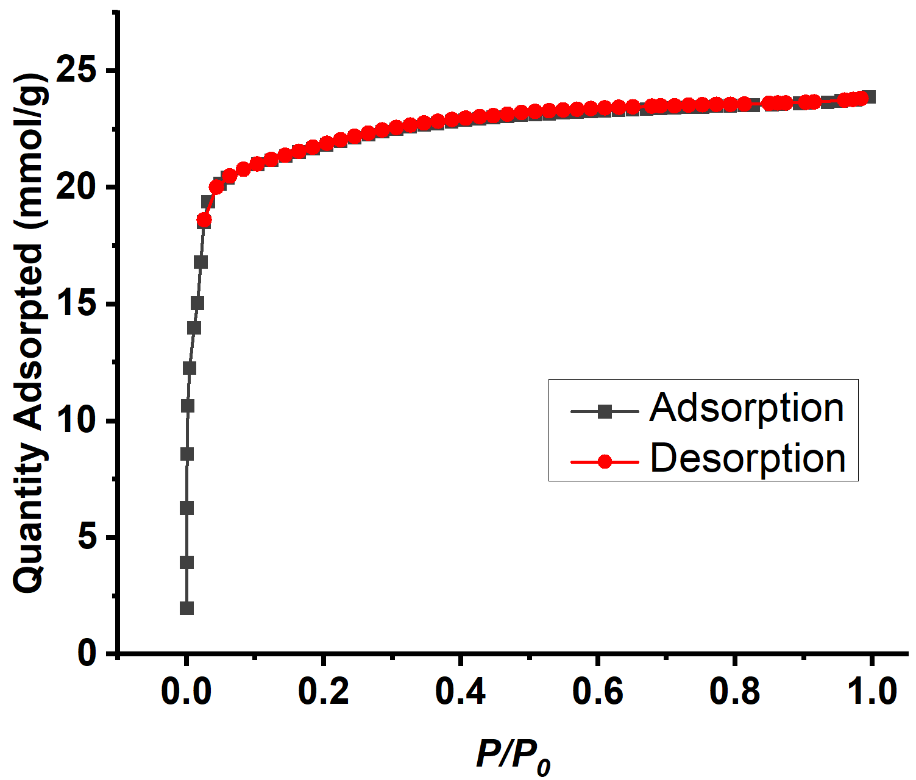


**Figure S42** Nitrogen adsorption isotherm of **14_TBAPy-α**


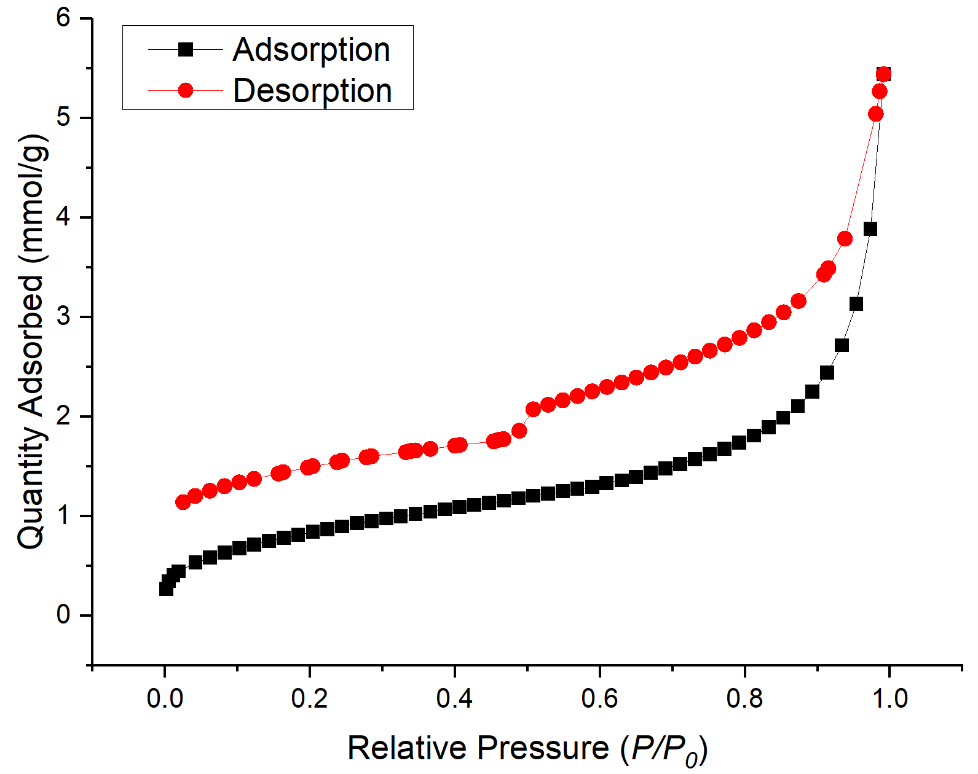


**Figure S43** Nitrogen adsorption isotherm of **20_CC1**


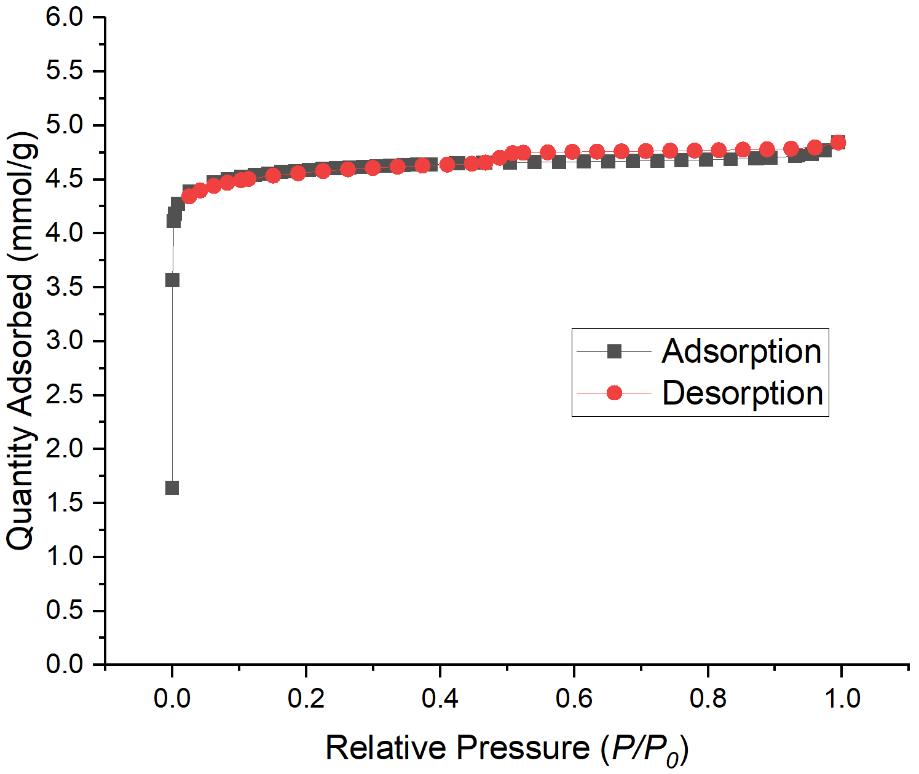


**Figure S44** Nitrogen adsorption isotherm of **21_CC3**


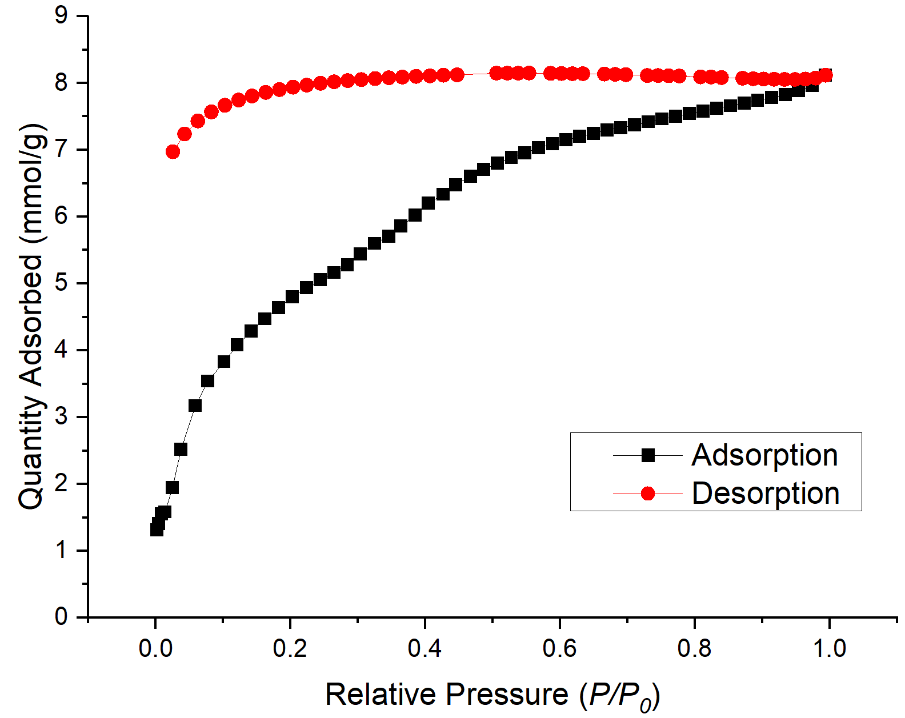

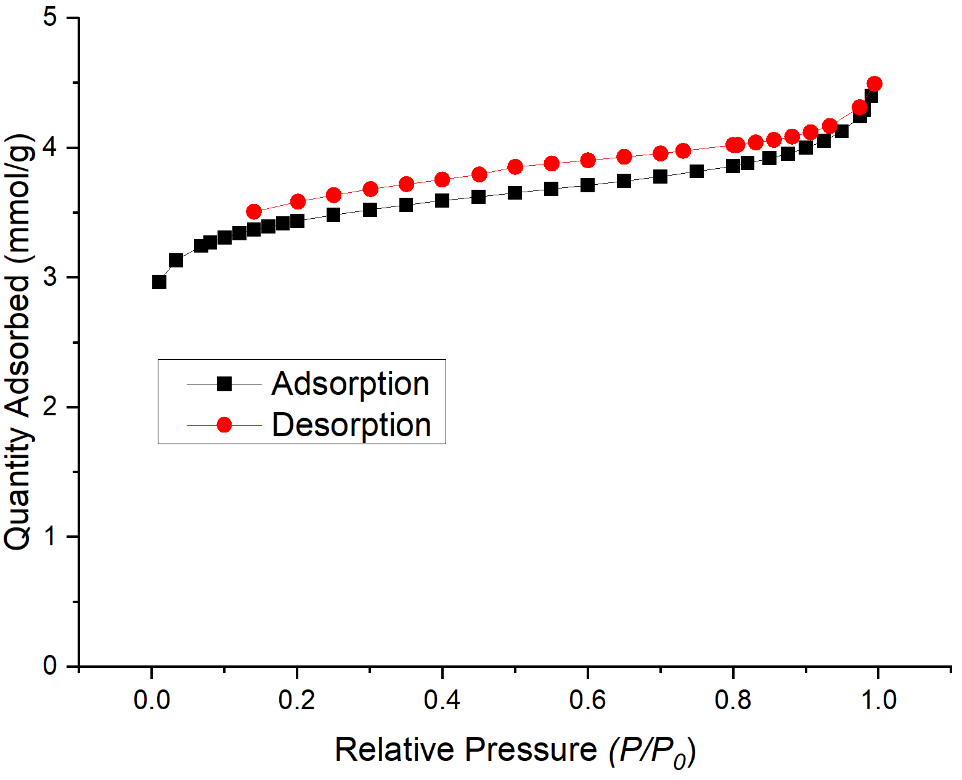


**Figure S46** Nitrogen adsorption isotherm of **23_CC19**

**Figure S45** Nitrogen adsorption isotherm of **22_CC4-R**


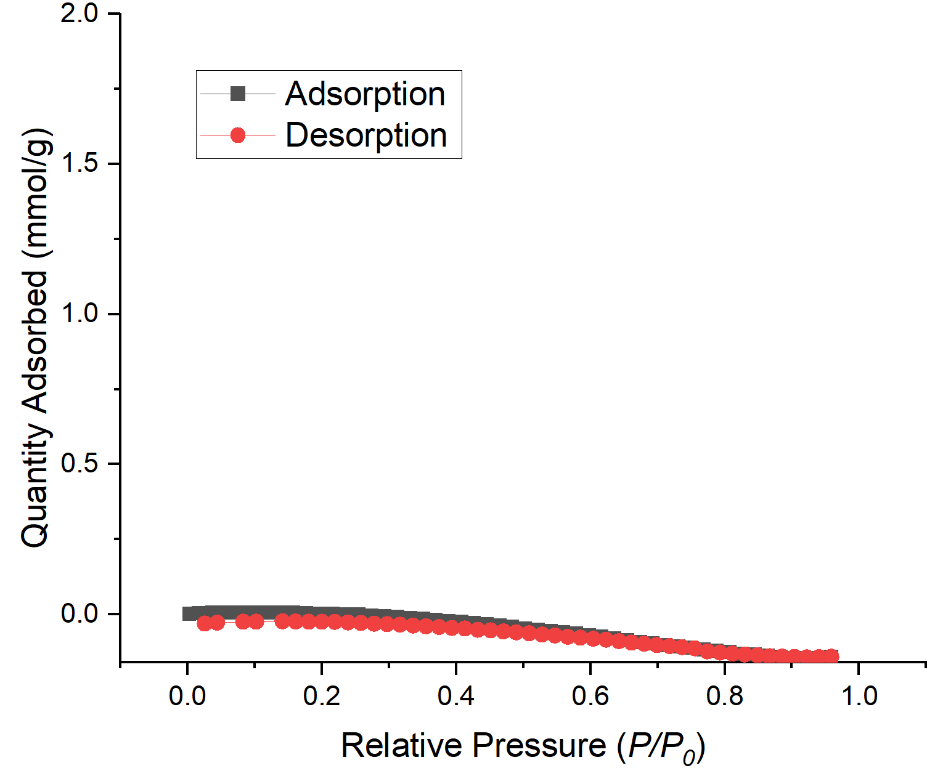


**Figure S47** Nitrogen adsorption isotherm of **24.** As **RCC1** has been confirmed to be nonporous in previous research, its nitrogen adsorption isotherm typically remains close to 0 mmol/g.


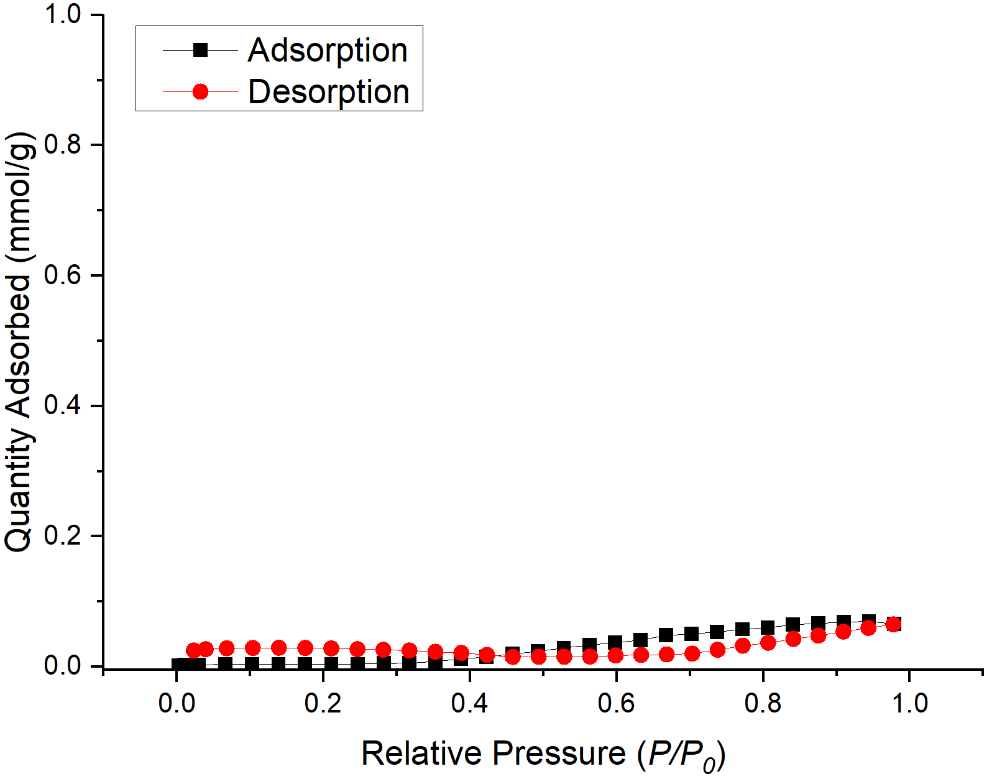


**Figure S48** Nitrogen adsorption isotherm of **25.** As **RCC3** has been confirmed to be nonporous in previous research, its nitrogen adsorption isotherm typically remains close to 0 mmol/g.


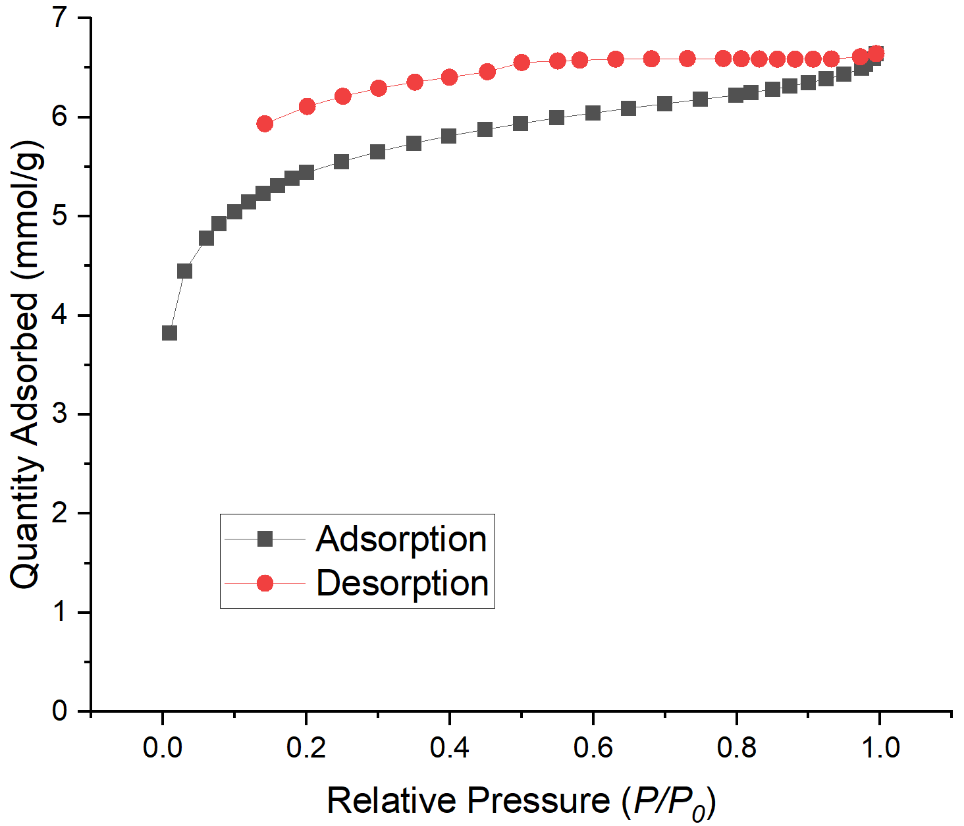


**Figure S49** Nitrogen adsorption isotherm of **26_TFB-CHEDA-cage**


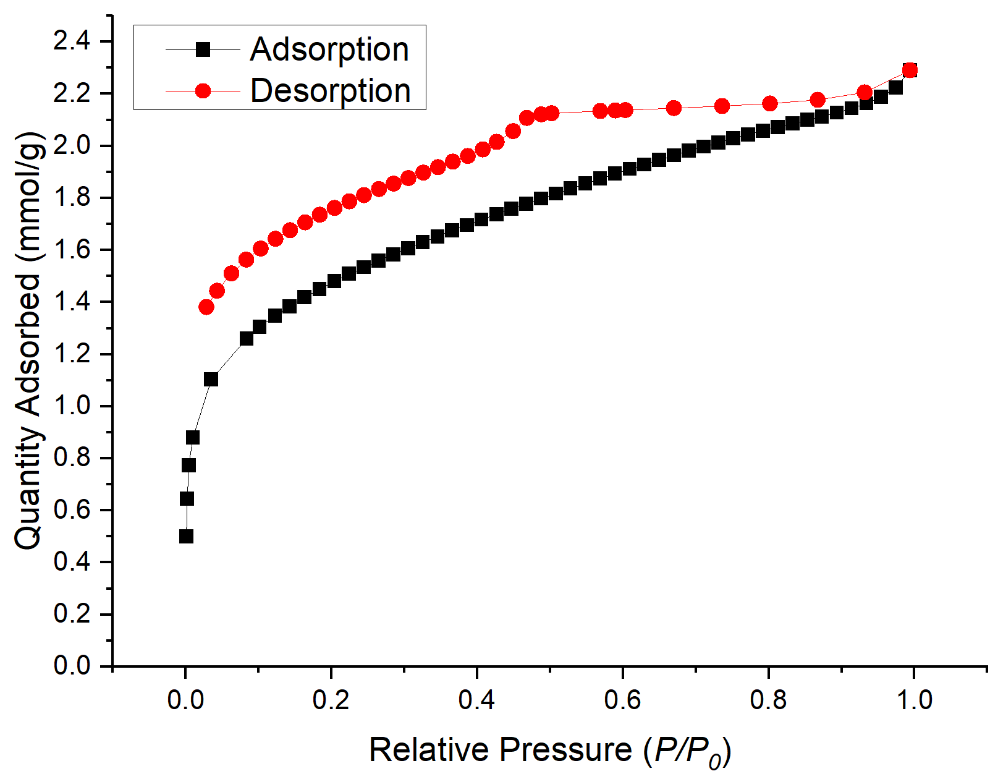


**Figure S50** Nitrogen adsorption isotherm of **27_CMP1_a**


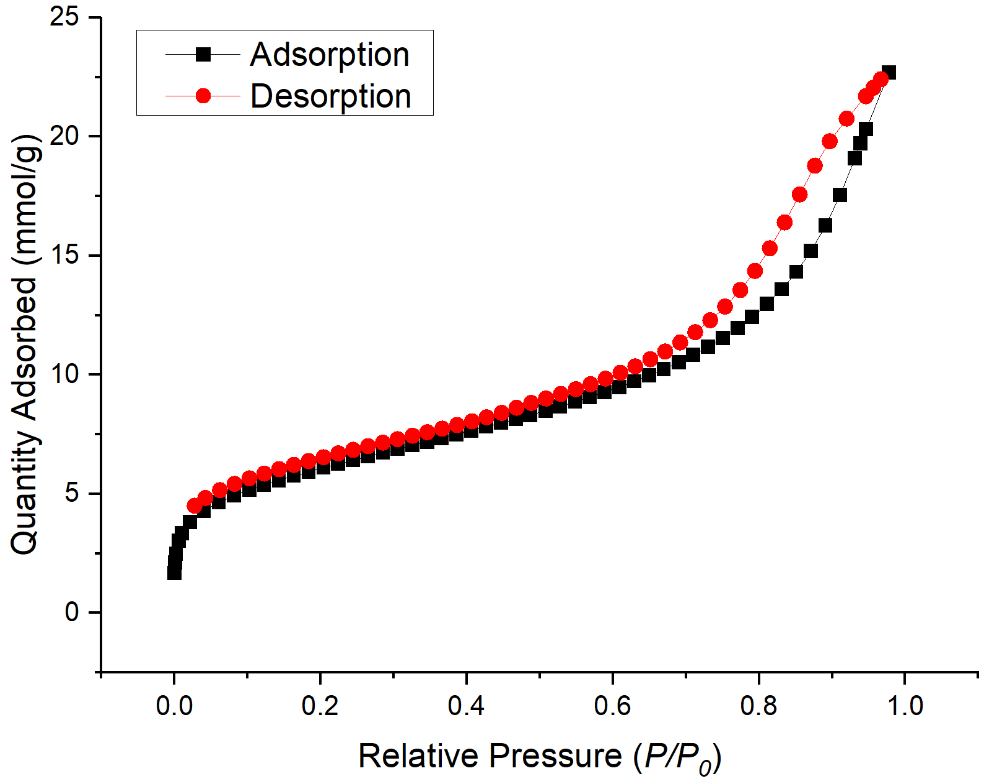


**Figure S51** Nitrogen adsorption isotherm of **28_CMP1_THF**


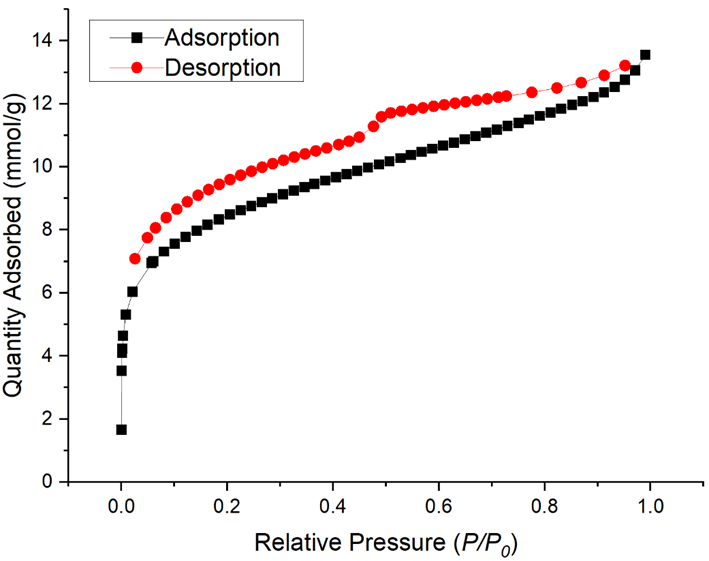


**Figure S52** Nitrogen adsorption isotherm of **29_CMP1_Toluene**


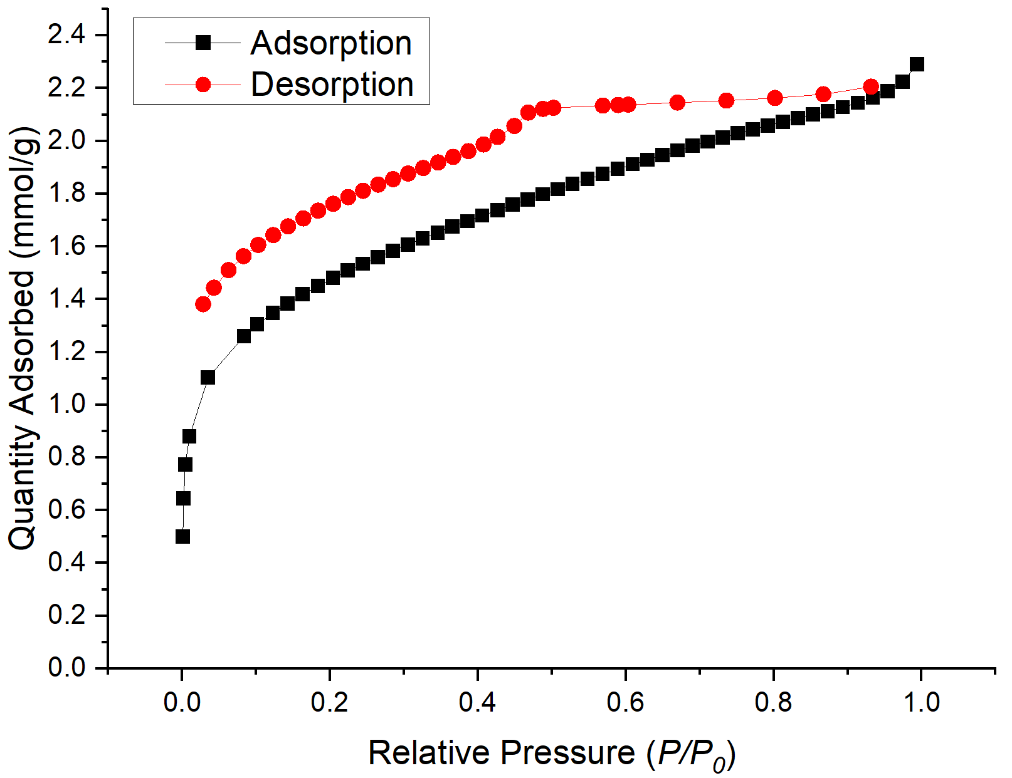


**Figure S53** Nitrogen adsorption isotherm of **30_CMP2_a**


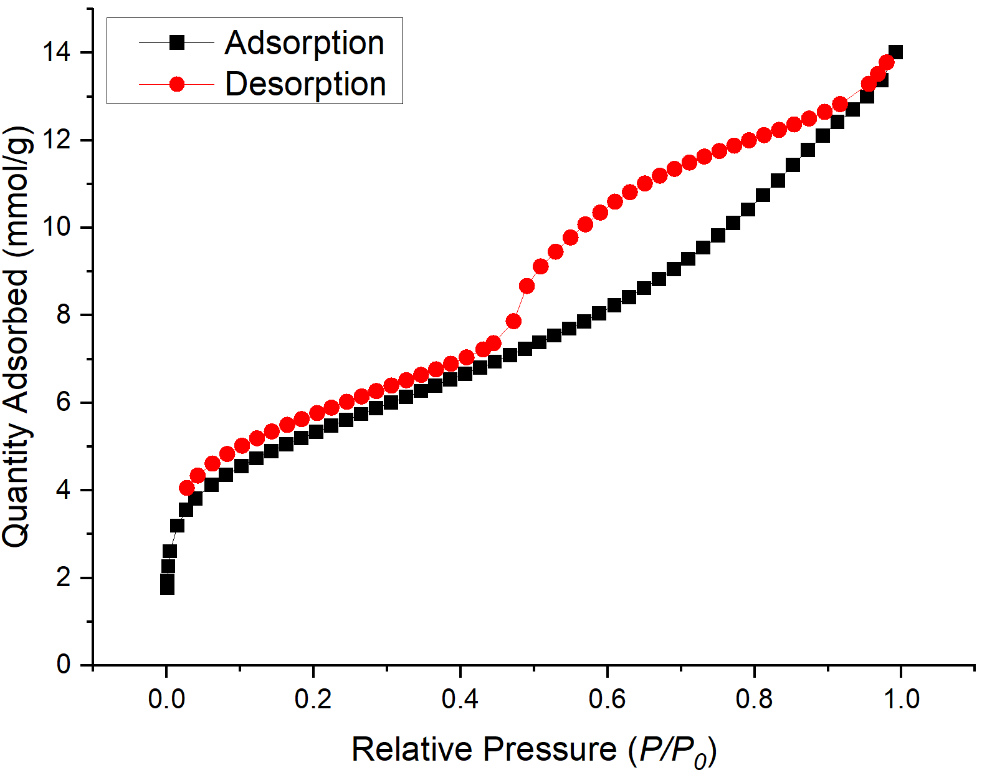


**Figure S54** Nitrogen adsorption isotherm of **31_CMP2_THF**


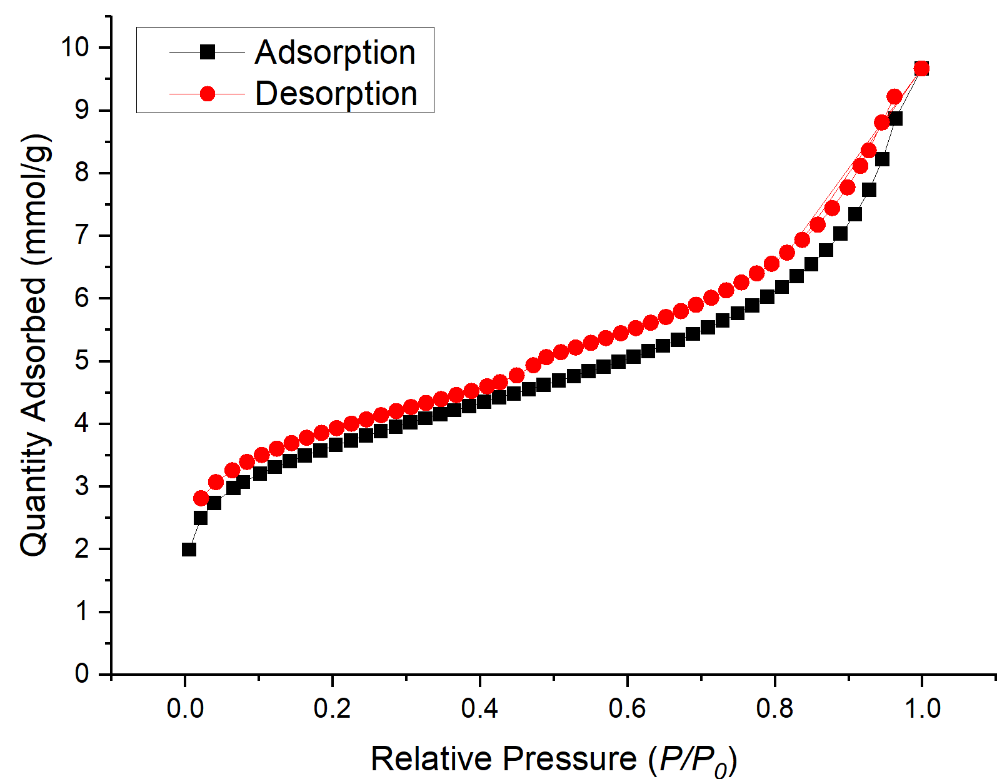


**Figure S55** Nitrogen adsorption isotherm of **32_CMP20_DMF**


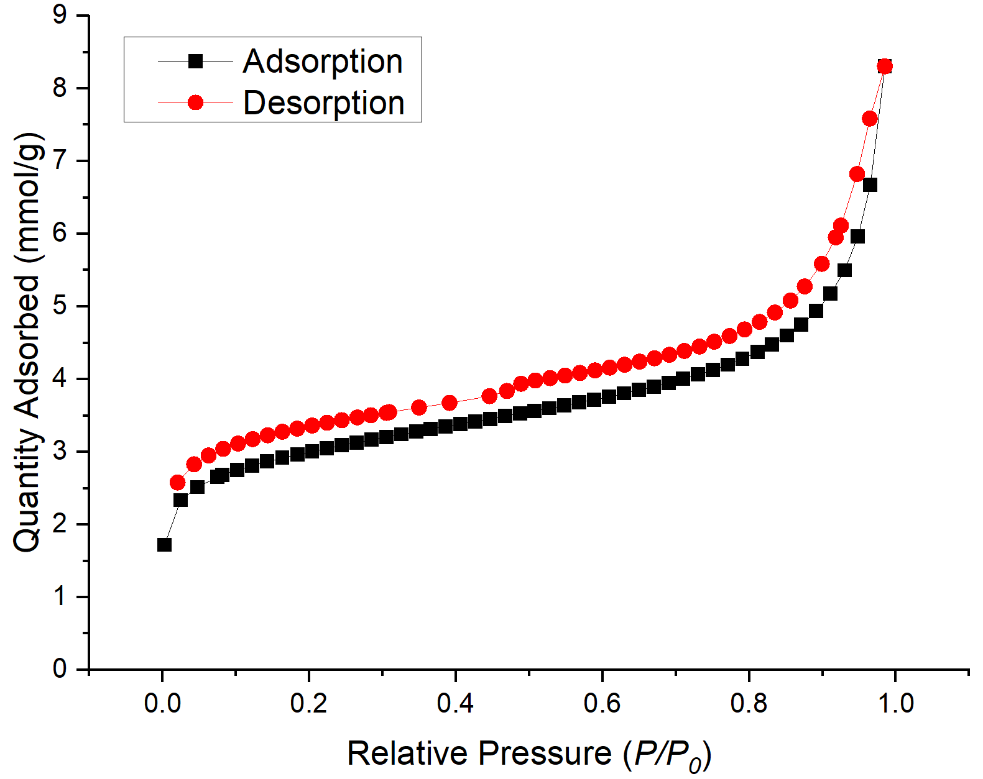


**Figure S56** Nitrogen adsorption isotherm of **33_CMP20_Toluene**


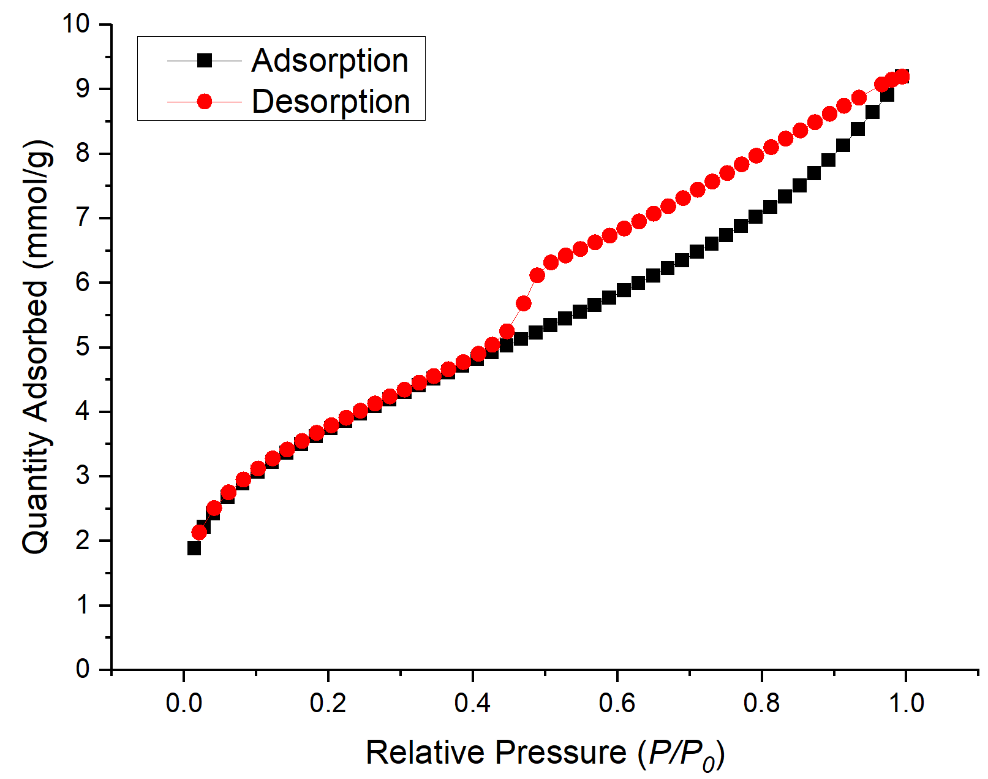


**Figure S57** Nitrogen adsorption isotherm of **34_CMP20_MeOH**


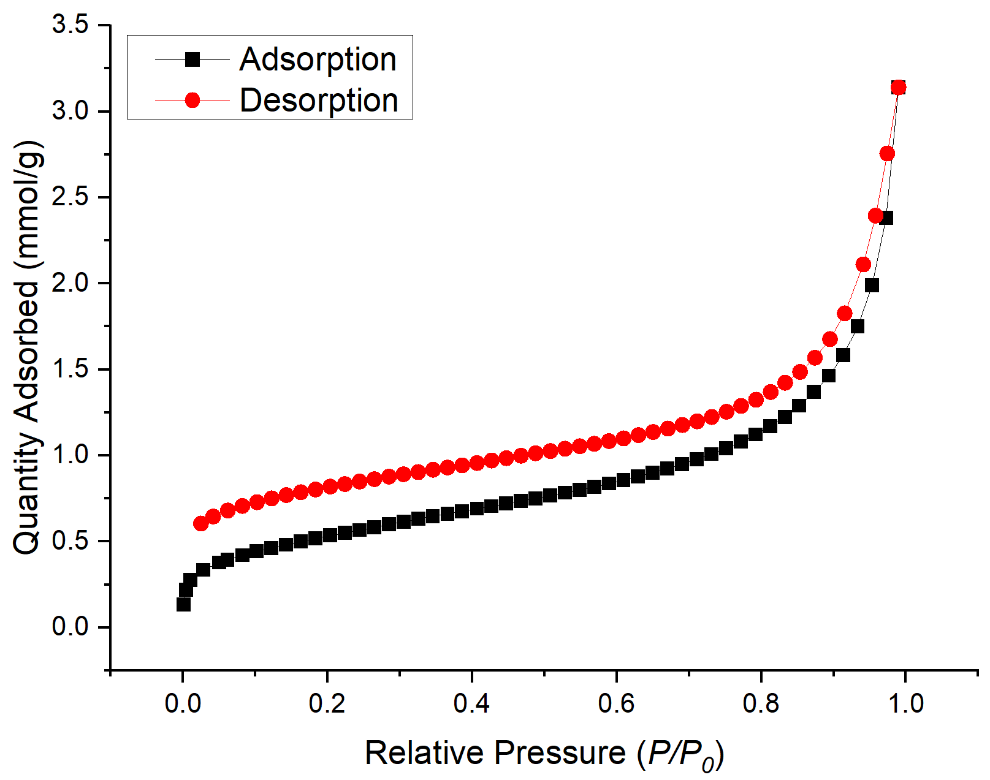


**Figure S58** Nitrogen adsorption isotherm of **35_CMP23_DMF**


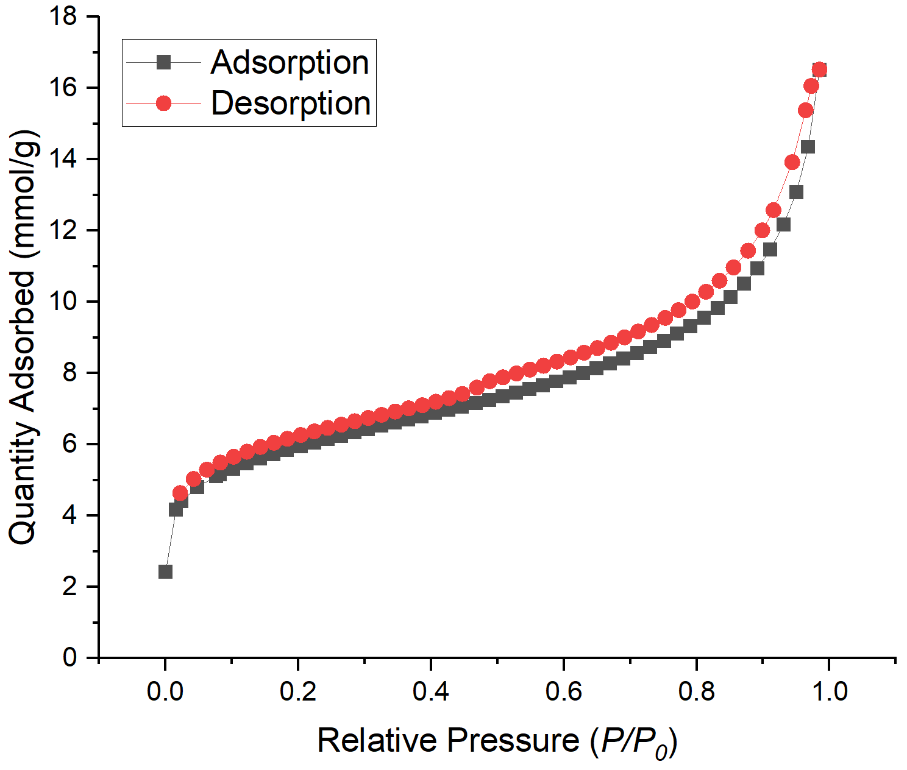


**Figure S59** Nitrogen adsorption isotherm of **36_CMP24_DMSO**


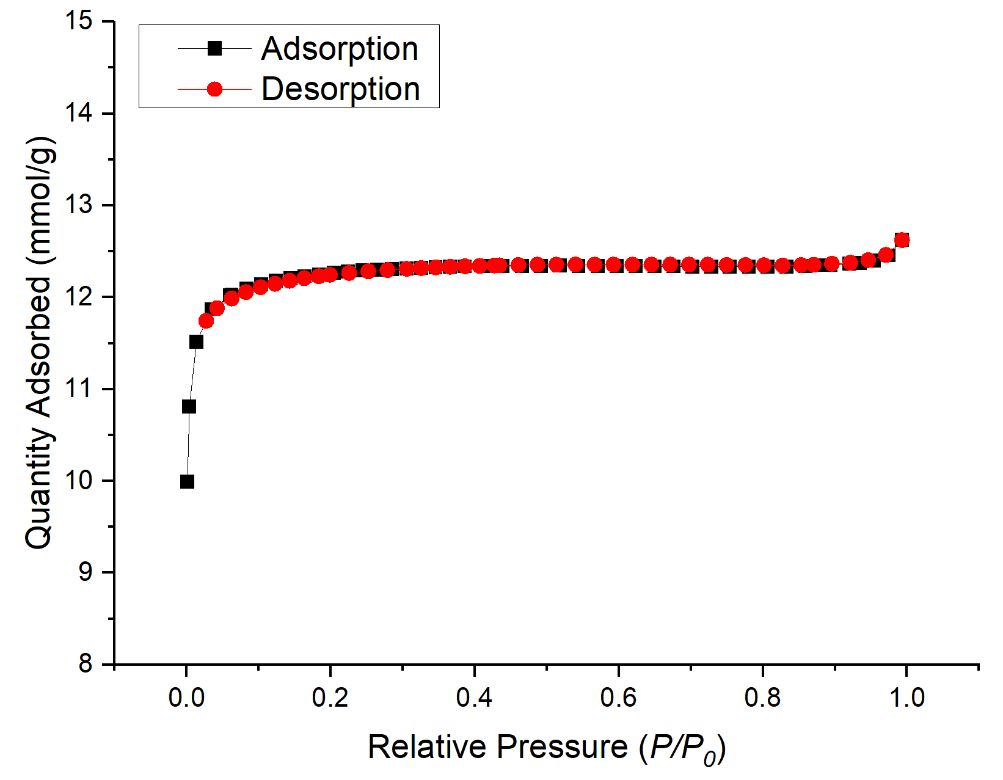


**Figure S60** Nitrogen adsorption isotherm of **37_MOF-801**


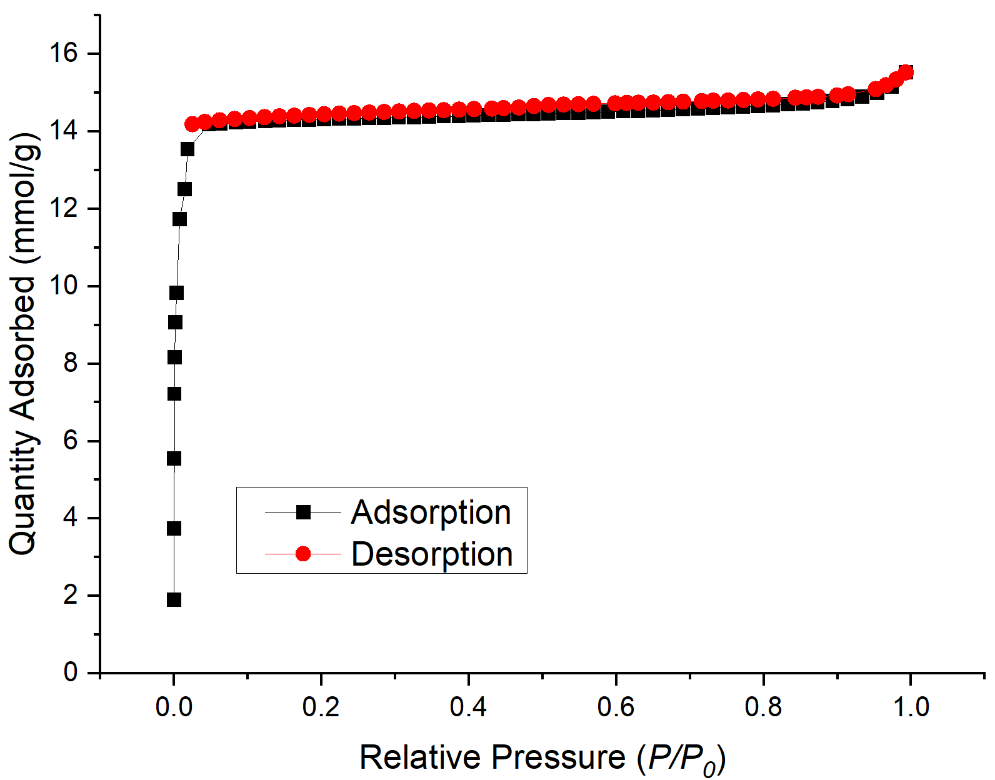


**Figure S61** Nitrogen adsorption isotherm of **38_ZIF-8**


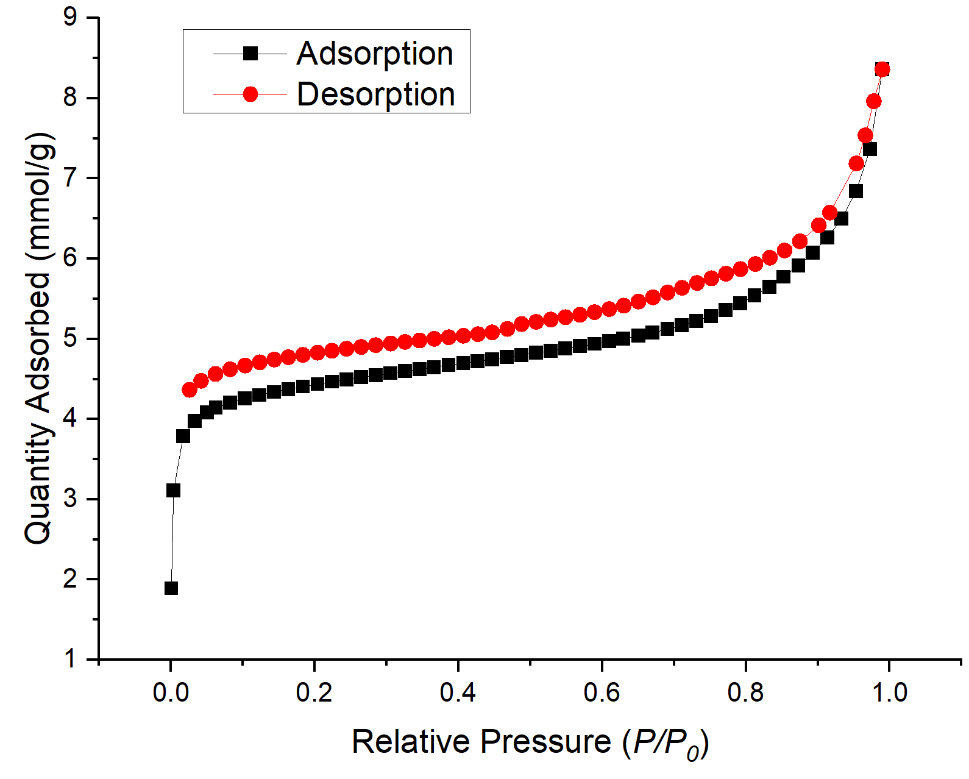


**Figure S62** Nitrogen adsorption isotherm of **39_MIL-53-Al-BPDC**


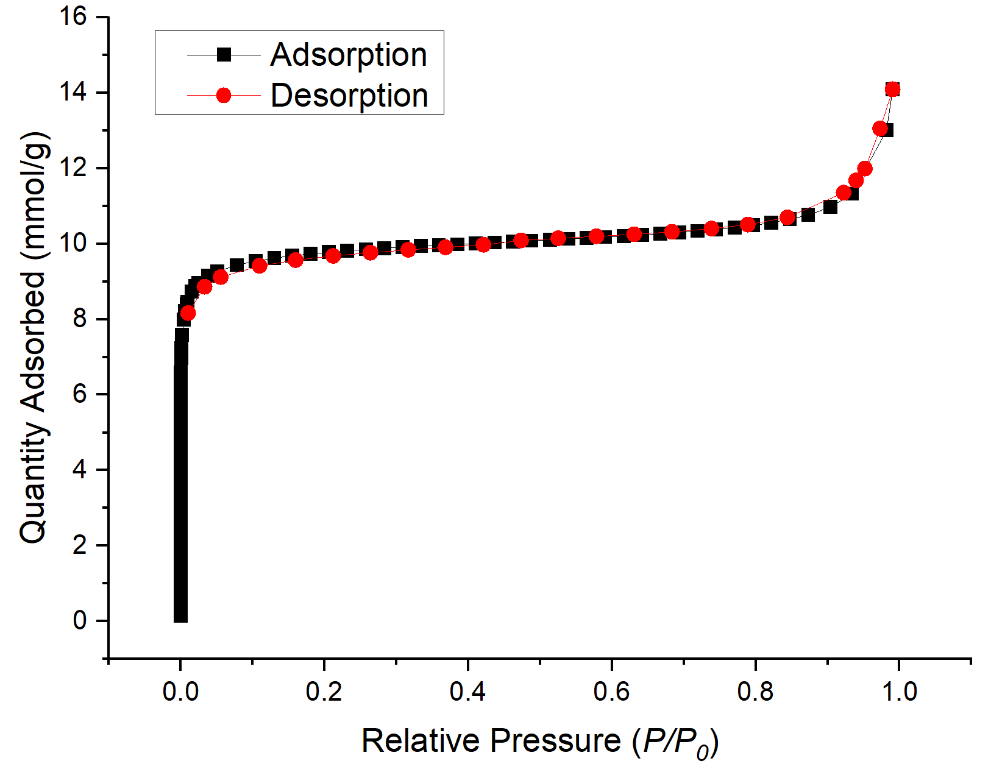


**Figure S63** Nitrogen adsorption isotherm of **40_MIL-68-(Al)**


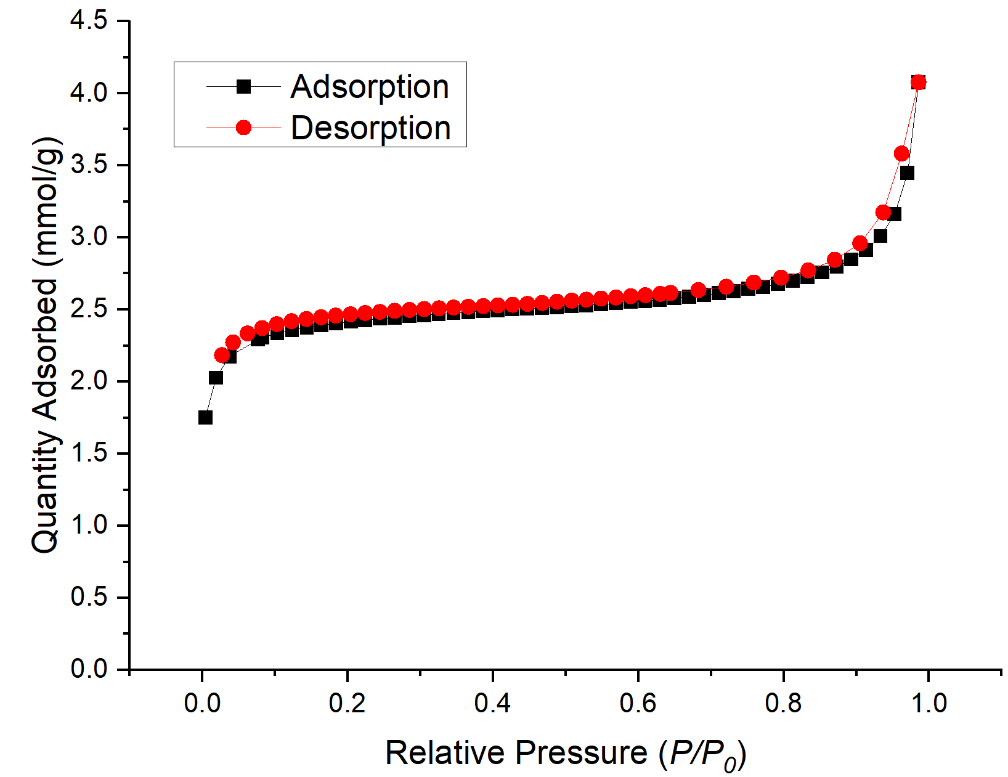


**Figure S64** Nitrogen adsorption isotherm of **41_MIL-101-(Cr)-SO_3_H**


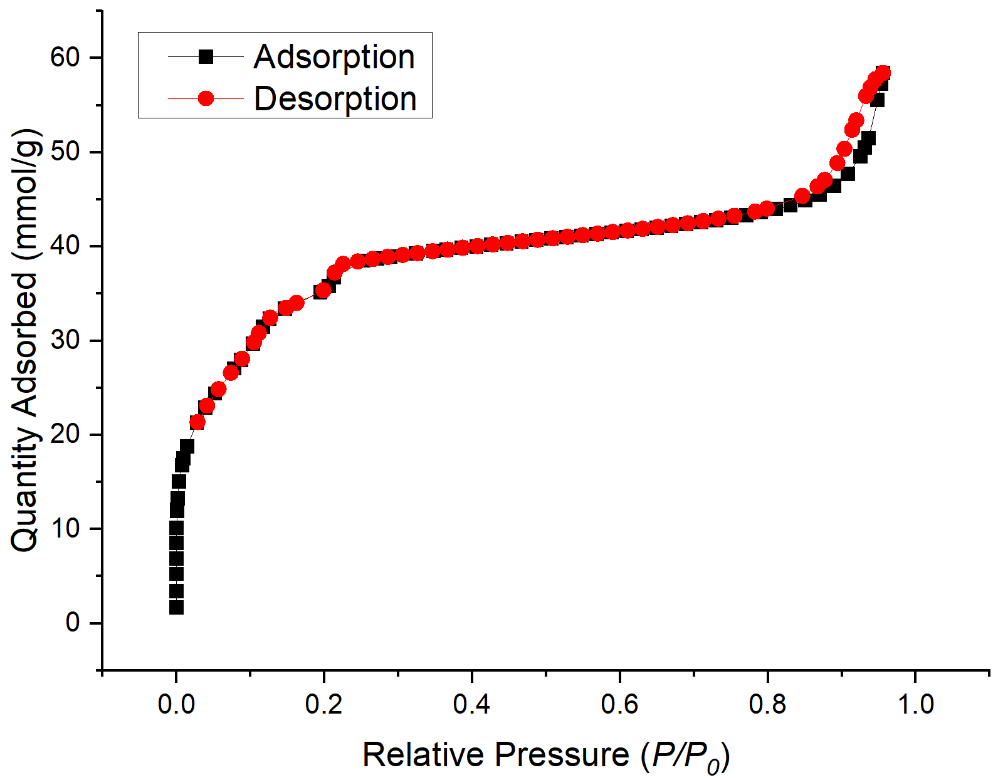


**Figure S65** Nitrogen adsorption isotherm of **42_MIL-101-(Cr)**


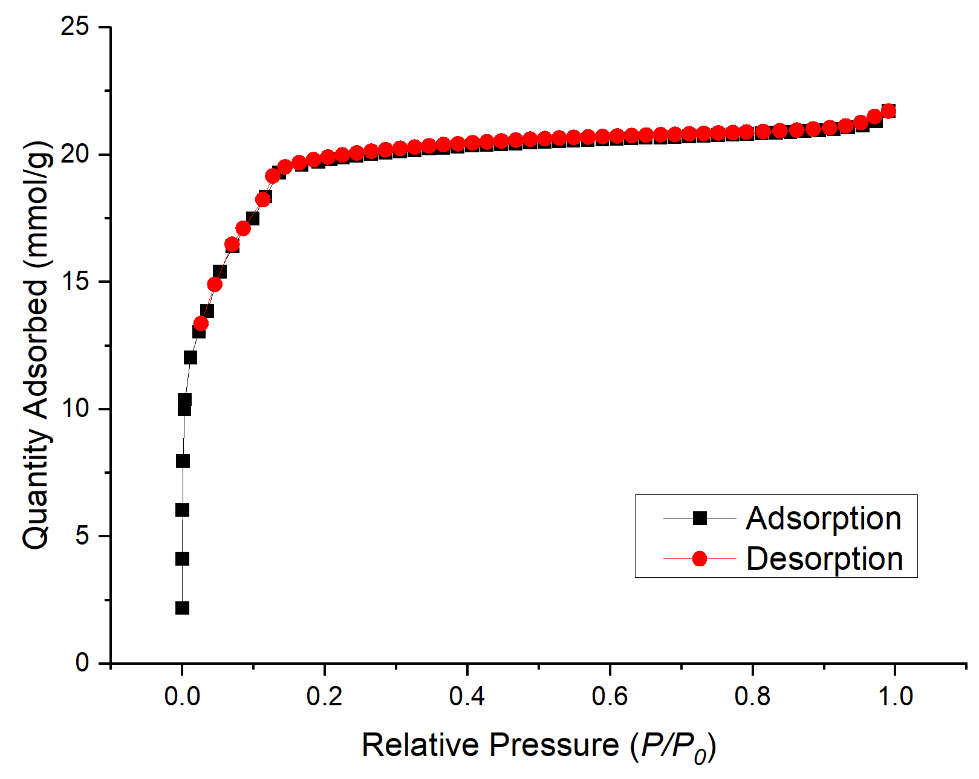


**Figure S66** Nitrogen adsorption isotherm of **43_MIL-100-(Fe)**


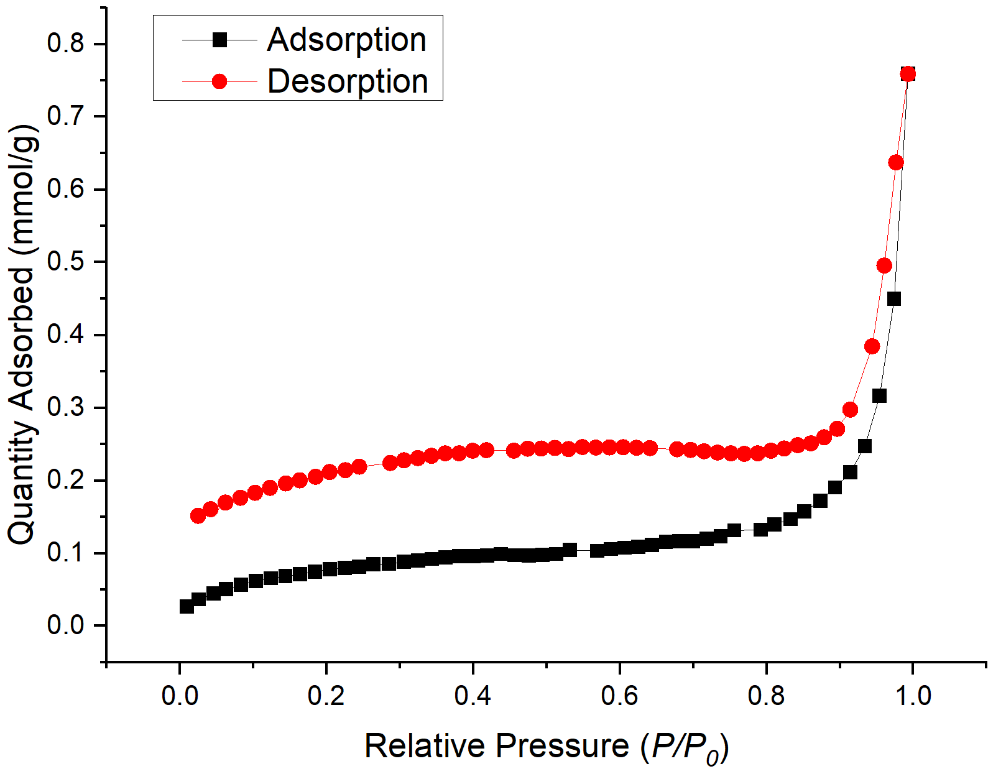


**Figure S67** Nitrogen adsorption isotherm of **44_MIL-53-(Fe)**


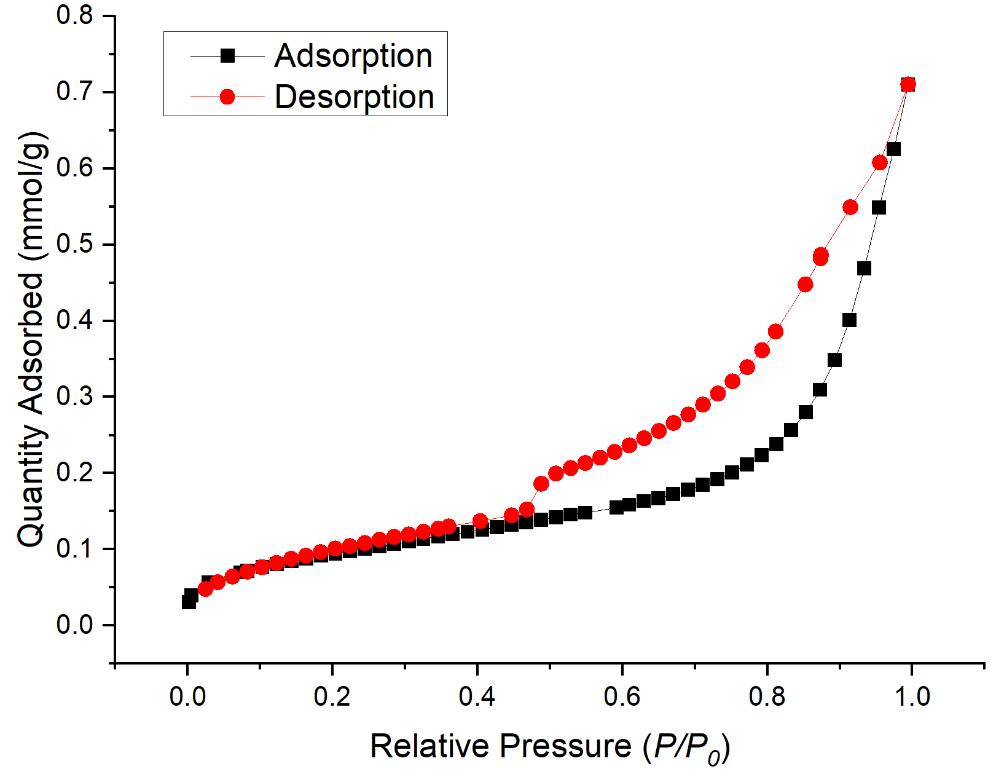


**Figure S68** Nitrogen adsorption isotherm of **45_Ni-BTC**


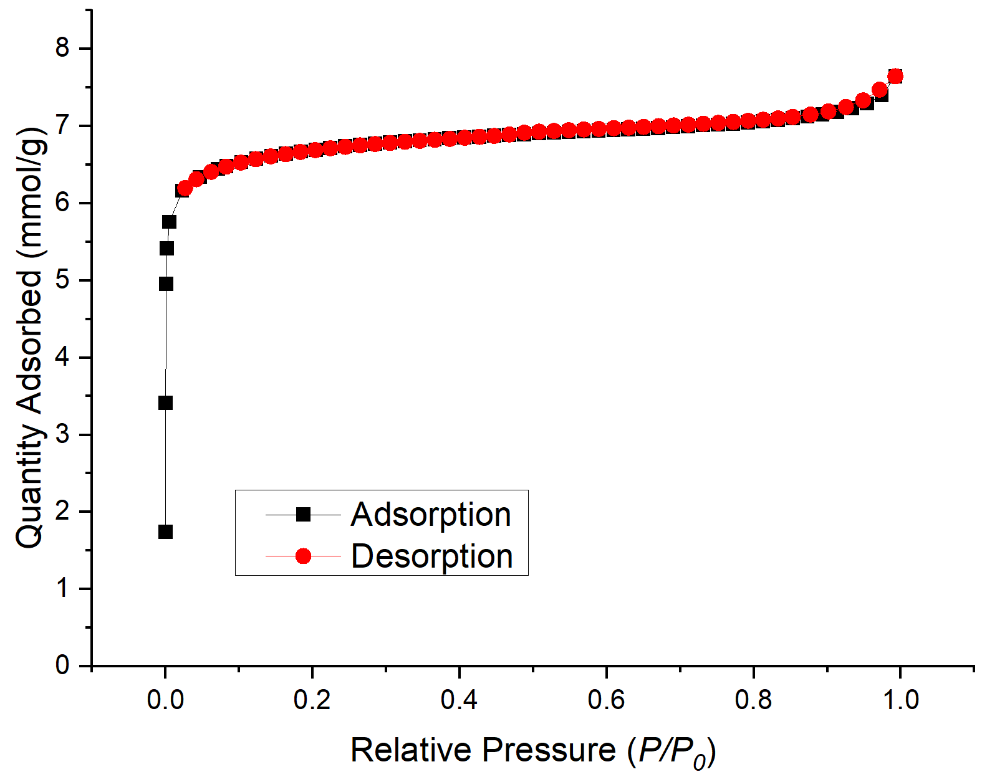


**Figure S69** Nitrogen adsorption isotherm of **46_MIL-125**


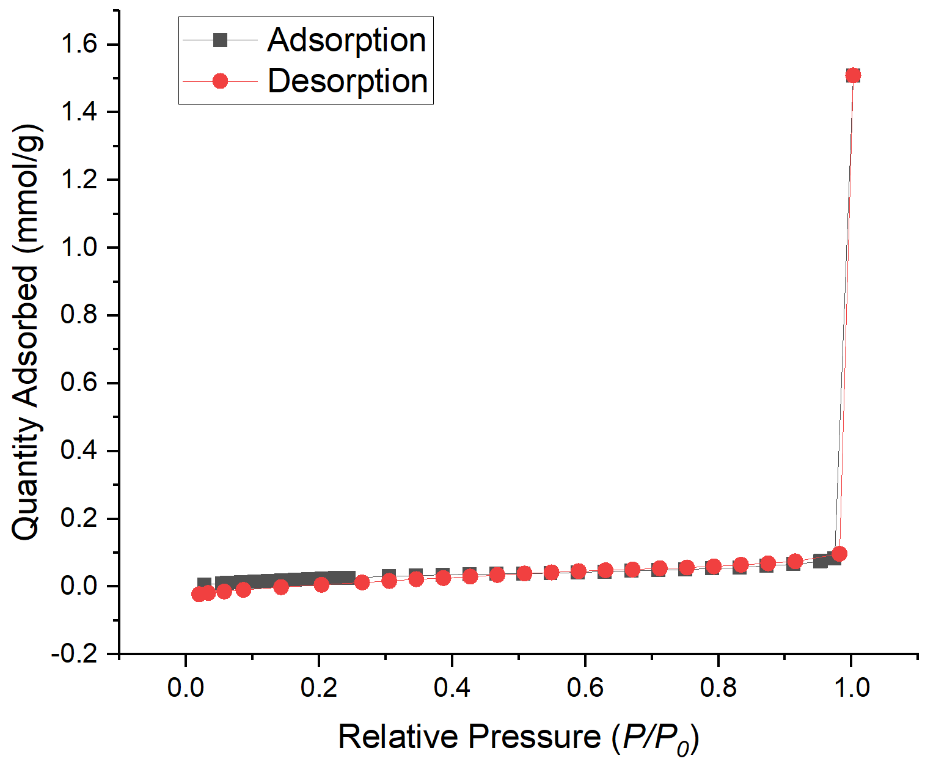


**Figure S70** Nitrogen adsorption isotherm of **47_Ni-PymS**


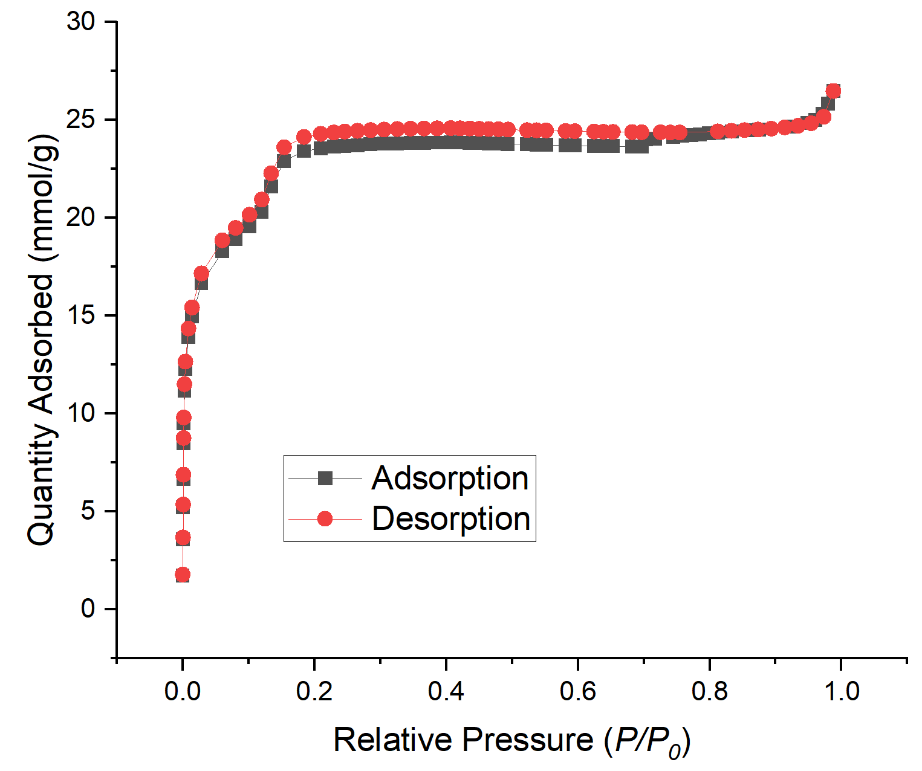


**Figure S71** Nitrogen adsorption isotherm of **48_UiO-67**


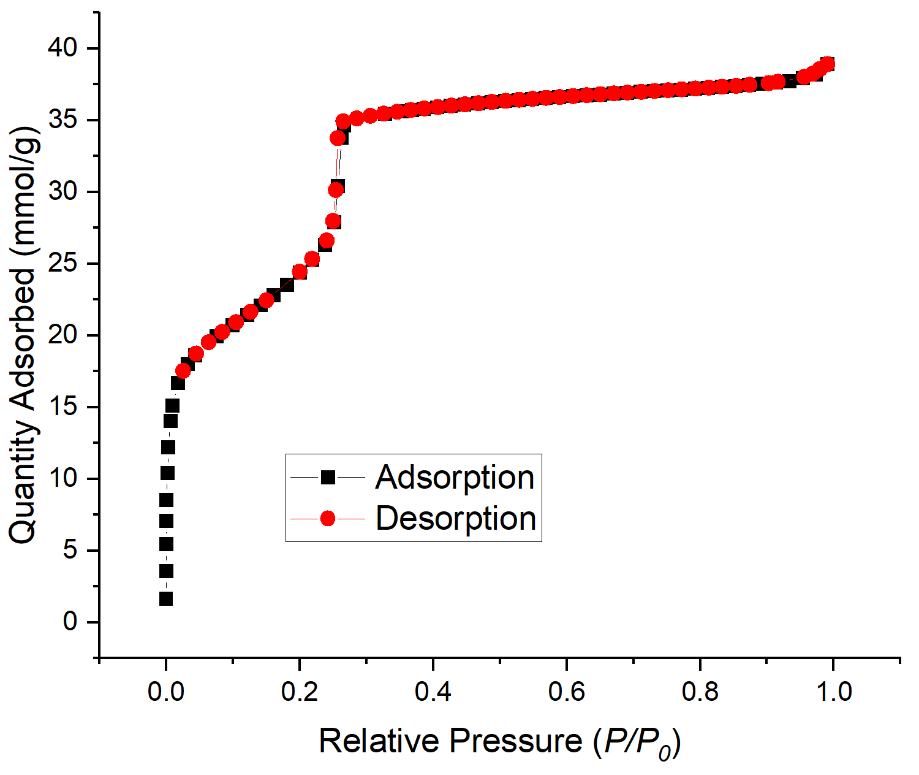


**Figure S72** Nitrogen adsorption isotherm of **49_NU-1000-csq**


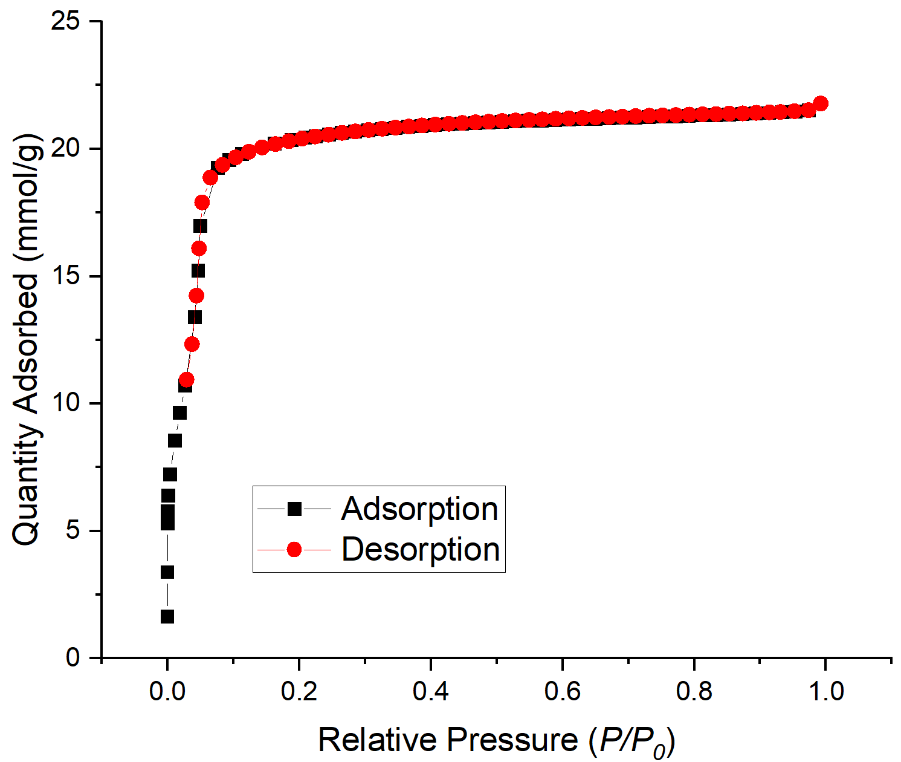


**Figure S73** Nitrogen adsorption isotherm of **50_MOF-808**

# **References**

(1) M. Aitchison, C.; M. Kane, C.; P. McMahon, D.; R. Spackman, P.; Pulido, A.; Wang, X.; Wilbraham, L.; Chen, L.; Clowes, R.; A. Zwijnenburg, M.; Sebastian Sprick, R.; A. Little, M.; M. Day, G.; I. Cooper, A. Photocatalytic Proton Reduction by a Computationally Identified, Molecular Hydrogen-Bonded Framework. *J. Mater. Chem. A* **2020**, *8* (15), 7158–7170. https://doi.org/10.1039/D0TA00219D.

(2) Yuan, Y. D.; Dong, J.; Liu, J.; Zhao, D.; Wu, H.; Zhou, W.; Gan, H. X.; Tong, Y. W.; Jiang, J.; Zhao, D. Porous Organic Cages as Synthetic Water Channels. *Nat. Commun.* **2020**, *11* (1), 4927. https://doi.org/10.1038/s41467-020-18639-7.

(3) Hasell, T.; Chong, S. Y.; Jelfs, K. E.; Adams, D. J.; Cooper, A. I. Porous Organic Cage Nanocrystals by Solution Mixing. *J. Am. Chem. Soc.* **2012**, *134* (1), 588–598. https://doi.org/10.1021/ja209156v.

(4) A. Little, M.; Y. Chong, S.; Schmidtmann, M.; Hasell, T.; I. Cooper, A. Guest Control of Structure in Porous Organic Cages. *Chem. Commun.* **2014**, *50* (67), 9465–9468. https://doi.org/10.1039/C4CC04158E.

(5) Chang, C.-W.; Borne, I.; Lawler, R. M.; Yu, Z.; Jang, S. S.; Lively, R. P.; Sholl, D. S. Accelerating Solvent Selection for Type II Porous Liquids. *J. Am. Chem. Soc.* **2022**, *144* (9), 4071–4079. https://doi.org/10.1021/jacs.1c13049.

(6) Liu, M.; Little, M. A.; Jelfs, K. E.; Jones, J. T. A.; Schmidtmann, M.; Chong, S. Y.; Hasell, T.; Cooper, A. I. Acid- and Base-Stable Porous Organic Cages: Shape Persistence and pH Stability via Post-Synthetic “Tying” of a Flexible Amine Cage. *J. Am. Chem. Soc.* **2014**, *136* (21), 7583–7586. https://doi.org/10.1021/ja503223j.

(7) Furukawa, H.; Gándara, F.; Zhang, Y.-B.; Jiang, J.; Queen, W. L.; Hudson, M. R.; Yaghi, O. M. Water Adsorption in Porous Metal–Organic Frameworks and Related Materials. *J. Am. Chem. Soc.* **2014**, *136* (11), 4369–4381. https://doi.org/10.1021/ja500330a.

(8) Akpinar, I.; Drout, R. J.; Islamoglu, T.; Kato, S.; Lyu, J.; Farha, O. K. Exploiting π–π Interactions to Design an Efficient Sorbent for Atrazine Removal from Water. *ACS Appl. Mater. Interfaces* **2019**, *11* (6), 6097–6103. https://doi.org/10.1021/acsami.8b20355.

(9) Katz, M. J.; Brown, Z. J.; Colón, Y. J.; Siu, P. W.; Scheidt, K. A.; Snurr, R. Q.; Hupp, J. T.; Farha, O. K. A Facile Synthesis of UiO-66, UiO-67 and Their Derivatives. *Chem. Commun.* **2013**, *49* (82), 9449–9451. https://doi.org/10.1039/C3CC46105J.

(10) Zarabadi-Poor, P.; Marek, R. In Silico Study of (Mn, Fe, Co, Ni, Zn)-BTC Metal–Organic Frameworks for Recovering Xenon from Exhaled Anesthetic Gas. *ACS Sustain. Chem. Eng.* **2018**, *6* (11), 15001–15006. https://doi.org/10.1021/acssuschemeng.8b03475.

(11) *Preparation of BiVO4/MIL‐125(Ti) composite with enhanced visible‐light photocatalytic activity for dye degradation - Yang - 2018 - Applied Organometallic Chemistry - Wiley Online Library*. https://onlinelibrary.wiley.com/doi/full/10.1002/aoc.4285 (accessed 2024-11-04).

(12) Feng, Y.; Chen, C.; Liu, Z.; Fei, B.; Lin, P.; Li, Q.; Sun, S.; Du, S. Application of a Ni Mercaptopyrimidine MOF as Highly Efficient Catalyst for Sunlight-Driven Hydrogen Generation. *J. Mater. Chem. A* **2015**, *3* (13), 7163–7169. https://doi.org/10.1039/C5TA00136F.

(13) Jiang, J.; Gándara, F.; Zhang, Y.-B.; Na, K.; Yaghi, O. M.; Klemperer, W. G. Superacidity in Sulfated Metal–Organic Framework-808. *J. Am. Chem. Soc.* **2014**, *136* (37), 12844–12847. https://doi.org/10.1021/ja507119n.

(14) Yang, H.; Li, C.; Liu, T.; Fellowes, T.; Chong, S. Y.; Catalano, L.; Bahri, M.; Zhang, W.; Xu, Y.; Liu, L.; Zhao, W.; Gardner, A. M.; Clowes, R.; Browning, N. D.; Li, X.; Cowan, A. J.; Cooper, A. I. Packing-Induced Selectivity Switching in Molecular Nanoparticle Photocatalysts for Hydrogen and Hydrogen Peroxide Production. *Nat. Nanotechnol.* **2023**, *18* (3), 307–315. https://doi.org/10.1038/s41565-022-01289-9.

(15) Dawson, R.; Laybourn, A.; Khimyak, Y. Z.; Adams, D. J.; Cooper, A. I. High Surface Area Conjugated Microporous Polymers: The Importance of Reaction Solvent Choice. *Macromolecules* **2010**, *43* (20), 8524–8530. https://doi.org/10.1021/ma101541h.

(16) David, E. Evaluation of Na-13X Zeolites Activity in the Catalytic Pyrolysis of Rapeseed Oil Cake to Produce Bio-Oil. *Appl. Catal. Gen.* **2021**, *617*, 118126. https://doi.org/10.1016/j.apcata.2021.118126.

(17) Han, Y.; Larmier, K.; Rivallan, M.; Pirngruber, G. D. Generation of Mesoporosity in H–Y Zeolites by Basic or Acid/Basic Treatments: Towards a Guideline of Optimal Si/Al Ratio and Basic Reagent. *Microporous Mesoporous Mater.* **2024**, *365*, 112906. https://doi.org/10.1016/j.micromeso.2023.112906.

(18) Thomson, K. T. Handbook of Zeolite Science and Technology Edited by Scott M. Auerbach (University of Massachusetts, Amherst), Kathleen A. Carrado (Argonne National Laboratory), Prabir K. Dutta (The Ohio State University). Marcel Dekker, Inc.:  New York, Basel. 2003. Xii + 1184 Pp. $235.00. ISBN 0-8247-4020-3. *J. Am. Chem. Soc.* **2004**, *126* (28), 8858–8859. https://doi.org/10.1021/ja0336067.

(19) *Adsorption Equilibria of Water Vapor on Zeolite 3A, Zeolite 13X, and Dealuminated Y Zeolite | Journal of Chemical & Engineering Data*. https://pubs.acs.org/doi/full/10.1021/acs.jced.5b00927 (accessed 2025-07-07).
